# Supplementary material for: Diabetes ROADMAP: Teaching Guideline Use, Communication, and Documentation When Delivering the Diagnosis of Diabetes
Source: MedEdPORTAL. 2020 Sep 11;16:10959. doi: 10.15766/mep_2374-8265.10959 (PMC7485911; doi:10.15766/mep_2374-8265.10959)
Supplement: Supplementary file 1 — Curriculum Overview.pdfTeaching Guide.pdfROADMAP Presentation.pptxFacilitator Guide.pdfSimulation Resources.pdfAssessment Tools.pdf [file mep_2374-8265.10959-s001.zip › A. Curriculum Overview.pdf]

ROADMAP

CURRICULUM GUIDE

Uniformed Services University of the Health Sciences

MPCRN

MILITARY PRIMARY CARE RESEARCH NETWORK

Table of contents

Introduction ____ 3

How to use this curriculum guide ____ 3

Section 1: Curriculum Overview

Curriculum format ____ 5

Roles and responsibilities ____ 7

Task checklist ____ 8

Objectives and core competencies ____ 9

Section 2: Teaching Guide

Overview ____ 11

Learning objectives ____ 12

Teaching slides ____ 13

HANDOUT: CLINICAL PRACTICE APPLICATION ____ 28

HANDOUT: CLINICAL DOCUMENTATION APPLICATION ____ 29

Section 3: Facilitator Guide

Overview ____ 31

Role play instructions ____ 33

Facilitator notes ____ 36

HANDOUT: ROLE PLAY SCENARIOS ____ 37

HANDOUT: ROLE PLAY OBSERVATION ____ 38

Section 4: Assessment Tools

Did ROADMAP improve practice? ____ 40

HANDOUT: LEARNER KNOWLEDGE CHECK ____ 41

Learner knowledge check key 42

HANDOUT: CURRICULUM EVALUATION 44

Section 5: Simulation Resources

Overview 46

Practice partner instructions 48

Instructions for learners 51

Clinical rehearsal feedback session guide 52

HANDOUT: PRACTICE PARTNER INFORMATION 53

HANDOUT: PRACTICE PARTNER FEEDBACK FORMS 55

Character case set 56

Authors include: Christy J.W. Ledford, Lauren A. Cafferty, Heather A. Rider, Dean A. Seehusen, Paul F. Crawford, Jasmyne J. Womack, Angela B. Seehusen, Tyler Rogers, Stephanie Fulleborn, Erik Clauson, Steven Trigg, and Christopher C. Ledford.

Disclaimer. The views expressed within this publication do not represent those of the authors and do not reflect the official position of the U.S. Air Force, U.S. Army, Uniformed Services University of the Health Sciences, the Department of Defense, or the U.S. Government at large.

The ROADMAP curriculum is designed as part of a grant to Uniformed Services University of the Health Sciences by Congressionally Directed Medical Research Programs (DM 150083).

Introduction

Many newly-diagnosed patients have limited prior medical knowledge of diabetes. The strongest predictor of how patients make sense of symptoms, actions, and consequences is their perception of how diabetes was explained to them. During that first diagnosis, clinicians should name the disease, provide background information about the disease, and assess the patient's response. Clinicians can influence patient self-management through discussions about how a patient can control disease outcomes through self-management behaviors. It is especially critical for clinicians to share information at the point of diagnosis because patients may go home and search for information online which can lead to information overload following diagnosis. Patients also expect clinicians to provide information and emotional support when they deliver challenging news.

Researchers investigating disparities among patients with diabetes repeatedly argue for intervention at multiple levels, particularly focusing on communication skills. Diabetes ROADMAP (Responding to the Opportunity to Adapt the Diagnosis to Motivate and Activate Patients) provides that intervention through clinician training because patients consistently cite their healthcare provider as their preferred source of information.

How to use this curriculum guide

Diabetes ROADMAP is a 2 or 3 hour curriculum (dependent on the usage of the optional Section 5) that teaches medical decision making, interpersonal communication, and clinical documentation in the context of prediabetes and diabetes. The aim of Diabetes ROADMAP is to prepare clinicians for the challenge of talking to patients about a new diabetes or prediabetes diagnosis. This curriculum can be used as undergraduate medical education, graduate medical education, or continuing medical education.

This guide includes curricular content along with details for implementation. It is divided into 5 sections.

Section 1: Curriculum Overview includes information on implementation and roles and responsibilities.

Section 2: **Teaching Guide** provides detailed presentation information and learner handouts for the large group.

Section 3: Facilitator Guide provides instructions and handouts for a small group role play activity.

Section 4: Assessment Tools provides options for tools to assess how well learning objectives are met and to provide additional feedback to learners.

Section 5: Simulation Resources provides an *optional* addition to the standard format. Materials and directions for adding patient simulation are included.

CURRICULUM OVERVIEW

Uniformed Services University of the Health Sciences

Authors include: Christy JW Ledford, Heather Rider, and Lauren Cafferty.

Curriculum format

TEACHING SESSION

The ROADMAP teaching session has three distinct lectures:

1. **Medical decision making**
   This lecture presents the 2019 guidelines for screening and treatment of prediabetes and type 2 diabetes.
2. **Interpersonal communication**
   This lecture focuses on how to communicate with patients during medical decision making.
3. **Clinical documentation**
   The final lecture presents how to tell the patient's story in the health record.

The first and second lectures (medical decision making and interpersonal communication) are grouped together, followed by a role play activity in small groups. After the activity is complete, learners return together to review the final lecture, clinical documentation.

The content of the teaching session can be found in the Teaching Guide (Section 2 beginning on page 10).

ROLE PLAY

Following the first two lectures, role play in a small group focuses on brainstorming what words to use in a clinical encounter and gives learners a non-threatening space to discover how they can best apply what they just learned in the lectures.

In groups of three to five learners plus a facilitator, group members can practice each microskill, or step, in communicating a diagnosis:

1. **Explain the diagnosis**: Name the condition, clearly explain what it is, and check for understanding.
2. **Explore patient perceptions**: Check for shared meaning, including severity and susceptibility.
3. **Establish goal for today's appointment**: Knowing diabetes is a chronic condition, determine what you and the patient can accomplish today.
4. **Elicit patient preferences**: Include the patient in decision making about treatment options and assessing behavior change facilitators and barriers.

This applied exercise allows learners to adapt the learning into their own vernacular and to hear feedback from their peers. The Facilitator Guide (Section 3 beginning on page 30) provides more detail on the role play activity.

CLINICAL PRACTICE

Learning does not end at the culmination of the teaching session. The goal of medical education is to improve clinical practice. In the weeks following the curricular intervention, it is highly likely that as a primary care provider, the learner will apply the skills of delivering a diabetes diagnosis in a patient encounter. When this occurs, the learner should complete the CLINICAL PRACTICE APPLICATION (p. 28). This

worksheet outlines the four steps of delivering the diagnosis and provides an example of how the clinician should note the encounter. Teaching preceptors can walk through these steps with the learner when they encounter a patient with a new prediabetes or diabetes diagnosis.

In addition to graduate medical education, this curriculum can be used with staff physicians and mid-level providers for formal Continuing Medical Education credits. For that audience, the curriculum can be shorter to accommodate clinical schedules and responsibilities. Within the overall structure, the time per activity can be abbreviated, or extended to fit your program's needs.

SCHEDULE

Programmatic overview of curriculum as graduate medical education (2 hours)

**Activity**	**Objective**	**Format**	**Time**
Medical decision making lecture	To present the current* guidelines for screening and treatment of prediabetes and type 2 diabetes.	Large group	15 mins
Interpersonal communication lecture	To teach how clinicians can facilitate patient conversations that lead to shared meaning of the diabetes diagnosis.		25 mins
Role play	To apply and practice skills in a supportive learning climate of observation and feedback.	Small group	50 mins
Clinical documentation lecture	To demonstrate how to incorporate information from the four steps into the patient's story, as documented in the health record.	Large group	15 mins
With personal practice and application…			
Clinical practice application	To reflect on application of skills utilized in clinical practice.	One on one	10 mins

*The 2019 American Diabetes Association (ADA) guidelines are presented in this curriculum guide.

WHAT COMES NEXT?

Diabetes ROADMAP is a curriculum focused on creating shared meaning and understanding with the patient and the healthcare team. The ideas presented in this curriculum are applicable beyond metabolic disorder. Encourage your learners to consider how shared meaning and understanding plays a role in the management of other chronic diseases within your patient population. Skills learned in this curriculum could be applied to talking with patients about hypertension, obesity, and other chronic conditions.

Roles and responsibilities

This curricular model requires educators to fill administrative, teaching, and facilitating roles. You can choose how many educators fill these roles and share responsibilities. One potential task-sharing framework is for a lead educator to take the administrative and training role, another set of educators to be teachers, and another set of educators to be facilitators. Collaboration is encouraged within and outside the program walls.

ADMINISTRATIVE/TRAINING ROLE

- Resources the curricular intervention
- Assigns educator roles
- Implements curricular intervention details

This role is best matched to individuals who are interested in the teaching content, who are detail-oriented, who have personal connections within and outside the residency, and who have a good understanding of scheduling.

TEACHING ROLE

- Prepares materials for the lectures
- Delivers the lecture content to large group

Content knowledge and credibility with learners are key. For the communication lecture, behavioral health or communication experts can add value. For the clinical documentation lecture, a clinician who has extensive experience with your hospital system's electronic health record can contribute details about the specific interface for documentation.

FACILITATOR ROLE

- Leads the small group activity

Facilitators don't need to be content experts. This role is about group dynamics and interpersonal management. Social skills are important for facilitation. Facilitators must be individuals who can keep a group on task and who are skilled at gentle interruption and group direction. Consider who the learners are when choosing the facilitators – who will learners allow to direct their discussion? A person from outside the residency or clinic can help learners feel at ease taking risks in the small group.

Task checklist

Tasks	NOTES
**Prior to day of curricular intervention**<br>☐ Schedule space for large group<br>☐ Schedule space for small groups<br>☐ Identify teachers<br>☐ Identify small group facilitators<br>☐ Assign tasks/roles to team<br>☐ Invite learners<br>☐ Print guides for teachers (Section 2) and facilitators (Section 3)<br>☐ Print handouts<br>☐ Teaching PowerPoint slide notes (optional)<br>☐ Teaching reference list (optional) (p. 27)<br>☐ ROLE PLAY SCENARIOS (p. 37)<br>☐ ROLE PLAY OBSERVATION (p. 38)<br>☐ CLINICAL PRACTICE APPLICATION and CLINICAL DOCUMENTATION GUIDE (pp. 28-29, can be printed double-sided, additional 10 per preceptor)<br>☐ LEARNER KNOWLEDGE CHECK (p. 41)<br>☐ CURRICULUM EVALUATION (p. 44)<br>☐ Train small group facilitators<br>☐ Review lecture content with teachers<br>☐ Tour space to understand flow	
**Day of curricular intervention**<br>☐ Assign small groups prior to first lecture<br>☐ Collect LEARNER KNOWLEDGE CHECK at end of clinical documentation lecture<br>☐ Collect CURRICULUM EVALUATION after collecting LEARNER KNOWLEDGE CHECK<br>☐ Distribute CLINICAL PRACTICE APPLICATION worksheets to preceptors	
**In six weeks following day of curricular intervention**<br>☐ Precept through CLINICAL PRACTICE APPLICATION worksheet	

Objectives and core competencies

Learners will be able …

- to list and describe the current American Diabetes Association (ADA) guidelines on screening, diagnosis, and treatment of prediabetes and type 2 diabetes;
- to recognize the connection between the diagnosis moment and potential for patient behavior change;
- to demonstrate how to establish shared meaning when communicating a new diabetes diagnosis; and
- to document a meaningful diabetes diagnosis in the patient health record.

These objectives align with the Accreditation Council for Graduate Medical Education (ACGME) core competencies of patient care (PC) and interpersonal and communication skills (C) within the following Family Medicine sub-competencies:

PC-2. Cares for patients with chronic conditions.

C-1. Develops meaningful, therapeutic relationships with patients and families.

C-2. Communicates effectively with patients, families, and the public.

C-3. Develops relationships and effectively communicates with physicians, other health professionals, and health care teams.

C-4. Uses technology to optimize communication.

This curriculum contributes to learners' ability to achieve the following Entrustable Professional Activities (EPA) for Family Medicine:

EPA-1. Provide a usual source of comprehensive, longitudinal medical care for people of all ages.

EPA-7. Diagnose and manage chronic medical conditions and multiple co-morbidities.

EPA -15. Develop trusting relationships and sustained partnerships with patients, families and communities.

EPA -17. In the context of culture and health beliefs of patients and families, use the best science to set mutual health goals and provide services most likely to benefit health.

On the day of the ROADMAP curricular intervention, learners participate in a teaching session and small group activity. The ROADMAP curriculum also includes structured reflection and feedback resources for educators and learners to incorporate the skills of delivering a diabetes diagnosis into clinical practice in the weeks that follow the curricular intervention.

This curriculum takes a learner-centered approach, which promotes learners' participation in setting goals and priorities; encourages sharing of personal and professional experiences related to the context; fosters a supportive learning climate that promotes risk-taking; engages learners in interactive experiences; and attends to relationship-building among participants.
