# Supplementary material for: Diabetes ROADMAP: Teaching Guideline Use, Communication, and Documentation When Delivering the Diagnosis of Diabetes
Source: MedEdPORTAL. 2020 Sep 11;16:10959. doi: 10.15766/mep_2374-8265.10959 (PMC7485911; doi:10.15766/mep_2374-8265.10959)
Supplement: Supplementary file 1 — Curriculum Overview.pdfTeaching Guide.pdfROADMAP Presentation.pptxFacilitator Guide.pdfSimulation Resources.pdfAssessment Tools.pdf [file mep_2374-8265.10959-s001.zip › B. Teaching Guide.pdf]

ROADMAP

TEACHING GUIDE

Uniformed Services University of the Health Sciences

MPCRN
MILITARY PRIMARY CARE RESEARCH NETWORK

Authors include: Christy JW Ledford, Dean Seehusen, Tyler Rogers, Stephanie Fulleborn, Erik Clauson, Steven Trigg, and Christopher Ledford.

Overview

The ROADMAP teaching session has three distinct lectures: medical decision making and interpersonal communication in the initial large group presentation and then clinical documentation following the small group activity. The first lecture presents guidelines for screening and treatment of prediabetes and type 2 diabetes. The second lecture focuses on how to communicate with patients during the medical decision making that follows a new diagnosis. Throughout the lecture, we have incorporated patient quotes from formative research so that learners can hear about communication challenges through the patients' voices. A diagnosis can only be truly meaningful is if it is shared among the primary care provider, the patient, and the healthcare team. The final lecture presents how to tell the patient's story in the health record. It is critical to teach the learner how to appropriately document the diagnosis discussion to facilitate continuity of care and informed medical decision making. This section of didactic instruction covers appropriate coding of the encounter, the characteristics of a quality clinical note, and where in the note to document the four steps of delivering the diagnosis.

SCHEDULE

Programmatic overview of curriculum as graduate medical education (2 hours)

Activity	Objective	Format	Time
Medical decision making lecture	To present the current* guidelines for screening and treatment of prediabetes and type 2 diabetes.	Large group	15 mins
Interpersonal communication lecture	To teach how clinicians can facilitate patient conversations that lead to shared meaning of the diabetes diagnosis.		25 mins
Role play	To apply and practice skills in a supportive learning climate of observation and feedback.	Small group	50 mins
Clinical documentation lecture	To demonstrate how to incorporate information from the four steps into the patient's story, as documented in the health record.	Large group	15 mins
With personal practice and application…			
Clinical practice application	To reflect on application of skills utilized in clinical practice.	One on one	10 mins

*The 2019 American Diabetes Association (ADA) guidelines are presented in this curriculum guide.

From the teaching session, we recommend two handouts for learners:

1. Teaching slides, printed as PowerPoint notes pages to allow learners to take additional notes or to keep for reference, and
2. Reference list from the teaching slides (p. 27) for additional information.

Learning objectives

Diabetes ROADMAP (Responding to the Opportunity to Adapt the Diagnosis to Motivate and Activate Patients) provides clinician training because patients consistently cite their healthcare provider as their preferred source of information. Our aim is to prepare clinicians for the challenge of talking to patients about a new diabetes or prediabetes diagnosis. This curriculum targets four learning objectives.

Learners will be able …

- to list and describe the current American Diabetes Association (ADA) guidelines on screening, diagnosis, and treatment of prediabetes and type 2 diabetes;
- to recognize the connection between the diagnosis moment and potential for patient behavior change;
- to demonstrate how to establish shared meaning when communicating a new diabetes diagnosis; and
- to document a meaningful diabetes diagnosis in the patient health record.

Diabetes ROADMAP is a 2 or 3 hour (dependent on the usage of the optional Section 5) curricular intervention that teaches medical decision making, interpersonal communication, and clinical documentation in the context of prediabetes and diabetes. On the day of the ROADMAP curricular intervention, learners participate in a teaching session and small group activity. The ROADMAP curriculum also includes structured reflection and feedback resources for educators and learners to incorporate the skills of delivering a diabetes diagnosis into clinical practice in the weeks that follow the curricular intervention.

The following teaching guide presents the didactic material describing medical decision making, interpersonal communication, and clinical documentation in the context of prediabetes and diabetes.

The *teaching* role prepares materials for the lectures and delivers the lecture content. Content knowledge and credibility with learners are key.

Individuals who fill the teaching role will:

- Review and adapt teaching slides
- Deliver lecture on medical decision making
- Deliver lecture on interpersonal communication
- Deliver lecture on clinical documentation

Teaching slides

This section presents slide-by-slide teaching content along with notes for the teacher. For each teaching slide, the slide content is within the box. For some slides, potential transition language is included above the box in italicized font within quotes. Below each box are notes for the teacher to give more information about the theory and evidence that underlies each slide.

Slide 1

Title slide

Slide 2

Learning objectives

- To list and describe the current ADA guidelines on screening, diagnosis, and treatment of prediabetes and type 2 diabetes
- To identify the relationship between the diagnosis moment and potential for patient behavior change
- To demonstrate how to establish shared meaning when communicating a new diabetes diagnosis
- To document a meaningful diabetes diagnosis in the patient health record

Notes:

MEDICAL DECISION MAKING LECTURE

Slide 3

Screening and diagnosis guidelines

Notes: All guidelines are current as of the publication of the 2019 Standards of Medical Care in Diabetes.[1]

Slide 4

Diagnostic criteria for T2DM (ADA)

- FPG ≥126 mg/dL. Fasting is defined as no caloric intake for at least 8 hours
- Classic symptoms of hyperglycemia or hyperglycemic crisis and a random plasma glucose ≥200 mg/dL
- A1C ≥6.5%
- 2-h PG ≥200 mg/dL during an 75g GTT

Notes:

Slide 5

Diagnostic criteria for T2DM (ADA)

- Diagnosis requires two abnormal test results from the same sample or in two separate test samples
- If using two separate test samples, the repeat test should be performed without delay

Notes:

Slide 6

[Potential transition] "The American Diabetes Association (ADA) presents definitional guidelines to identify individuals with prediabetes but advocates against considering prediabetes as a 'clinical entity.' Instead, the ADA presents prediabetes as an increased risk factor for T2DM and cardiovascular disease."

Diagnostic criteria for prediabetes (ADA)

- In 1997 and 2003, the Expert Committee on the Diagnosis and Classification of Diabetes Mellitus of the ADA recognized a group of individuals whose glucose levels did not meet the criteria for diabetes but were too high to be considered normal
- A1C 5.7–6.4%
- FPG of 100 mg/dl to 125 mg/dl also acceptable
- 2H GTT results of 140 mg/dl to 199 mg/dl
- Prediabetes should not be viewed as a clinical entity in its own right but rather as an increased risk for diabetes

Notes:

Slide 7

Screening criteria (ADA)

- Testing should be considered in overweight or obese (BMI ≥25 kg/m2 or ≥23 kg/m2 in Asian Americans) adults who have one or more of the following risk factors:
  - A1C ≥5.7% (39 mmol/mol), IGT, or IFG on previous testing
  - first-degree relative with diabetes
  - high-risk race/ethnicity (e.g., African American, Latino, Native American, Asian American, Pacific Islander)
  - women who were diagnosed with GDM
  - history of CVD
  - hypertension (≥140/90 mmHg or on therapy for hypertension)
  - HDL cholesterol level <35 mg/dL (0.90 mmol/L) and/or a triglyceride level >250 mg/dL (2.82 mmol/L)
  - women with polycystic ovary syndrome
  - physical inactivity
  - other clinical conditions associated with insulin resistance (e.g., severe obesity, acanthosis nigricans)

Notes: When learners see this list, they will see that most American patients qualify for screening.

Slide 8

Screening criteria (continued)

- For all patients, testing should begin at age 45 years.
- If results are normal, testing should be repeated at a minimum of 3-year intervals, with consideration of more frequent testing depending on initial results and risk status
- Those with prediabetes should be tested yearly

Notes:

Slide 9

[Potential transition] "When we look at prediabetes as a 'risk factor,' this conversation needs to carefully talk through treatment options. This conversation can help establish a habit of shared decision making."

Treatment options for prediabetes (ADA)

- Intensive Lifestyle Modification (Lose 7% of body weight; >= 150 minutes per week of exercise)
  - Delay T2DM onset by an average of 11 years
  - Reduce incidence of T2DM by 20%
- Metformin
  - Delay T2DM onset by an average of 3.4 years
  - Reduce incidence of T2DM by 8%
- Bariatric surgery
  - If BMI > 40
  - As low as BMI = 30 if weight loss not achieved with reasonable non-surgical means

Notes: The ADA does not define "reasonable non-surgical means."

INTERPERSONAL COMMUNICATION LECTURE

Slide 10

[Potential transition] "This curriculum draws from the theory that the moment of diagnosis is a time when patients can be motivated to change their behavior. But to leverage that opportunity, clinicians need to be intentional about how they deliver the diagnosis."

> ### What is ROADMAP?
> *Responding to the Opportunity to Adapt the Diagnosis to Motivate and Activate Patients*

Notes:

Slide 11

> ### ROADMAP
> - What is a shared meaningful diagnosis?
> - Four steps of delivering the diabetes diagnosis

Notes: The content of ROADMAP is setting the stage for the role of meaning in diagnosis and the four steps of delivery. Throughout the lecture, we have incorporated patient quotes from our formative research so that learners can hear about interpersonal communication challenges through the patients' voice.

Slide 12

[Potential transition] "I want to share some patient stories as we go here. These first two are a simple reminder of how this is a psychologically challenging appointment for a patient. You have likely delivered this diagnosis repeatedly, but this is the one time this patient will hear this news."

> ### Female patient with T2DM
> ***"I think, in the beginning, [finding out I had diabetes] was… it was a wake-up call that I needed at the time, and I knew that I needed to take care of myself…It was the kick I needed to get myself in gear, yeah."***

Notes: This quote sets the stage for how powerful a diagnosis can be.

Slide 13

Shared meaning and the moment of diagnosis

- What does diabetes mean to a person?
  - severity + susceptibility

Notes: When patients don't perceive a disease's threat, they respond to clinician cues differently. For instance, when a patient does not think they'll ever get cancer, when they hear a nutritionist talk about how eating vegetables is associated with preventing cancer, they won't pay attention to it or start eating more vegetables. In the same way, if patients don't perceive the threat of diabetes, they aren't likely to hear messages that talk about how to reduce their chances of developing diabetes.

How does the patient perceive the threat of the disease?

Threat has two parts: severity + susceptibility[2]

Severity = I might think I'm going to develop a disease but not think it's a big deal.

Susceptibility = I might think a disease is really scary but not think I'll ever get it.

Where do we form our perceptions of threat? Seeing people around us, especially family; hearing people around us tell stories; the media (news, entertainment, social media).

Slide 14:

Male patient with T2DM

Interviewer: "*What was your first thought when they told you that [you had diabetes]?"*
Patient: "*You know, I wasn't surprised because my father had diabetes. And I used to watch him take his insulin… his shots. So I had seen him that way for like thirty years, and my mom used to tell me there was always a possibility that I would end up with diabetes. So it was kind of like… I was waiting for it."*

Notes: Quote included as an example of susceptibility.

Slide 15

Male patient with prediabetes

"I didn't have any type of, uh… I wasn't getting up in the middle of the night, going to the bathroom. I wasn't thirsty or anything like that. So that's why I was kind of alarmed when they told me I was pre-diabetic, and nobody in my family ever had diabetes."

Notes: Quote included as example of no perception of susceptibility.

Slide 16

Shared meaning and the moment of diagnosis

- What does diabetes mean to a person?
    - severity + susceptibility
- Why does meaning matter at the moment of diagnosis?
    - When you tell a patient they have a disease, what has happened to their perceived susceptibility?
    - So what role does severity play?

Notes: When you tell a patient they have a disease, what has happened to their perceived susceptibility? If they knew they were susceptible, if their mom and dad both had diabetes, their expectation is met. However, if they didn't think they were susceptible, they experience a jarring dissonance. This can create an emotional response that overwhelms the patient.

So, what role does severity play?[3,4] People are more likely to change their behavior if they think severity is high. For instance, someone, who thinks it's likely they'll develop diabetes but also saw their mom live a pretty normal, unaffected life with the disease, isn't likely to change their behavior. Research studies also reveal that patients who have been told they have prediabetes are uncertain about the seriousness of the condition. Large-scale interventions designed to delay the onset of T2DM emphasize communicating the seriousness of prediabetes as a risk factor. However, a 2009 survey showed that only 59% of patients who had been told they had prediabetes felt they were at risk for diabetes.[5] When clinicians talk to patients about prediabetes, they should present the risk factor within the spectrum of glucose tolerance. Although it is only a risk factor, clinicians should emphasize that prediabetes remains a serious concern that will not lessen without intervention.

Slide 17

Male patient with T2DM

"And… but no one ever said, yes, you are prediabetic, or, yes, you are diabetic, or, no, you're not. It's always, you might be and… I'd leave and… so if you'd ever ask me, I'm like, I don't know. He said I might be. But they've said you might be for so long that I assume that I am."

Notes: Quote included as an example of why clinicians need to be clear in the diagnosis.

Slide 18

DELIVERING THE DIAGNOSIS: STEP ONE

- Explain the diagnosis
  - Name the disease

Notes: These four steps fall outside the appointment template of History, Assessment, and Plan. After a clinician completes the history and assessment, the four steps of delivering the diagnosis would precede creating a plan.

Name the disease – One of the sources of uncertainty for patients is not understanding if the clinician has diagnosed them with prediabetes or type 2 diabetes, what the difference is, and why it matters. We are early on in this research and are still working to figure out how patients with prediabetes think about their disease and how that diagnosis affects their behavior. We do know that patients think prediabetes is less severe than type 2 and they don't think it's chronic or permanent.[6]

Stick with words that you and the patient can understand and agree on. If you determine that a patient should be told that he has prediabetes, don't confuse the patient with impaired fasting glucose or impaired glucose tolerance.

Slide 19

DELIVERING THE DIAGNOSIS: STEP ONE

- Explain the diagnosis
  - Name the disease
  - Pause
  - Briefly outline your medical decision making for the diagnosis, using patient-centered language (avoid medical jargon)
- Check for understanding
  - *What do you know about diabetes?*

Notes: Pause – Giving a diagnosis is only the initiation of a dialogue. This clinical encounter should be more about listening to the patient and collecting information for shared decision making.

Slide 20

Male patient with T2DM

"I mean I know… I know it's a blood sugar… like not eating healthy or whatever. But very little information about it from the doctor. It didn't seem like… coming from him, it didn't seem like a real big deal… yeah, it was just like a cold, here's a prescription."

Notes: Example quote for how patients report that clinician behavior can reduce patient perception of severity.

Slide 21

DELIVERING THE DIAGNOSIS: STEP TWO

- Explore patient perspectives: checking for shared meaning
    - Susceptibility
        - *Have you ever thought that you could get diabetes? Why or why not?*
        - *Why do you think it started when it did?*
    - Severity
        - *What do you fear most about diabetes?*
        - Chronic nature
            - *Do you expect diabetes to have a long or a short course?*

Notes: Shared meaning – looking for differences in patient and clinician perceptions

Severity and susceptibility are culturally-bound.[3] Don't make assumptions about these two concepts. Patients will have different perceptions of each based on their family history, social experience, and where they live.

Slide 22

Male patient diagnosed with T2DM

"I'd like [providers] to know that there's an emotional factor that comes along with stress. Not stress, but, you know, any kind of diagnosis, no matter what it is. It could be the simplest thing. I mean diabetes was just… it's not as bad as cancer, but you still have it. You see what I'm saying? Um… no matter what, it's just very emotional."

Notes: Quote set up as a transition to the next idea of what can be accomplished after telling the patient the diagnosis.

Slide 23

DELIVERING THE DIAGNOSIS: STEP THREE

- Establish the goal for the appointment
    - Chronic diseases are different than acute conditions. You can't cure the patient in one day. What you do today is the beginning of a long-term treatment plan.
        - What does the patient want to accomplish today?
    - Is the patient emotionally prepared to talk about treatment options?

Notes: After telling the patient the news, it is important to take the time to discuss the patient's goals for the encounter.

Know that telling a patient about this diagnosis can be psychologically disconcerting. The patient may not be able to have a conversation about treatment options. Before you start listing off what's going to happen next, take an assessment of what the patient is ready to do in this appointment.

Slide 24

Female patient diagnosed with T2DM

"I was hurt, I was scared, I was depressed, I worried… all of those things because what… like I said, when they first told me, it was, your A1C is up… is at a level high, and I think what we need to do is to put you on medicine. But I was missing a lot of the information about, okay, what is exactly happening to me right now? And again, he didn't say… and I didn't know what to ask. Like I said, I heard of diabetes, and I know that it could be serious, and that's really all I know. So the fact that I did not have the information really sent me in a…because when I left out of his office, I went in my car and I sat there and I just cried because I was like… literally I'm scared to death."

Notes: Quote further illustrates the potential state of mind of a patient after hearing the news of diagnosis.

Slide 25

DELIVERING THE DIAGNOSIS: STEP FOUR

- Elicit patient preferences
    - Assess lifestyle behavior change facilitators and barriers.
    - Include the patient in decision making about treatment options
        - *What kind of treatment do you think you should receive?*
        - *What are the most important results you hope to receive from this treatment?*

Notes: Assess facilitators and barriers.

How do they feel about medications? What is their first response to diet and exercise changes?

Explore patient experience and social support

How does the patient think their family and friends will support them in changes?

Some people are more capable of overcoming challenges in their lives, and a disease diagnosis is simply another challenge for them to overcome. Although this isn't the type of question that you can generally ask in a first meeting encounter, it is the type of trait that you can identify with your own patient panel. Don't overlook the power of personal determination and "stick-to-it-iveness" when having these discussions with your patients.

Slide 26

[Potential transition] "This is only the beginning of the conversation. This isn't the type of encounter that is going to have a nice bow tied around it. It is simply the beginning of the conversation."

How do you close this encounter?

- This is only the beginning of the conversation
- Closing the appointment should focus on…
    - Setting expectations
    - Previewing educational options
    - Creating a collaborative relationship

Notes: Close of encounter should focus on…

1. Setting expectations: Help the patient understand that this is a journey. Be realistic in sharing that it will be difficult, but it's important and worth the effort.
2. Previewing educational options: Most patients need information but they need it at a level they can understand which isn't likely available within the time you have in this first appointment. Be ready with resources to share.
3. Creating relationship: Remember that this is not only a relationship with you, but with the clinic, and with the system. Diagnosis of a chronic disease can mean a new level of integration into the healthcare system through referrals and numerous appointments. It is critical that you create a relationship that encourages and facilitates the patient's engagement in care. They need to want to come back because they need to come back.

Focusing the close on these three actions means you might not make any decisions with the patient in this appointment, you may not write a prescription, and you may not order any tests.

CLINICAL DOCUMENTATION LECTURE

Slide 27

[Potential transition] "Now that you've had the opportunity to practice how to have the diagnosis discussion, let's talk about how you document it."

Documenting the encounter

Notes:

Slide 28

[Potential transition] "Coding this type of encounter is important for creating the history of present illness in a way that is easily searchable and understandable by the healthcare team."

ICD coding

- T2DM ICD-10 Codes: E11.0 – E11.9
- Prediabetes ICD-10 Code: R73.03

Notes: Include information here that may be unique to your healthcare system's health records management system. Is there a best place to enter a diagnosis so that it is tagged in future encounters? Is putting it in the problem list enough?

Slide 29

[Potential transition] "Remember the diagnosis is simply the beginning of the conversation. Before small groups, we talked about how diagnosis of a chronic disease indicates a new level of integration into the healthcare system through referrals and numerous appointments. A diagnosis can only be truly meaningful if it is shared among you, the patient, and the healthcare team. The clinical note is your voice in advocating for the patient, to the healthcare team, and to the system."

Documenting the diagnosis

- Shared meaningful diagnosis is shared among …
    - You
    - The patient
    - Healthcare team
- Your voice to your team is the clinical note
    - tells a coherent story about the patient from one visit to the next
    - synthesizes patient information
    - facilitates the linkage of one encounter note to related information and encounters
    - coordinates information from different sources
    - facilitates follow-up

Notes:

Slide 30

[Potential transition] "Here on the left we have characteristics that you are likely familiar with. Notes should be concise – to the point; sufficient – give enough information; relevant – pertaining to the primary complaint; current – information should reflect what is happening at the time of encounter; and accurate – valid reflection of what's happening."

"On the right are characteristics of a quality note that require more advanced medical decision making and communication skills. Notes should be clear and understandable for the entire healthcare team – this is how we coordinate care in many ways. We rely on the notes of previous providers to know what has occurred in the past and why decisions were made. Increasingly, notes need to be clear and understandable to patients. As 'open notes' become more common and patients become more engaged in their own care, the health record can help them understand decisions that have been made. One strategy that can build patient alliances is to pull alongside a patient with their health record and review what is in his or her record."

What is in a quality note?

- Concise
- Sufficient
- Relevant
- Current
- Accurate

- Clear and understandable to…
  - patients
  - subsequent providers
- Explains your thought process
- Explains plan of care
- Prioritized for action
- Few or no abbreviations, and understandable syntax

Notes: The note should explain the thought process and the plan of care. It should also prioritize items for action. Don't fall into the trap of overusing abbreviations or syntax. Although "TLC" may mean one thing in the course of diabetes prevention and treatment, it means a far different thing outside of that context.

Each of these characteristics describes how a note should be written in a single encounter. But don't overlook the role of each note in telling a continuous story about the patient. We are privileged to contribute to that story in single acts or full arcs, but that patient's story likely began far before you met her and will continue beyond today.

This is a time to address how learners use a "copy-forward" function (we recommend caution) or how learners use auto fill template language such as a click box that will fill in "patient acknowledges plan" (this is meaningless without context).

(Characteristics of a quality note are from Hanson et al[7])

Slide 31

Examples

Prediabetes R73.03: A1C 5.9%. Follow-up in 6 months with repeat A1C.
INSUFFICIENT

Prediabetes R73.03: 72 yo M with prediabetes, A1C 5.9%. Poor diet and no exercise. Discussed medication vs lifestyle changes. Follow-up in 6 months with repeat A1C.
ACCEPTABLE

Prediabetes R73.03: 72 yo M with new diagnosis prediabetes based on A1C 5.9%. Recent history of numerous cruises, poor diet, and absence of exercise. Discussed future AE of DM and need for interventions now to prevent development of DM. Discussed medication vs lifestyle changes. Patient elects for lifestyle change. Will follow-up in 6 months with repeat A1C.
DM: preDM April '19 A1C 5.9% (May '18 5.5%)
BP: WNL today, on ACE inhibitor (Lisinopril)
LAST ASCVD of 21% in April 2019, on Lipitor 20mg
LAST CMP/BMP: SCr 0.8 on 11 April 2019
LAST MICROALBUMIN: 11 April 2019, 47
MEANINGFUL

Notes: These three examples are notes from the same encounter.

The first note demonstrates an insufficient amount of information to establish continuity of care.

The second note demonstrates an acceptable amount of information about the encounter but does not create a shared meaningful diagnosis with the next clinician.

The third note adds this meaning – it recounts the progression of disease, it shows how the clinician and patient made decisions together, and it provides enough information for the next clinician to understand the medical decision making.

Slide 32

What to include in S/O

- STEP TWO: Explore patient perspectives: checking for shared meaning
  - "This is a wake-up call."
  - "Husband likes to cook fried foods."
  - "Thinks family hx caused this but understands diet component."
  - (Patient with preDM) "She had a neighbor growing up who lost a foot due to DM and she is scared she actually has DM. She thinks the labs may be inaccurate and would like to redraw them."
  - "Decrease in exercise due to mother and sister passing."
  - "Patient is recently retired from military, now truck driver, has noted ~30 lb weight gain in this time. More sedentary lifestyle."

Notes: We've pulled some examples of how to comment on these steps within the S/O portion of the note.

Slide 33

[Potential transition] "What about the A/P? Did the patient provide information that impacted your choices today? If you were seeing this patient at a subsequent visit, is this information you would want to have when considering changing treatment?"

What to include in A/P

- STEP FOUR: Elicit patient preferences
- If it's relevant to long-term management and continuity, consider…
  - "Husband could benefit too. Pt believes DZ is manageable."
  - "Patient is open to lifestyle modifications before trying meds. Patient does not have exercise routine, but is open to trying small incremental activity changes to achieve 30 mins x 5 days per week. She is OK with weekly weigh-ins at home to track progress."
  - "If no change at follow up will consider medication – metformin."

Notes: We've pulled some examples of how to comment on these steps within the A/P portion of the note.

Slide 34

Looking forward

- Clinical practice application and Clinical documentation application
- Learner knowledge check
- Curriculum evaluation

Notes: Learners will likely be anticipating an end to the intervention. Before they leave, talk about three different items and collect two.

CLINICAL PRACTICE APPLICATION and CLINICAL DOCUMENTATION APPLICATION (pp. 28-29) are provided for them to use in precepting their next clinical appointment when they can apply these new skills. Make sure preceptors have copies of these sheets in their office, but it's valuable to give learners a copy here to preview what they will see later.

LEARNER KNOWLEDGE CHECK (p. 41) is a short assessment of what they learned for the day. Ask them to complete these and mark them with their names so that you can see who met the learning objectives.

CURRICULUM EVALUATION (p. 44) is for you as a teacher to see how effective the intervention was. This is not an evaluation of learners.

References

1. Standards of Medical Care in Diabetes-2019 Abridged for Primary Care Providers. *Clinical diabetes : a publication of the American Diabetes Association.* 2019;37(1):11-34.

2. Bloom Cerkoney KA, Hart LK. The Relationship Between the Health Belief Model and Compliance of Persons with Diabetes Mellitus. *Diabetes Care.* 1980;3(5):594-598.

3. Ledford CJW, Seehusen DA, Crawford PF. Geographic and Race/Ethnicity Differences in Patient Perceptions of Diabetes. *Journal of Primary Care & Community Health.* 2019;10:2150132719845819.

4. Seehusen DA, Fisher CL, Rider HA, et al. Exploring patient perspectives of prediabetes and diabetes severity: a qualitative study. *Psychology & health.* 2019:1-14.

5. Gallivan J, Brown C, Greenberg R, Clark CM. Predictors of perceived risk of the development of diabetes. *Diabetes Spectrum.* 2009;22(3):163-169.

6. Ledford CJW, Seehusen DA, Crawford PF. The relationship between patient perceptions of diabetes and glycemic control: A study of patients living with prediabetes or type 2 diabetes. *Patient education and counseling.* 2019.

7. Hanson JL, Stephens MB, Pangaro LN, Gimbel RW. Quality of outpatient clinical notes: a stakeholder definition derived through qualitative research. *BMC health services research.* 2012;12:407.

ROADMAP

CLINICAL PRACTICE APPLICATION

Step	Notes
**Explain the diagnosis**: Name the condition, clearly explain what it is, and check for understanding.	
**Explore patient perceptions**: Check for shared meaning, including severity and susceptibility.	
**Establish goal for today's appointment**: Knowing diabetes is a chronic condition, determine what you and the patient can accomplish today.	
**Elicit patient preferences**: Include the patient in decision making about treatment options and assessing behavior change facilitators and barriers.	

ROADMAP

CLINICAL DOCUMENTATION APPLICATION

OVERALL CHARACTERISTICS CHECKLIST

- O Does the note explain your thought process?
- O Does the note explain the plan of care?
- O Is the note prioritized for action?
- O Is your note clear and understandable to subsequent providers?
- O Does the note tell a continuous story about the patient?

DIAGNOSIS SPECIFIC DOCUMENTATION

What did you include in the S/O to document patient perspectives, including severity and susceptibility and shared meaning?

What did you include in the A/P to document patient preferences, including the patient in decision making about treatment options and assessing behavior change facilitators and barriers?
