# Supplementary material for: Diabetes ROADMAP: Teaching Guideline Use, Communication, and Documentation When Delivering the Diagnosis of Diabetes
Source: MedEdPORTAL. 2020 Sep 11;16:10959. doi: 10.15766/mep_2374-8265.10959 (PMC7485911; doi:10.15766/mep_2374-8265.10959)
Supplement: Supplementary file 1 — Curriculum Overview.pdfTeaching Guide.pdfROADMAP Presentation.pptxFacilitator Guide.pdfSimulation Resources.pdfAssessment Tools.pdf [file mep_2374-8265.10959-s001.zip › C. ROADMAP Presentation.pptx]

## Slide 1
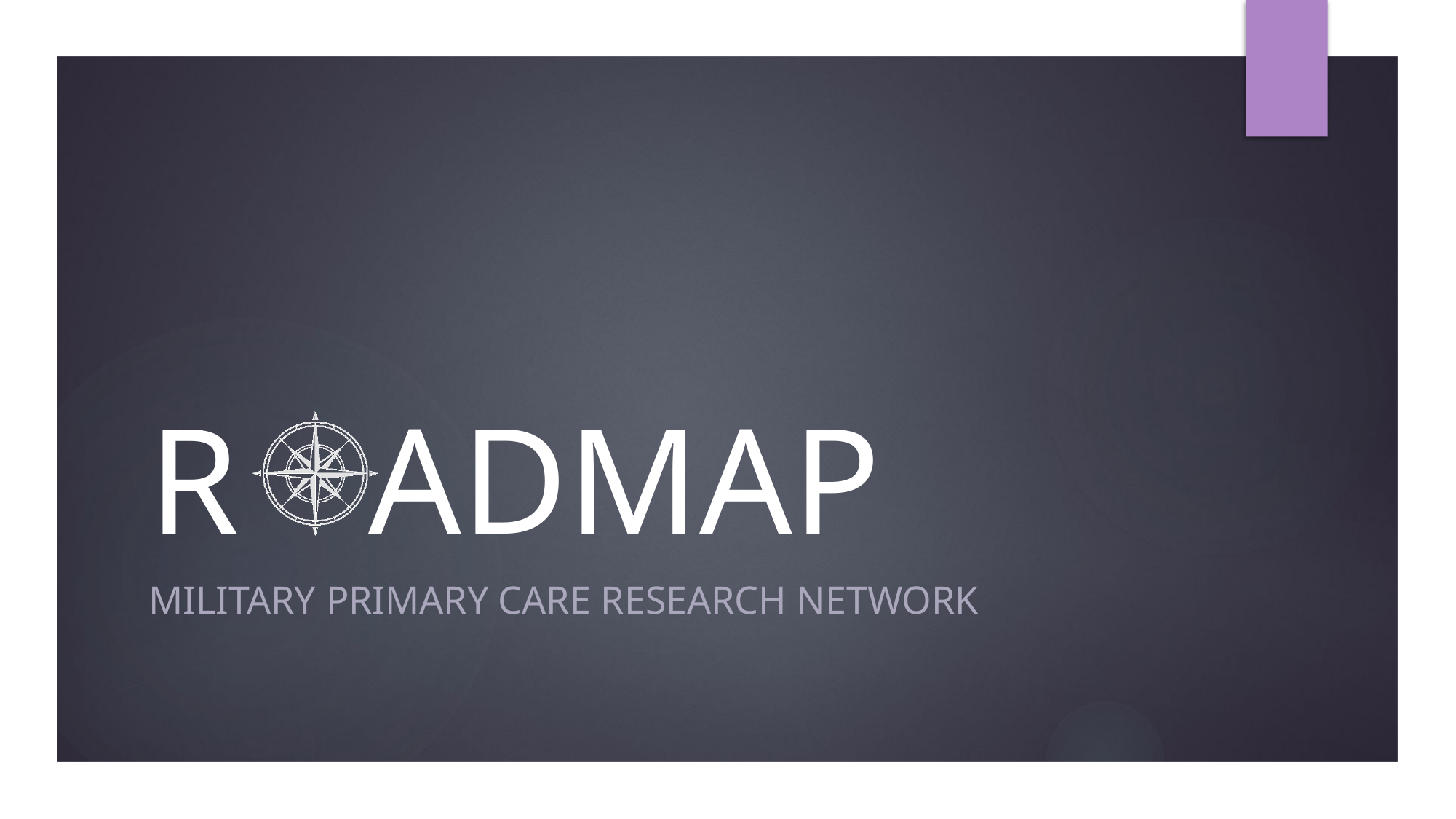

R
ADMAP
Military Primary Care Research Network

## Slide 2
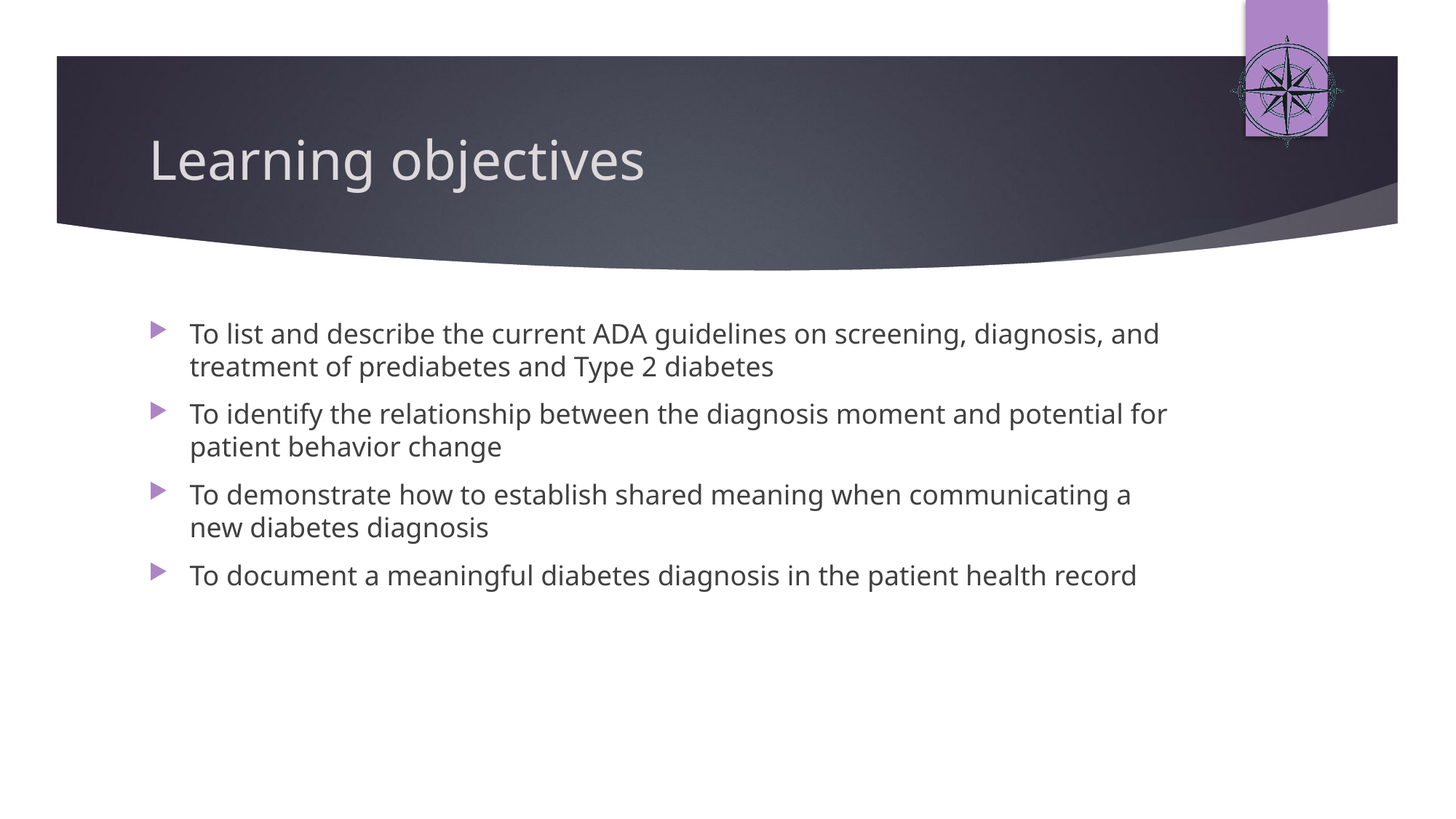

# Learning objectives
To list and describe the current ADA guidelines on screening, diagnosis, and treatment of prediabetes and Type 2 diabetes
To identify the relationship between the diagnosis moment and potential for patient behavior change
To demonstrate how to establish shared meaning when communicating a new diabetes diagnosis
To document a meaningful diabetes diagnosis in the patient health record

## Slide 3
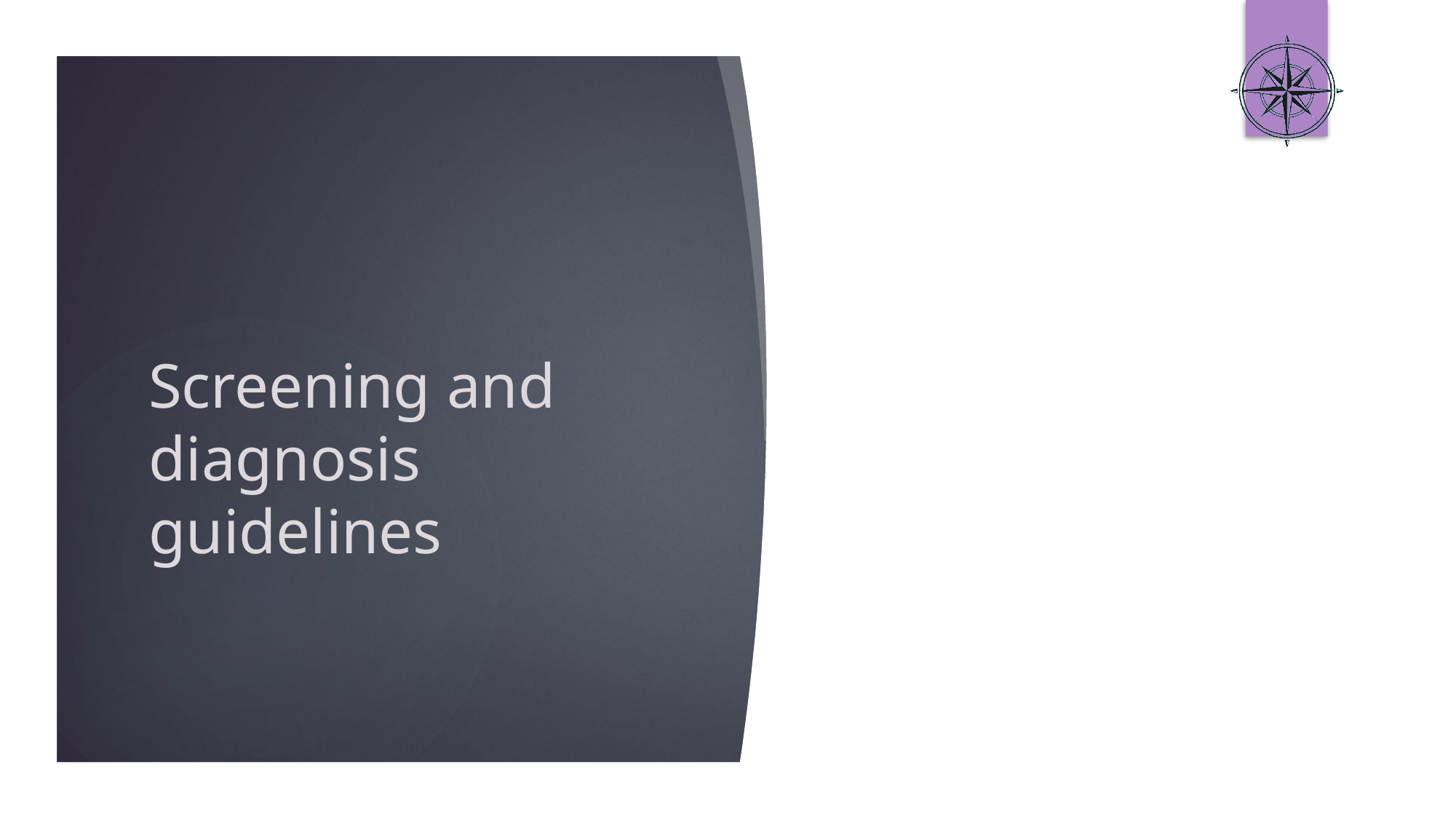

# Screening and diagnosis guidelines

## Slide 4
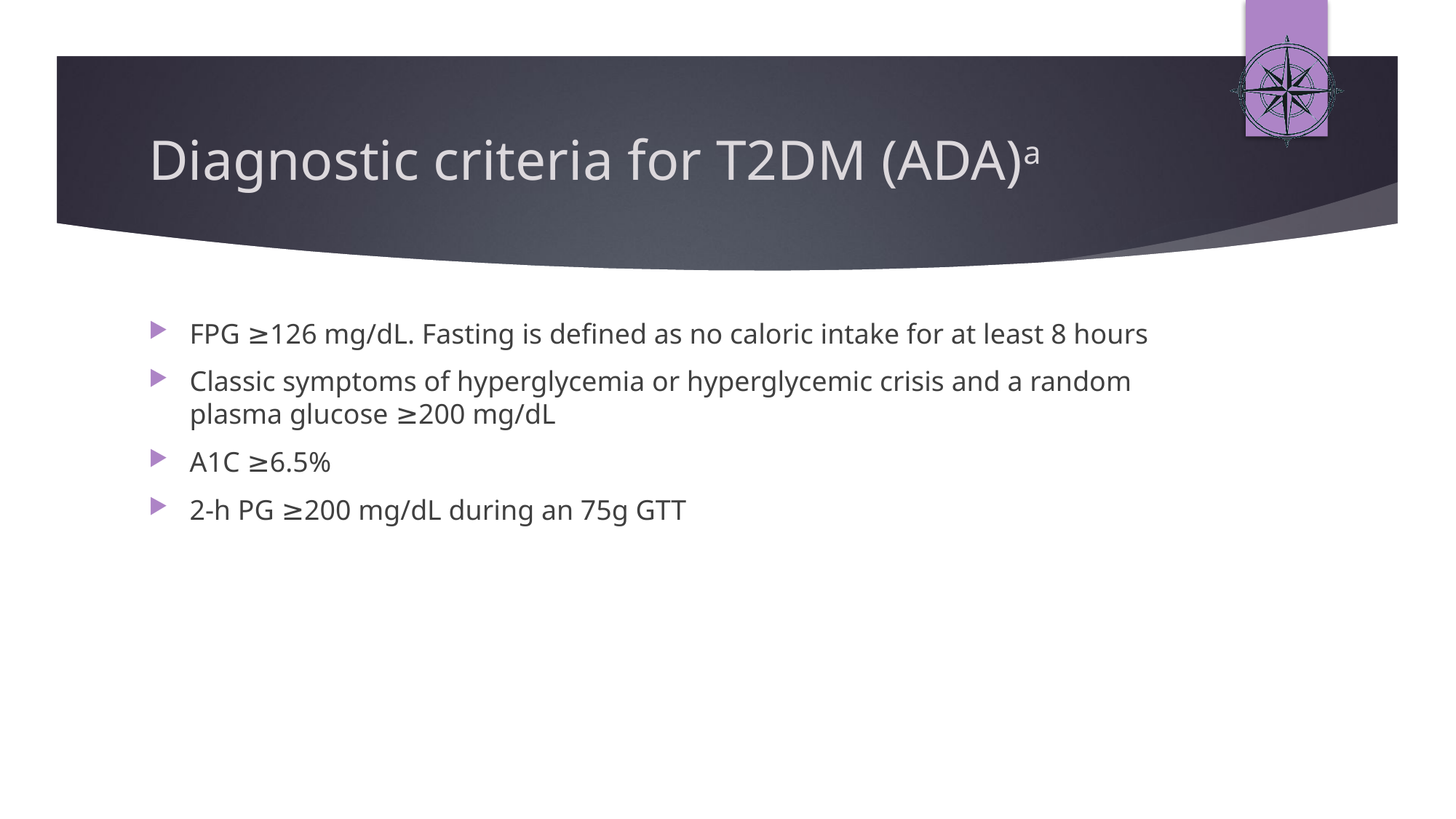

# Diagnostic criteria for T2DM (ADA)a
FPG ≥126 mg/dL. Fasting is defined as no caloric intake for at least 8 hours
Classic symptoms of hyperglycemia or hyperglycemic crisis and a random plasma glucose ≥200 mg/dL
A1C ≥6.5%
2-h PG ≥200 mg/dL during an 75g GTT

## Slide 5
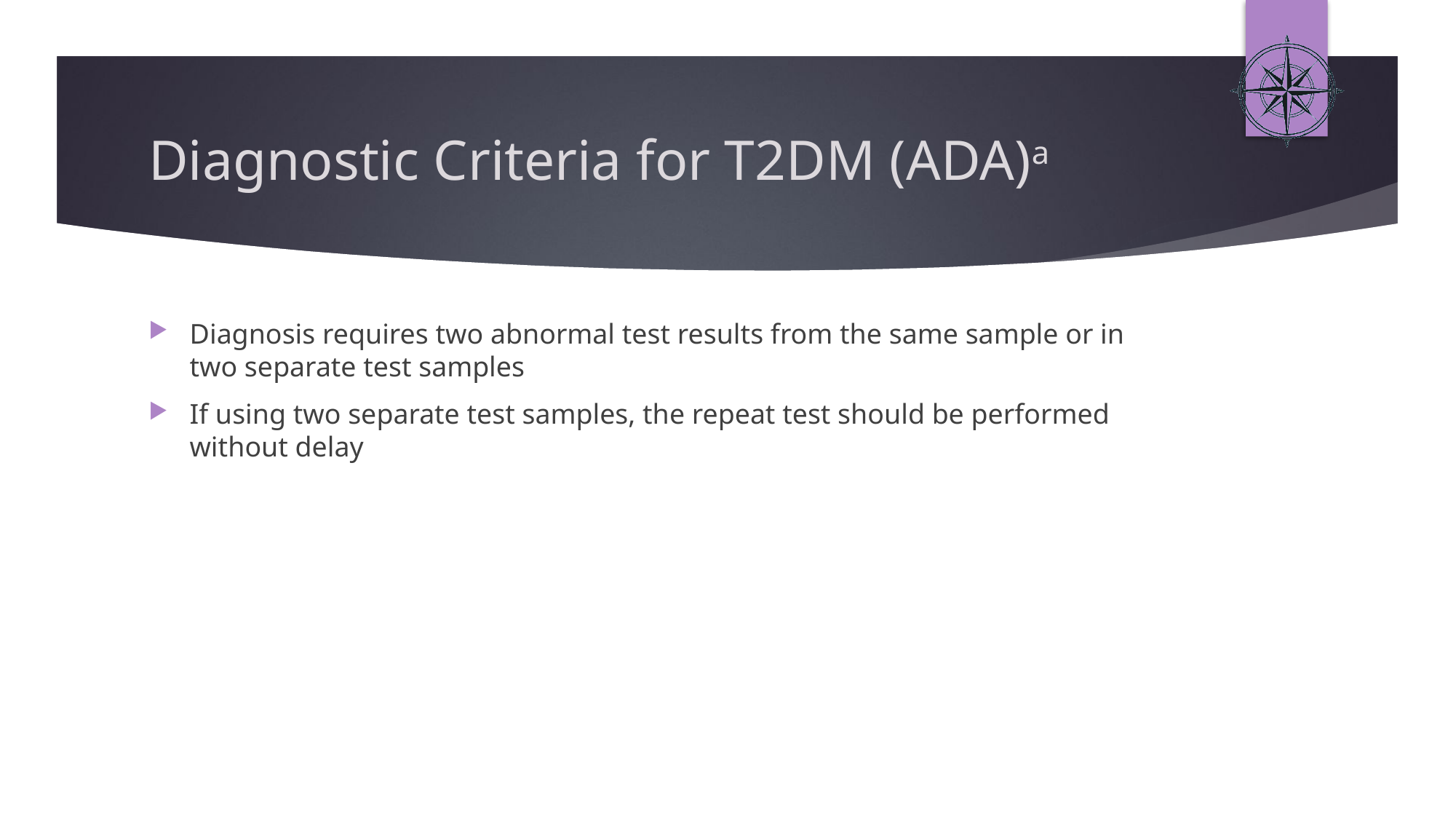

# Diagnostic Criteria for T2DM (ADA)a
Diagnosis requires two abnormal test results from the same sample or in two separate test samples
If using two separate test samples, the repeat test should be performed without delay

## Slide 6
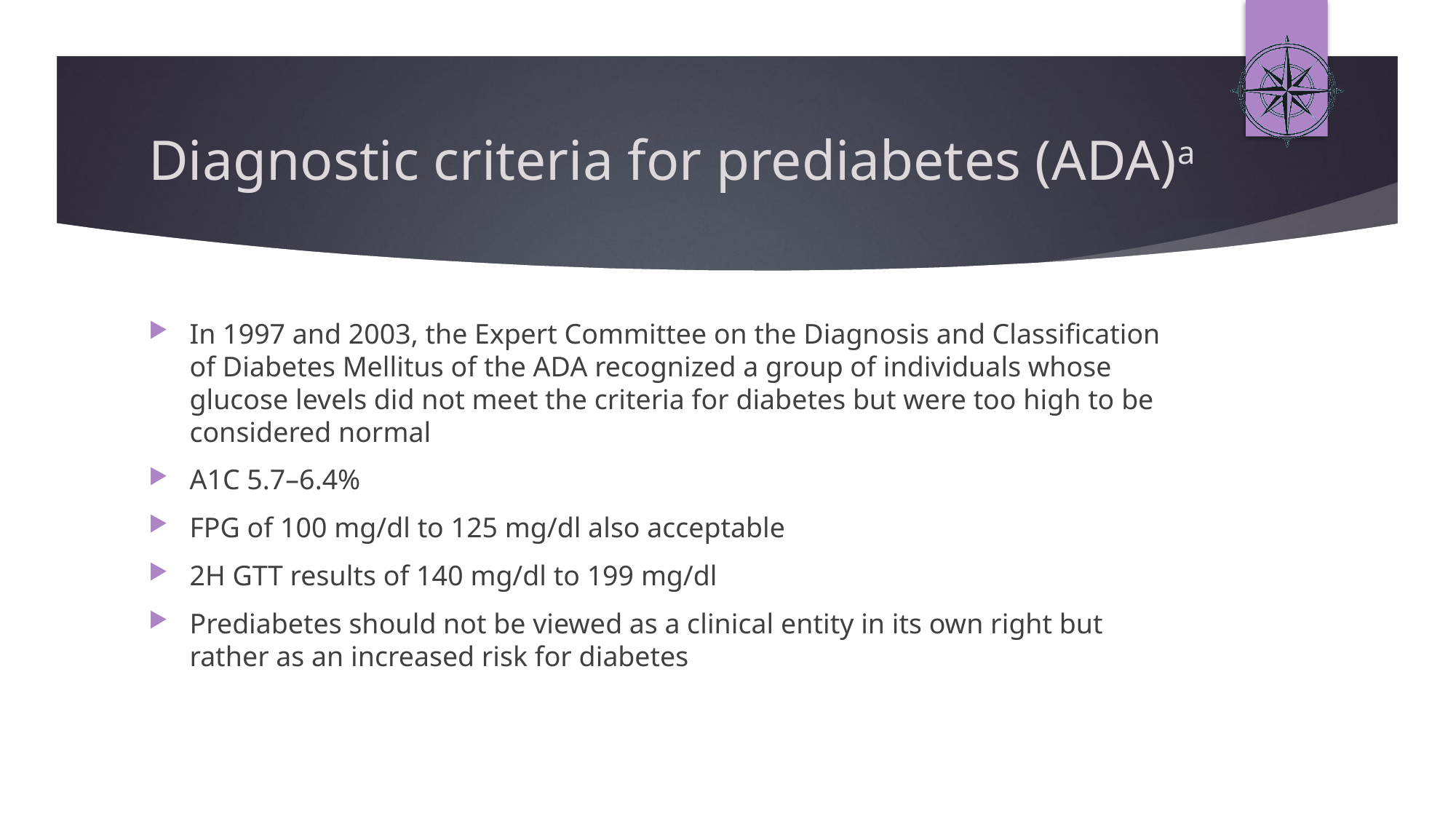

# Diagnostic criteria for prediabetes (ADA)a
In 1997 and 2003, the Expert Committee on the Diagnosis and Classification of Diabetes Mellitus of the ADA recognized a group of individuals whose glucose levels did not meet the criteria for diabetes but were too high to be considered normal
A1C 5.7–6.4%
FPG of 100 mg/dl to 125 mg/dl also acceptable
2H GTT results of 140 mg/dl to 199 mg/dl
Prediabetes should not be viewed as a clinical entity in its own right but rather as an increased risk for diabetes

## Slide 7
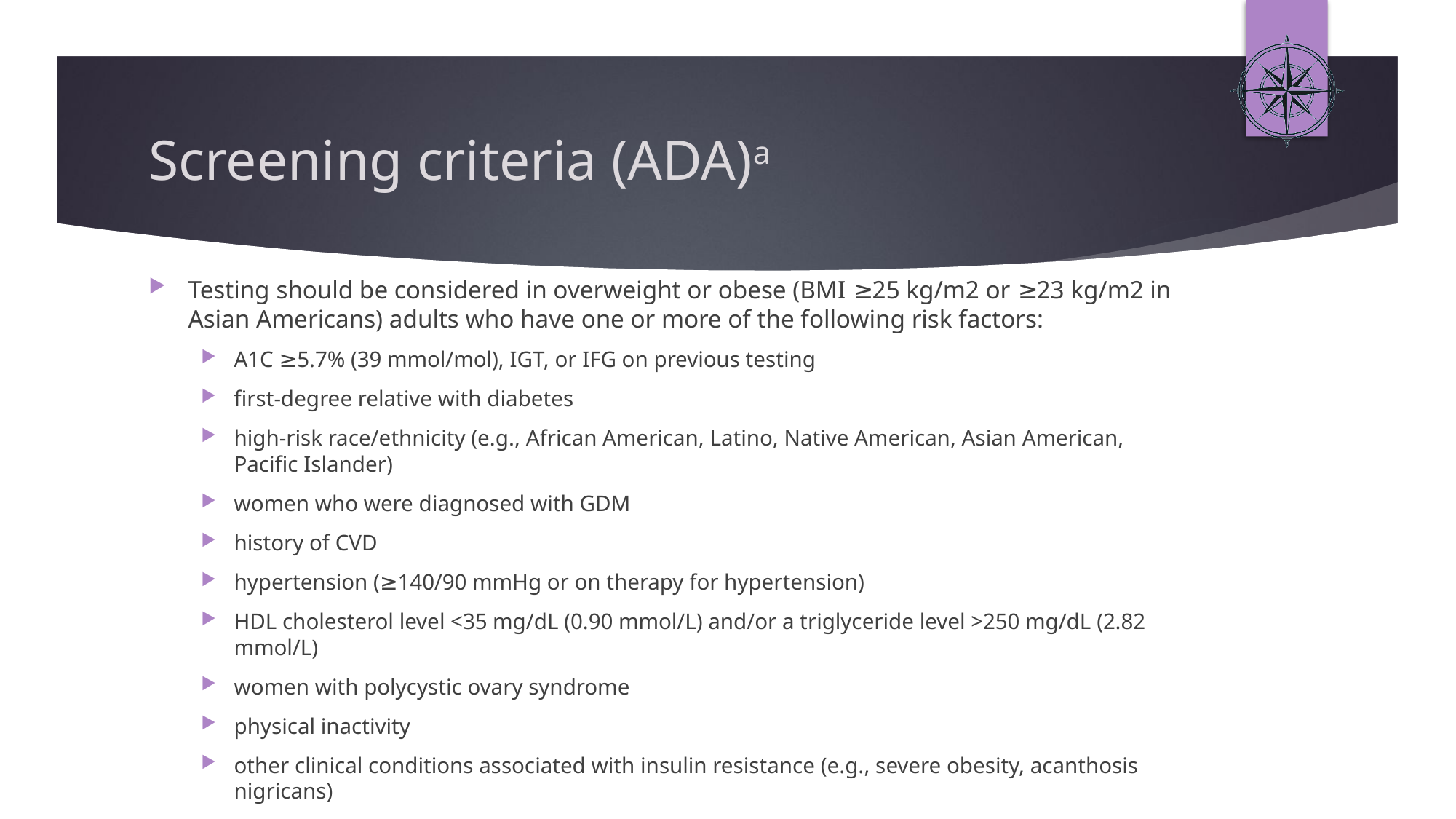

# Screening criteria (ADA)a
Testing should be considered in overweight or obese (BMI ≥25 kg/m2 or ≥23 kg/m2 in Asian Americans) adults who have one or more of the following risk factors:
A1C ≥5.7% (39 mmol/mol), IGT, or IFG on previous testing
first-degree relative with diabetes
high-risk race/ethnicity (e.g., African American, Latino, Native American, Asian American, Pacific Islander)
women who were diagnosed with GDM
history of CVD
hypertension (≥140/90 mmHg or on therapy for hypertension)
HDL cholesterol level <35 mg/dL (0.90 mmol/L) and/or a triglyceride level >250 mg/dL (2.82 mmol/L)
women with polycystic ovary syndrome
physical inactivity
other clinical conditions associated with insulin resistance (e.g., severe obesity, acanthosis nigricans)

## Slide 8
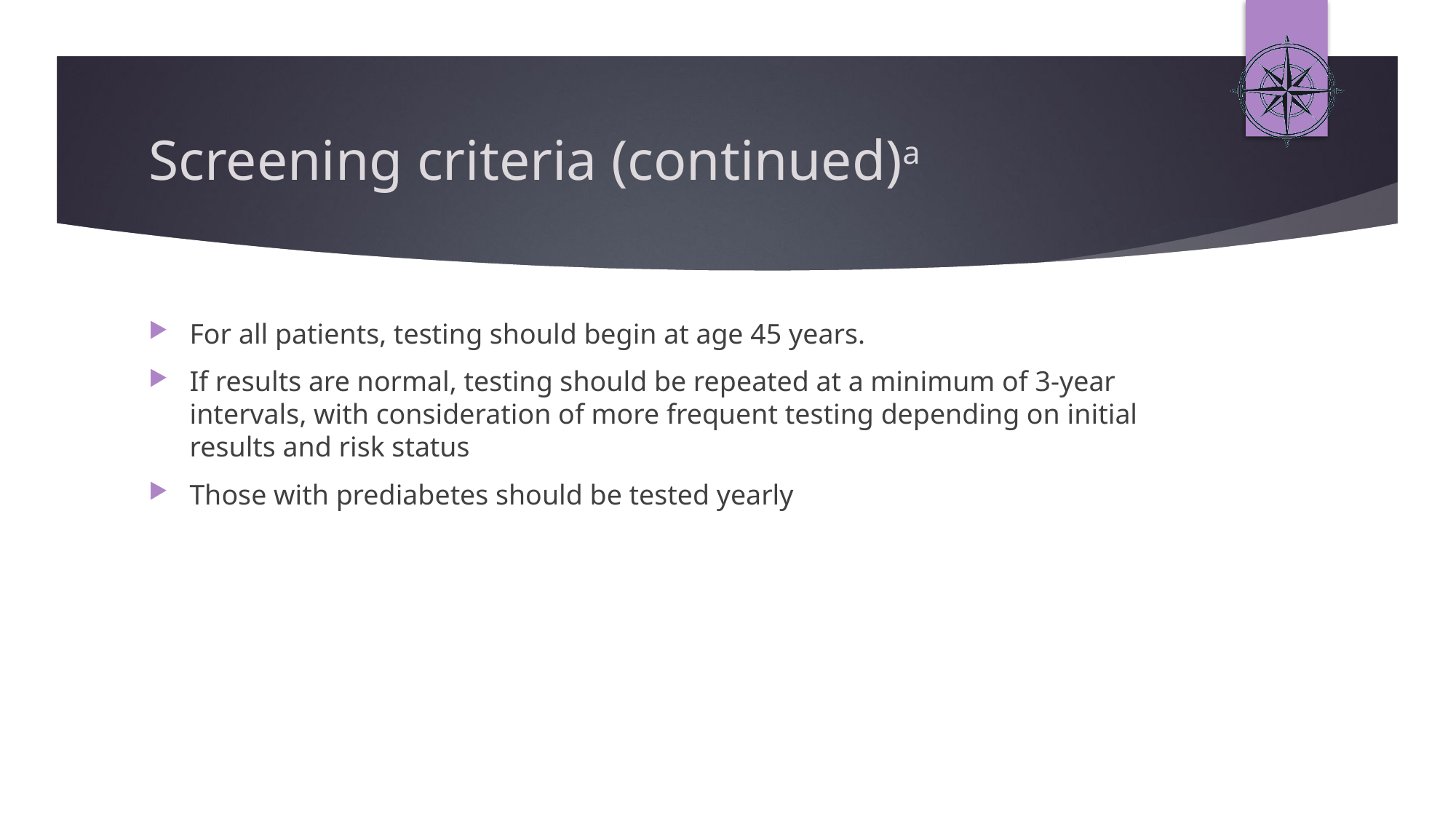

# Screening criteria (continued)a
For all patients, testing should begin at age 45 years.
If results are normal, testing should be repeated at a minimum of 3-year intervals, with consideration of more frequent testing depending on initial results and risk status
Those with prediabetes should be tested yearly

## Slide 9
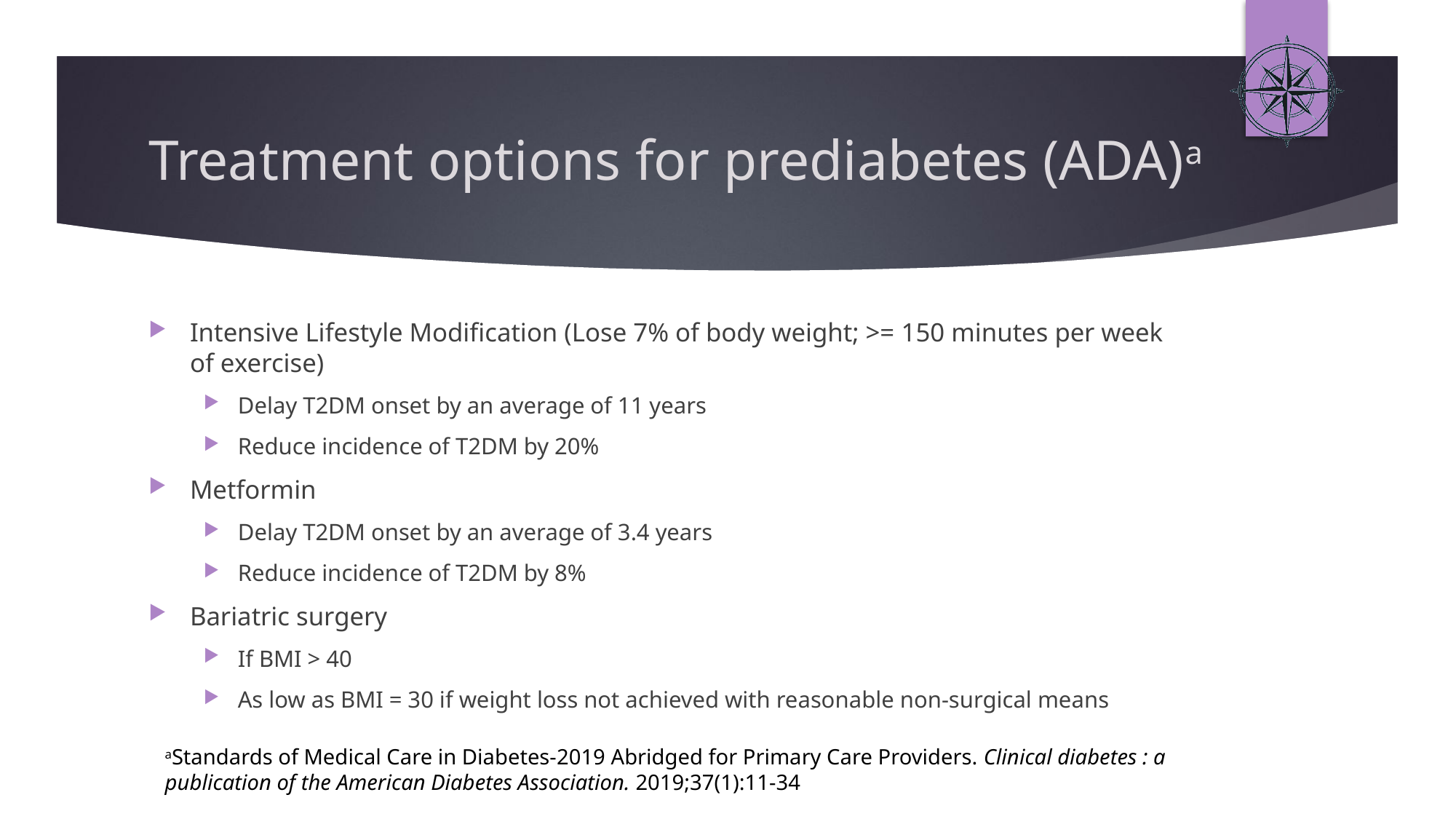

# Treatment options for prediabetes (ADA)a
Intensive Lifestyle Modification (Lose 7% of body weight; >= 150 minutes per week of exercise)
Delay T2DM onset by an average of 11 years
Reduce incidence of T2DM by 20%
Metformin
Delay T2DM onset by an average of 3.4 years
Reduce incidence of T2DM by 8%
Bariatric surgery
If BMI > 40
As low as BMI = 30 if weight loss not achieved with reasonable non-surgical means
aStandards of Medical Care in Diabetes-2019 Abridged for Primary Care Providers. Clinical diabetes : a publication of the American Diabetes Association. 2019;37(1):11-34

## Slide 10
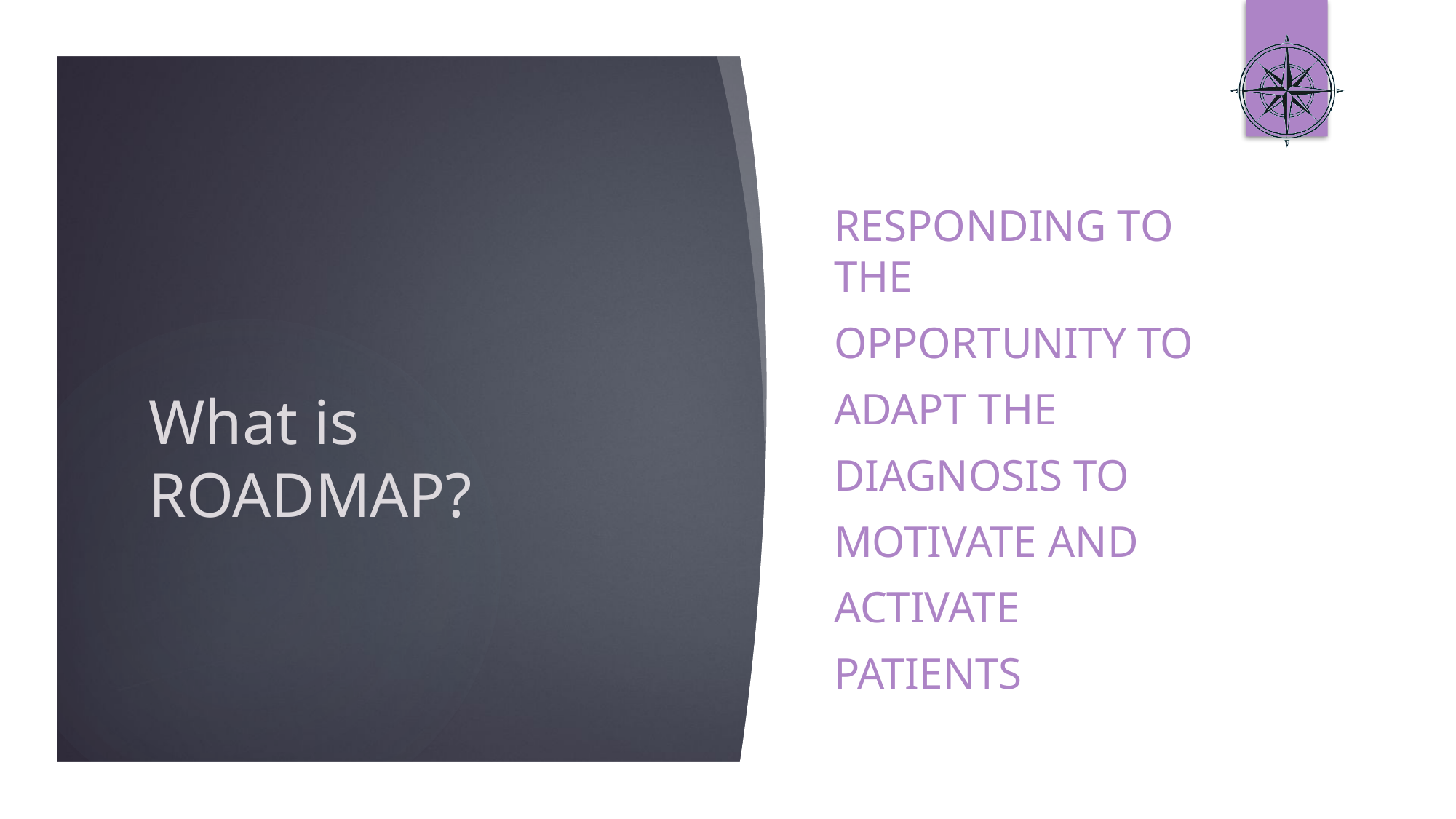

Responding to the
Opportunity to
Adapt the
Diagnosis to
Motivate and
Activate
Patients
# What is ROADMAP?

## Slide 11
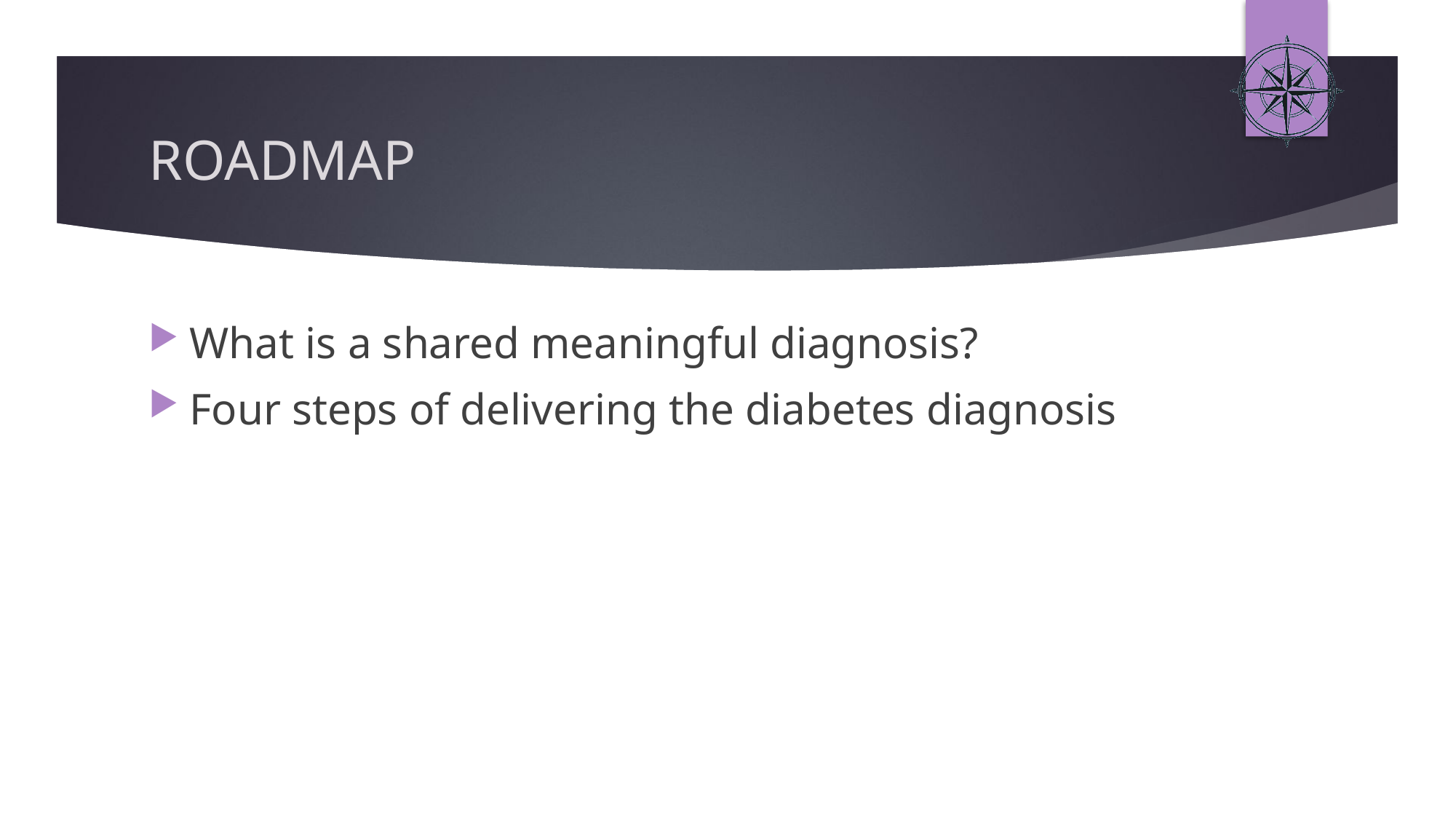

# ROADMAP
What is a shared meaningful diagnosis?
Four steps of delivering the diabetes diagnosis

## Slide 12
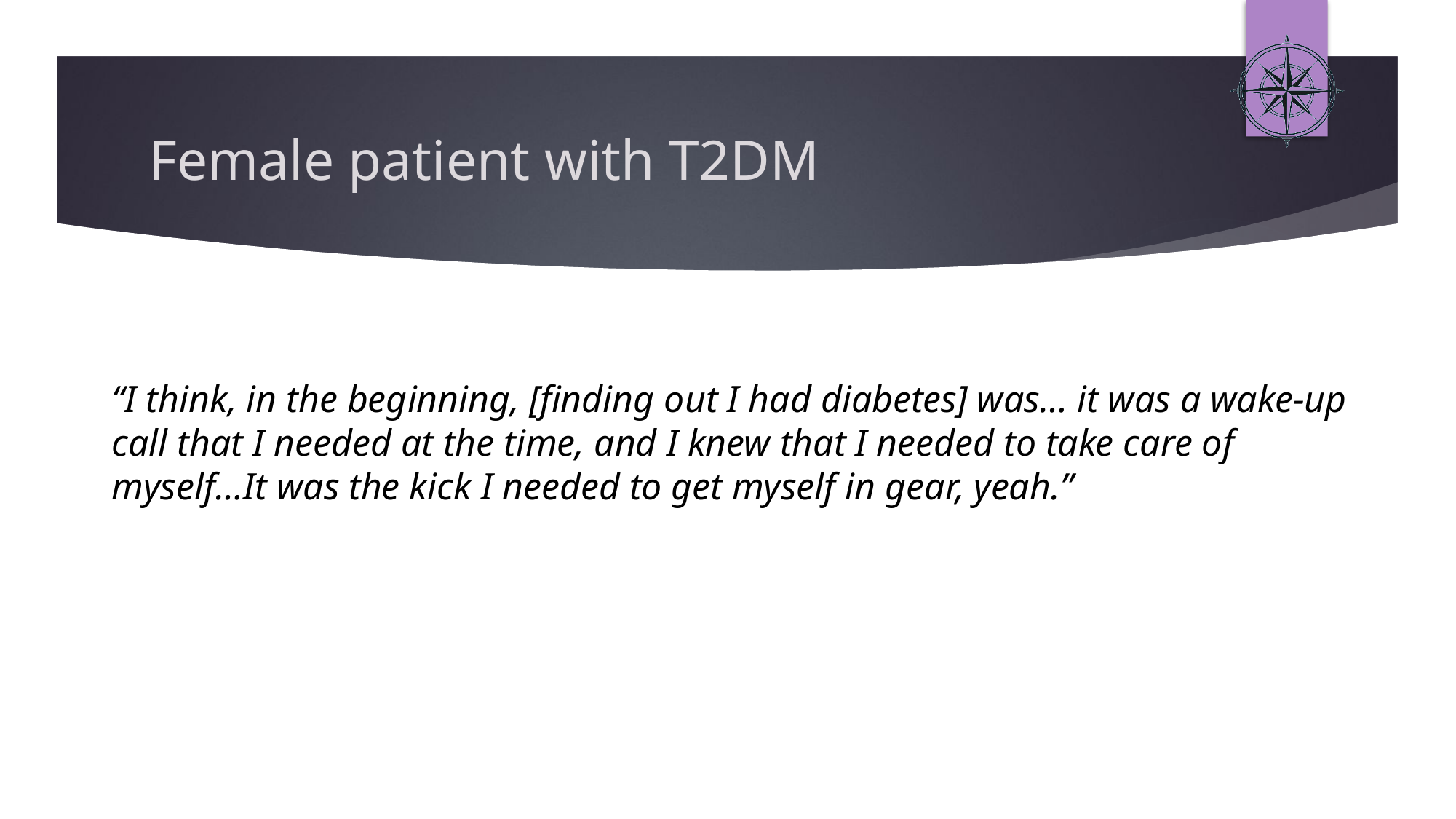

# Female patient with T2DM
“I think, in the beginning, [finding out I had diabetes] was… it was a wake-up call that I needed at the time, and I knew that I needed to take care of myself…It was the kick I needed to get myself in gear, yeah.”

## Slide 13
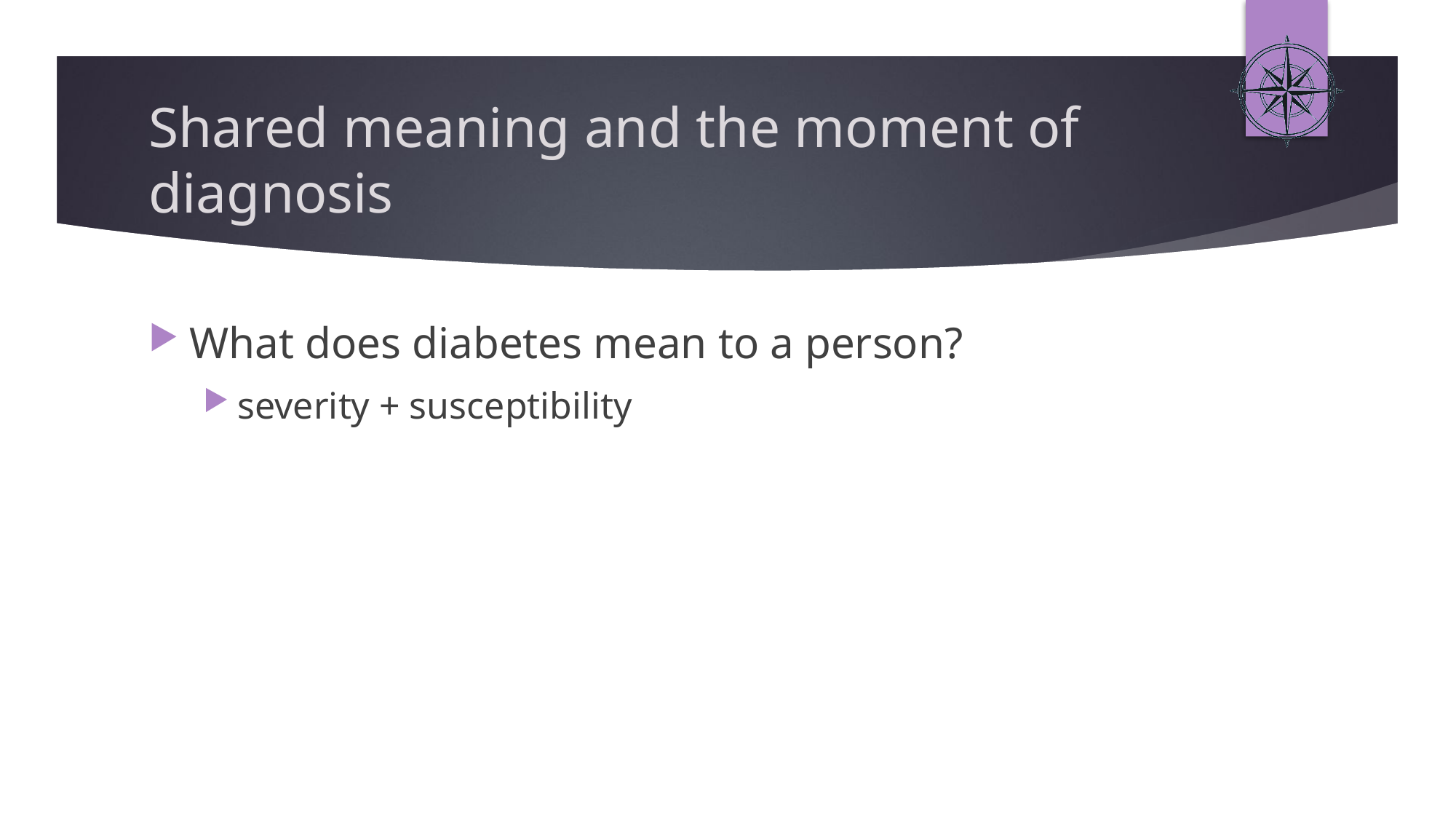

# Shared meaning and the moment of diagnosis
What does diabetes mean to a person?
severity + susceptibility

## Slide 14
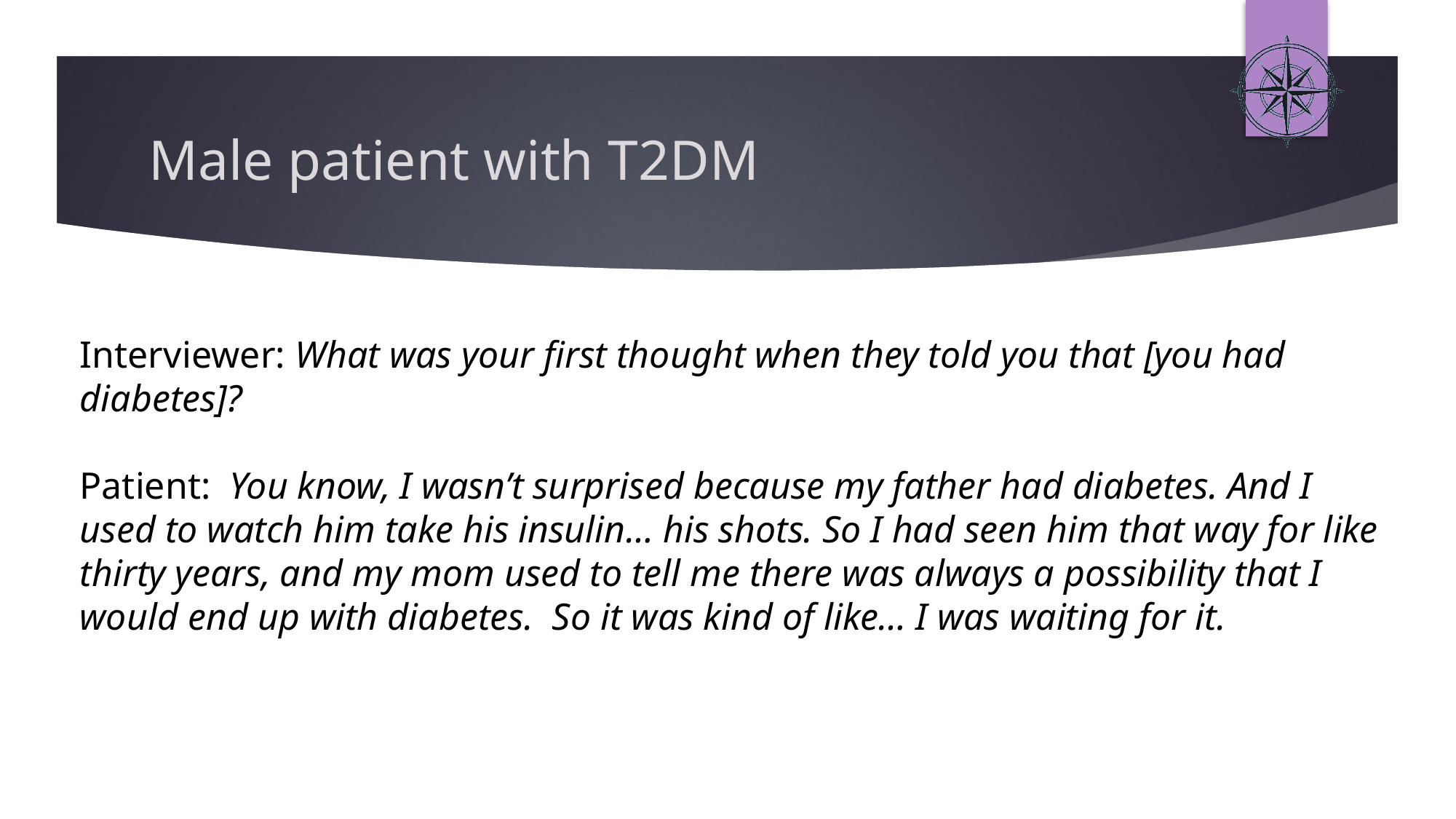

# Male patient with T2DM
Interviewer: What was your first thought when they told you that [you had diabetes]?
Patient: You know, I wasn’t surprised because my father had diabetes. And I used to watch him take his insulin… his shots. So I had seen him that way for like thirty years, and my mom used to tell me there was always a possibility that I would end up with diabetes. So it was kind of like… I was waiting for it.

## Slide 15
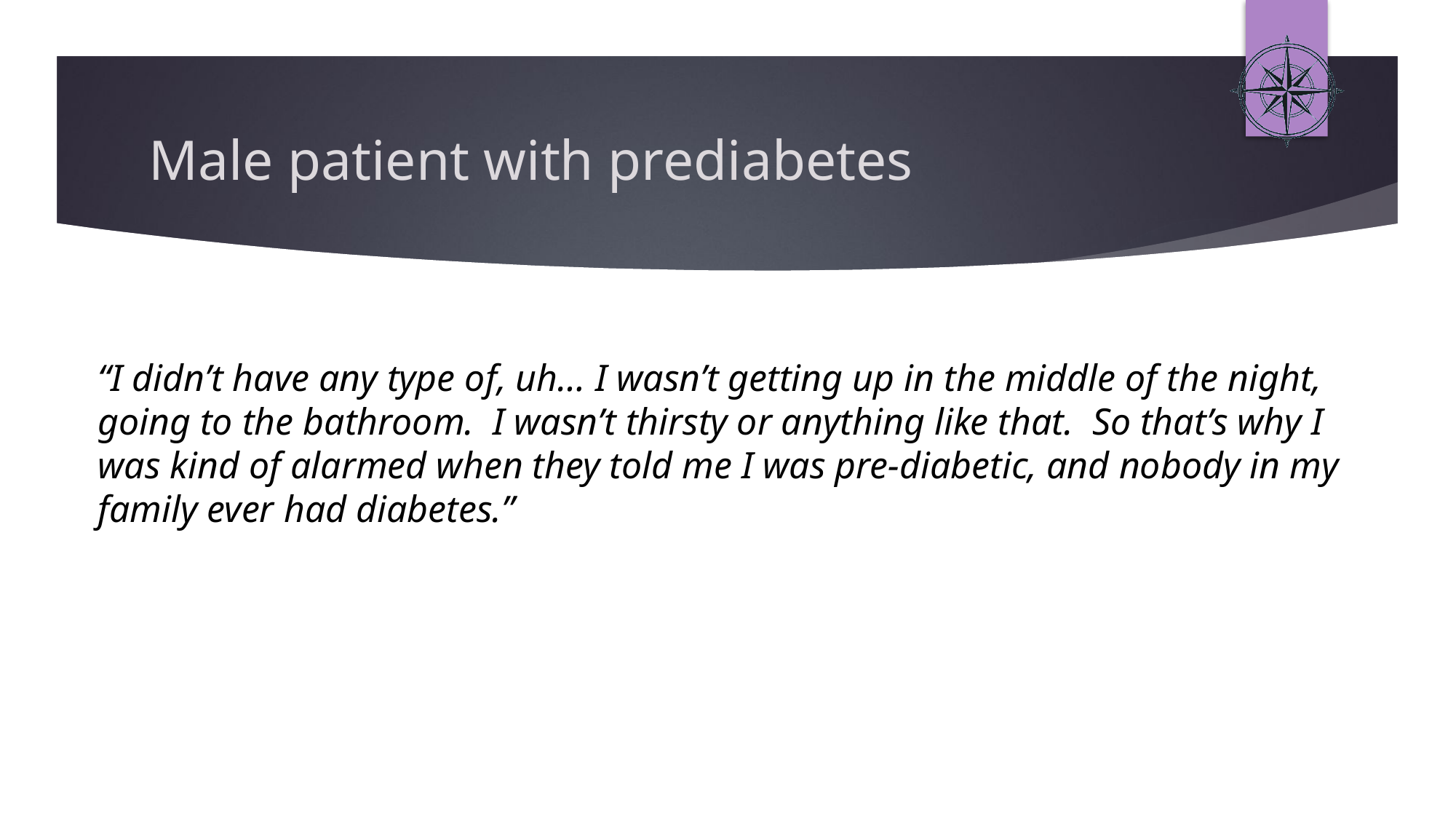

# Male patient with prediabetes
“I didn’t have any type of, uh… I wasn’t getting up in the middle of the night, going to the bathroom. I wasn’t thirsty or anything like that. So that’s why I was kind of alarmed when they told me I was pre-diabetic, and nobody in my family ever had diabetes.”

## Slide 16
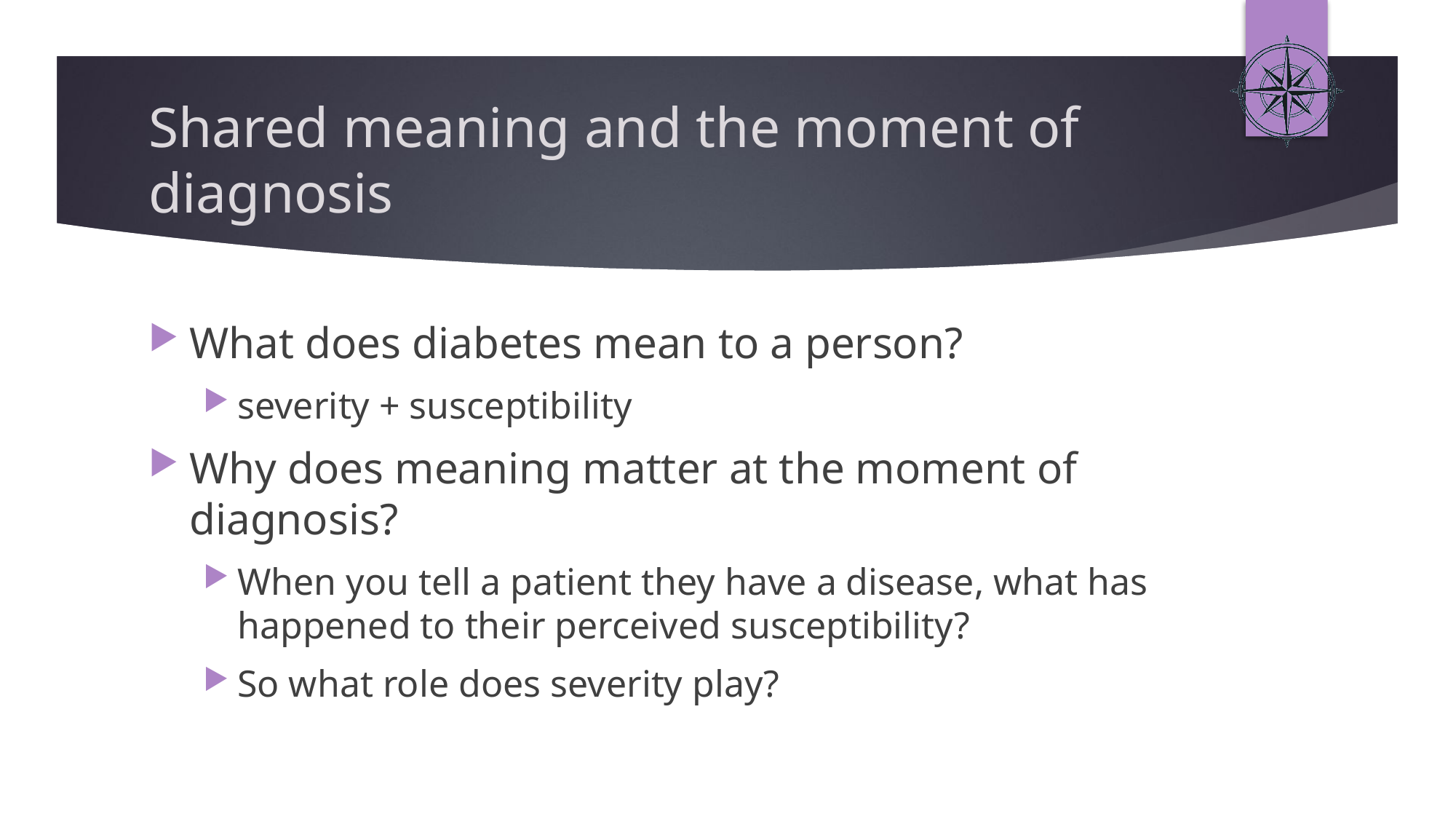

# Shared meaning and the moment of diagnosis
What does diabetes mean to a person?
severity + susceptibility
Why does meaning matter at the moment of diagnosis?
When you tell a patient they have a disease, what has happened to their perceived susceptibility?
So what role does severity play?

## Slide 17
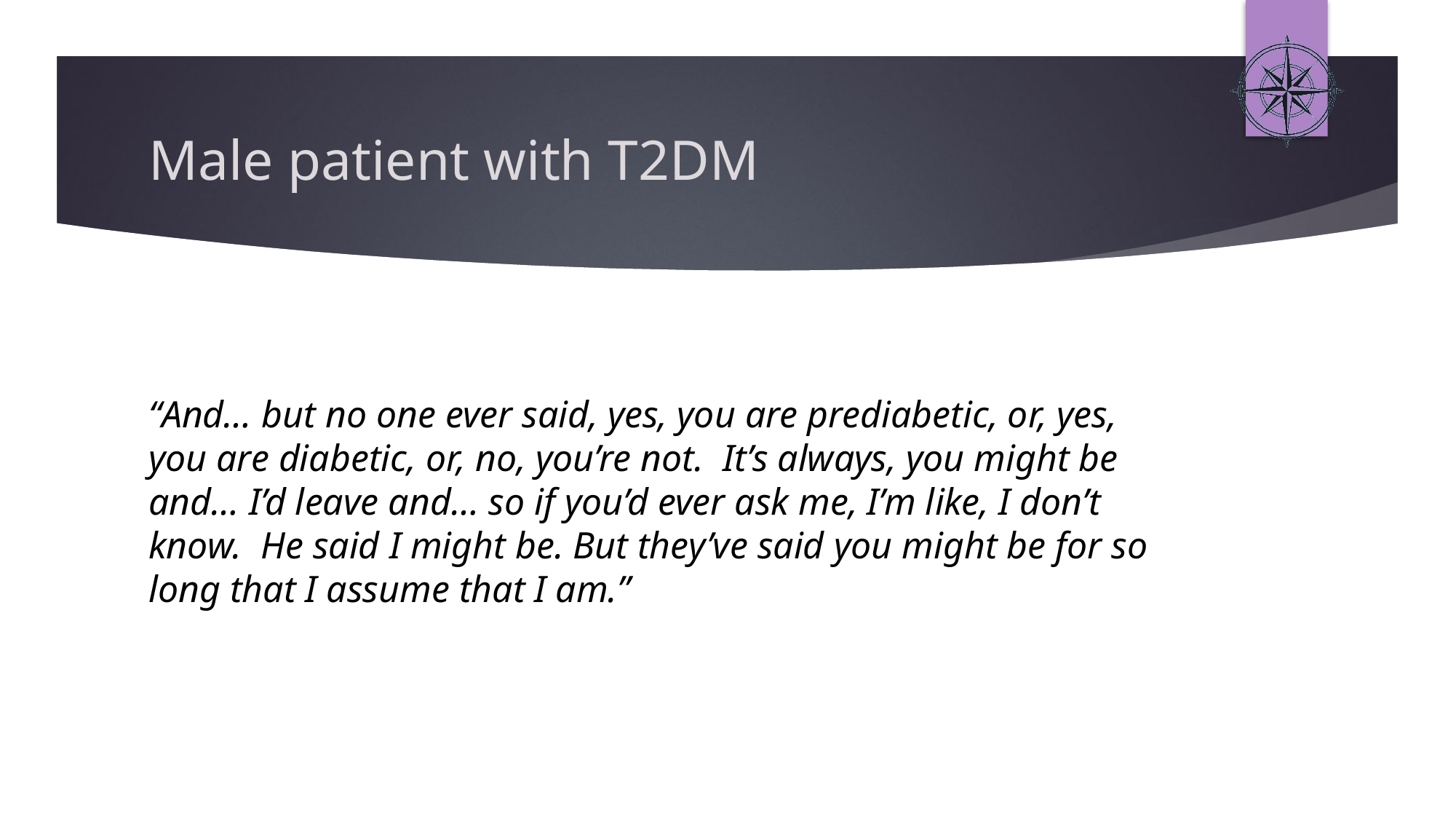

# Male patient with T2DM
“And… but no one ever said, yes, you are prediabetic, or, yes, you are diabetic, or, no, you’re not. It’s always, you might be and… I’d leave and… so if you’d ever ask me, I’m like, I don’t know. He said I might be. But they’ve said you might be for so long that I assume that I am.”

## Slide 18
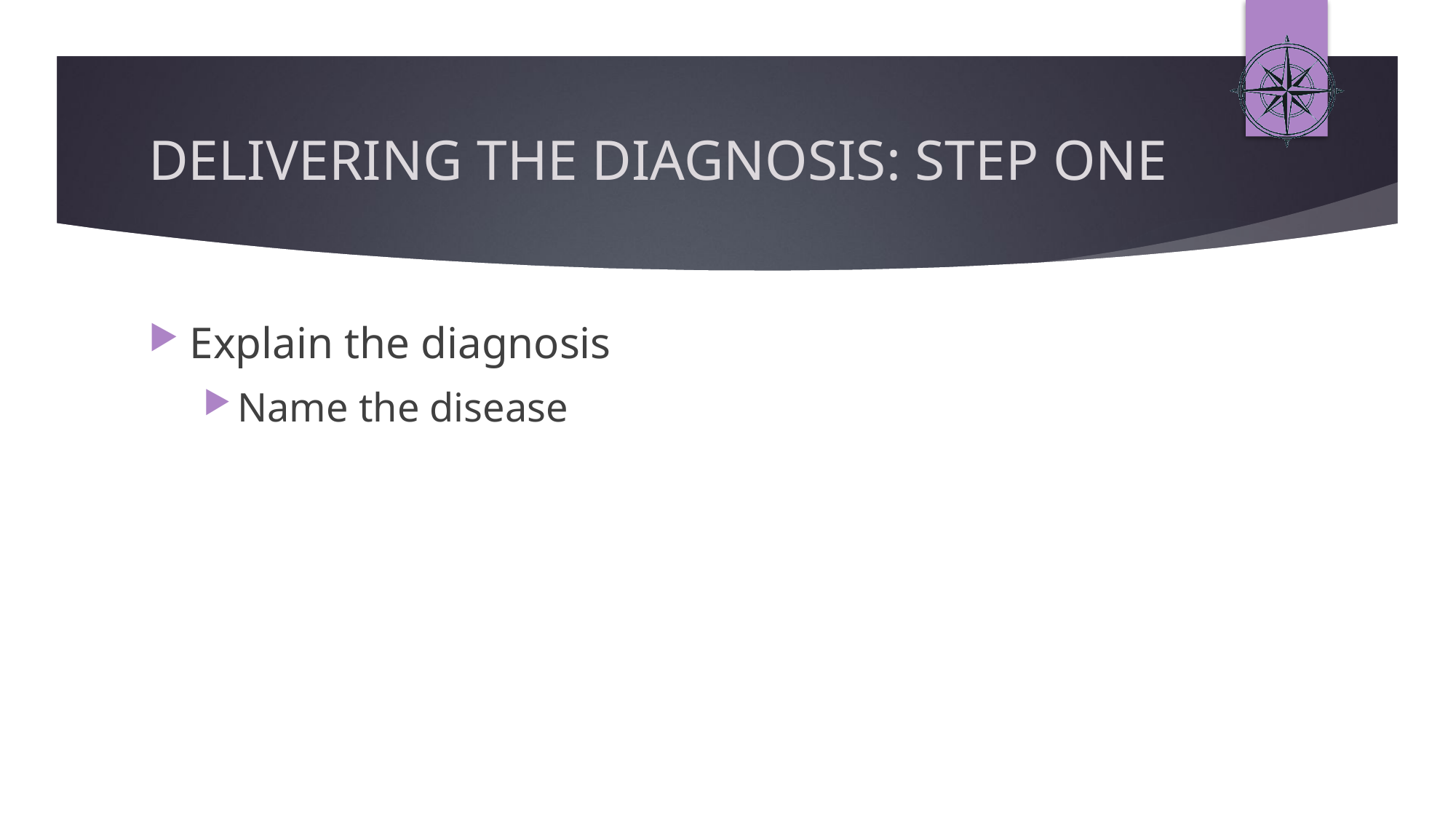

# DELIVERING THE DIAGNOSIS: STEP ONE
Explain the diagnosis
Name the disease

## Slide 19
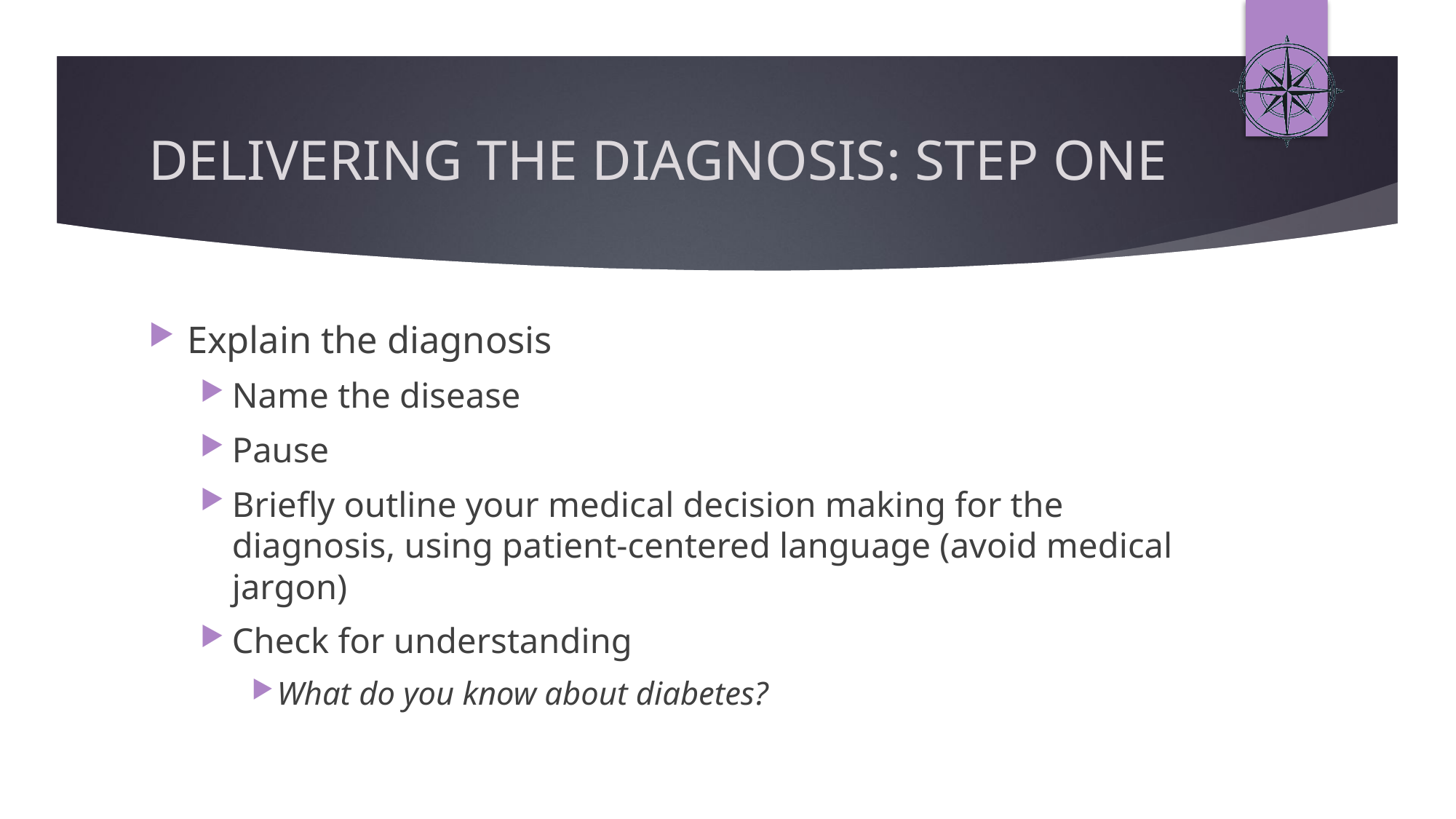

# DELIVERING THE DIAGNOSIS: STEP ONE
Explain the diagnosis
Name the disease
Pause
Briefly outline your medical decision making for the diagnosis, using patient-centered language (avoid medical jargon)
Check for understanding
What do you know about diabetes?

## Slide 20
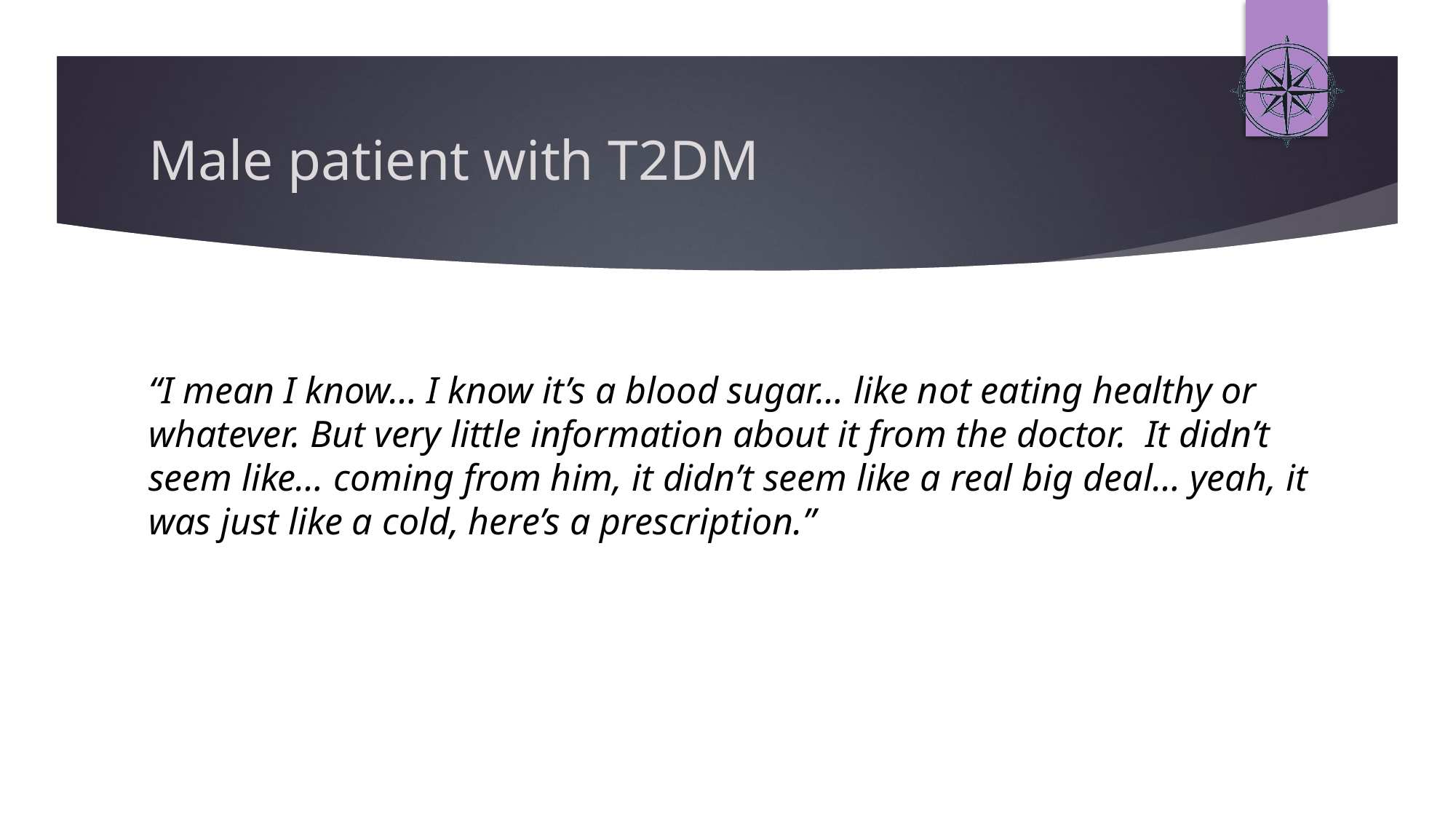

# Male patient with T2DM
“I mean I know… I know it’s a blood sugar… like not eating healthy or whatever. But very little information about it from the doctor. It didn’t seem like… coming from him, it didn’t seem like a real big deal… yeah, it was just like a cold, here’s a prescription.”

## Slide 21
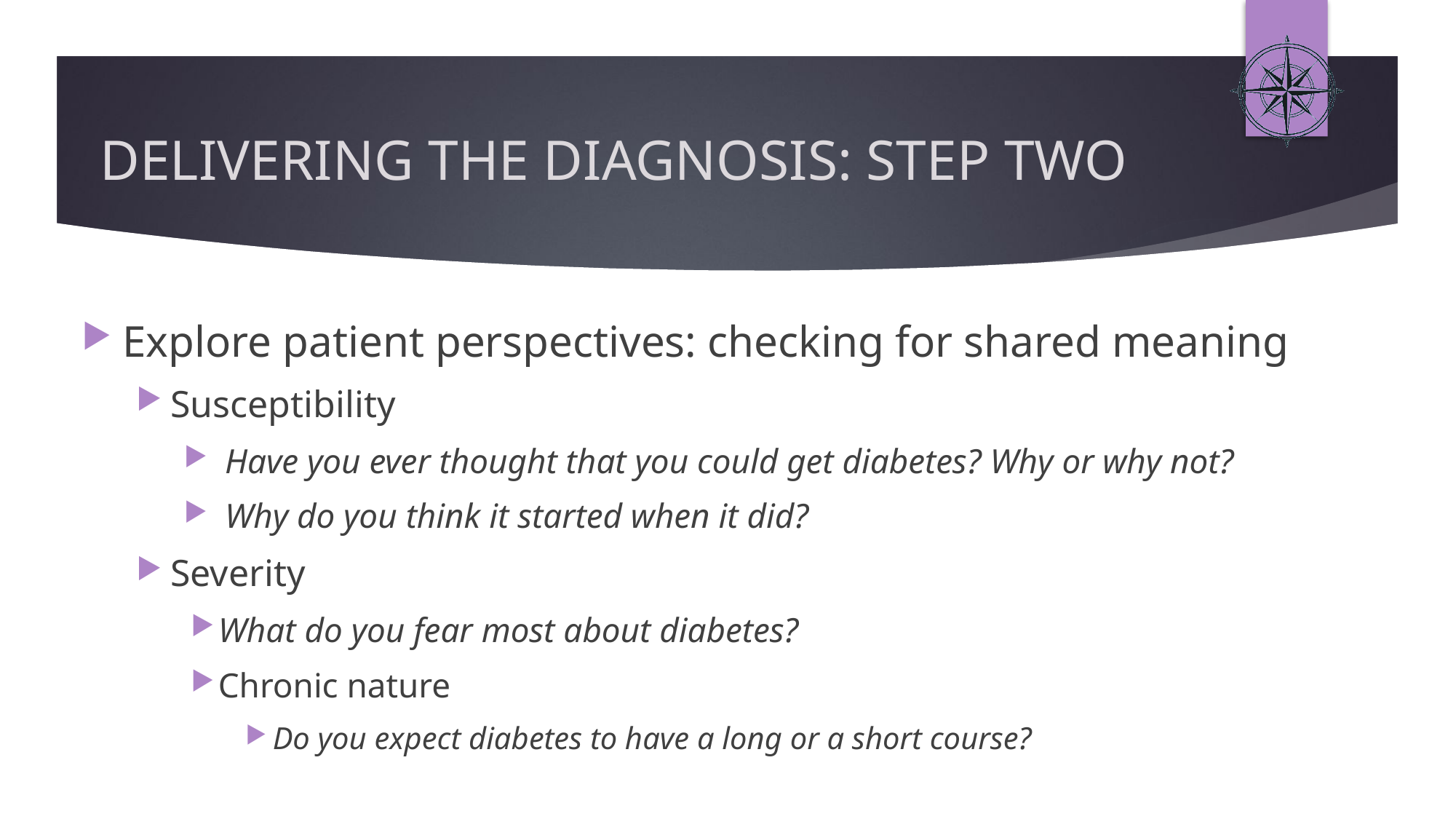

# DELIVERING THE DIAGNOSIS: STEP TWO
Explore patient perspectives: checking for shared meaning
Susceptibility
Have you ever thought that you could get diabetes? Why or why not?
Why do you think it started when it did?
Severity
What do you fear most about diabetes?
Chronic nature
Do you expect diabetes to have a long or a short course?

## Slide 22
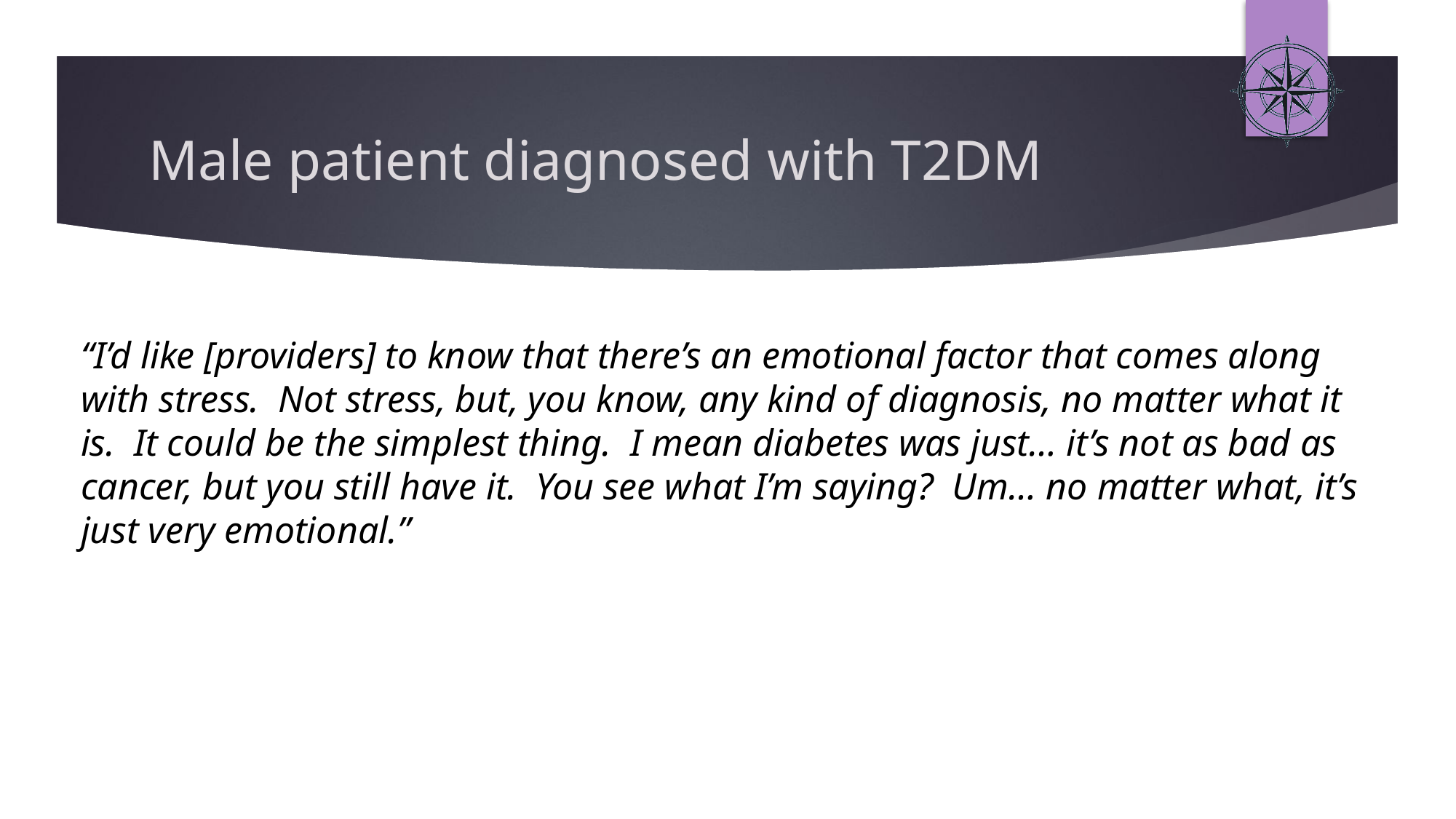

# Male patient diagnosed with T2DM
“I’d like [providers] to know that there’s an emotional factor that comes along with stress. Not stress, but, you know, any kind of diagnosis, no matter what it is. It could be the simplest thing. I mean diabetes was just… it’s not as bad as cancer, but you still have it. You see what I’m saying? Um… no matter what, it’s just very emotional.”

## Slide 23
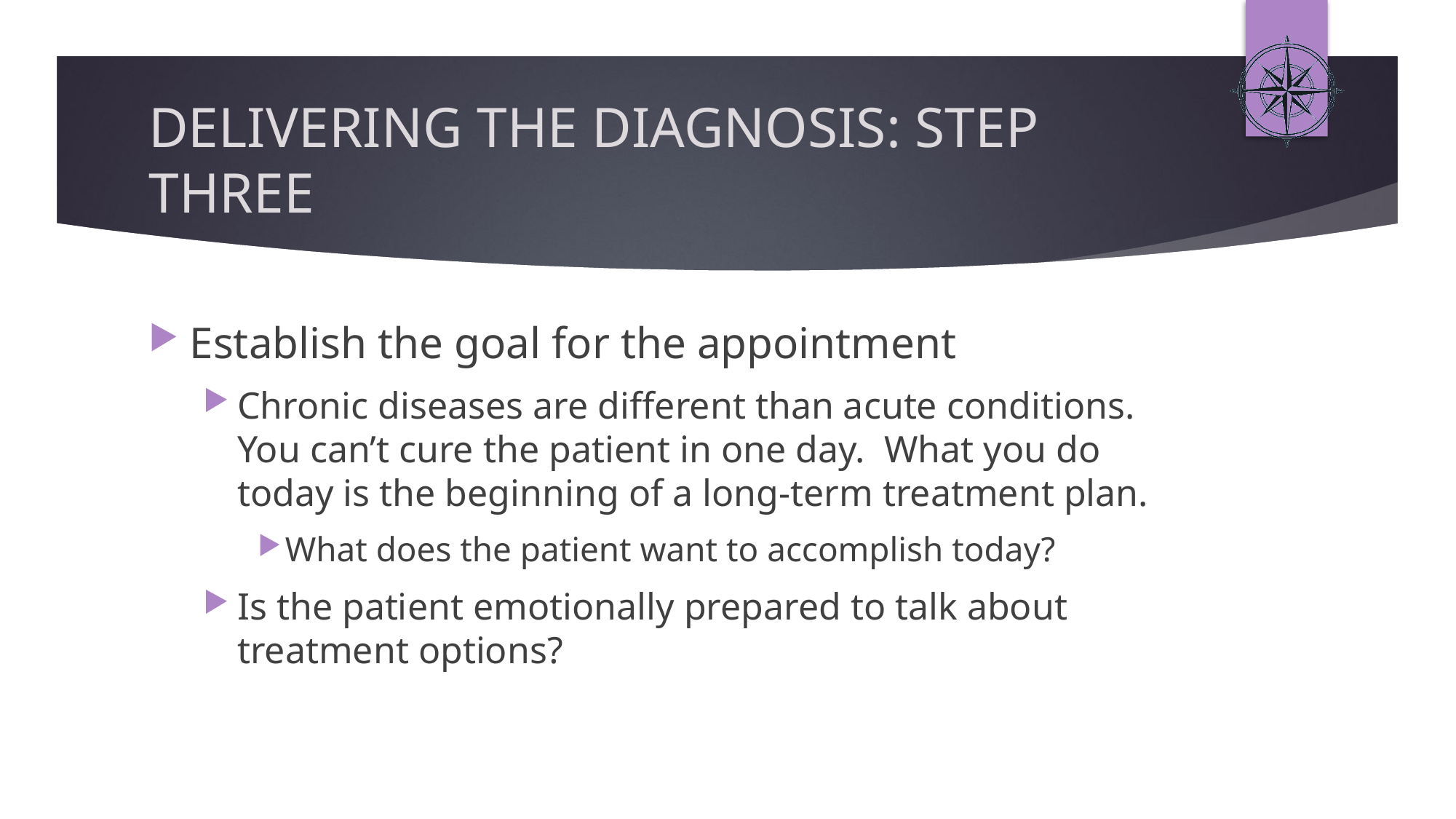

# DELIVERING THE DIAGNOSIS: STEP THREE
Establish the goal for the appointment
Chronic diseases are different than acute conditions. You can’t cure the patient in one day. What you do today is the beginning of a long-term treatment plan.
What does the patient want to accomplish today?
Is the patient emotionally prepared to talk about treatment options?

## Slide 24
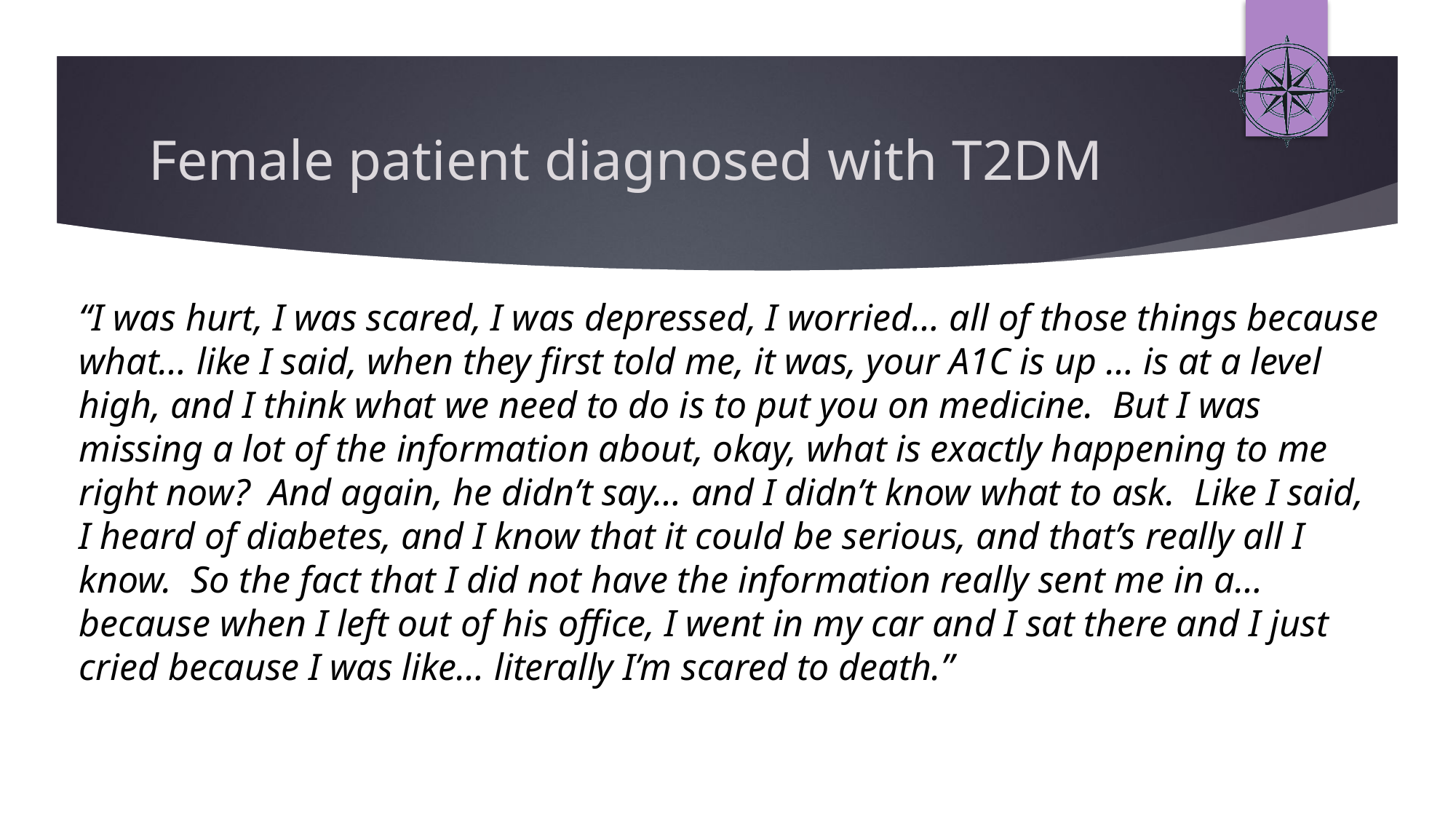

# Female patient diagnosed with T2DM
“I was hurt, I was scared, I was depressed, I worried… all of those things because what… like I said, when they first told me, it was, your A1C is up … is at a level high, and I think what we need to do is to put you on medicine. But I was missing a lot of the information about, okay, what is exactly happening to me right now? And again, he didn’t say… and I didn’t know what to ask. Like I said, I heard of diabetes, and I know that it could be serious, and that’s really all I know. So the fact that I did not have the information really sent me in a…because when I left out of his office, I went in my car and I sat there and I just cried because I was like… literally I’m scared to death.”

## Slide 25
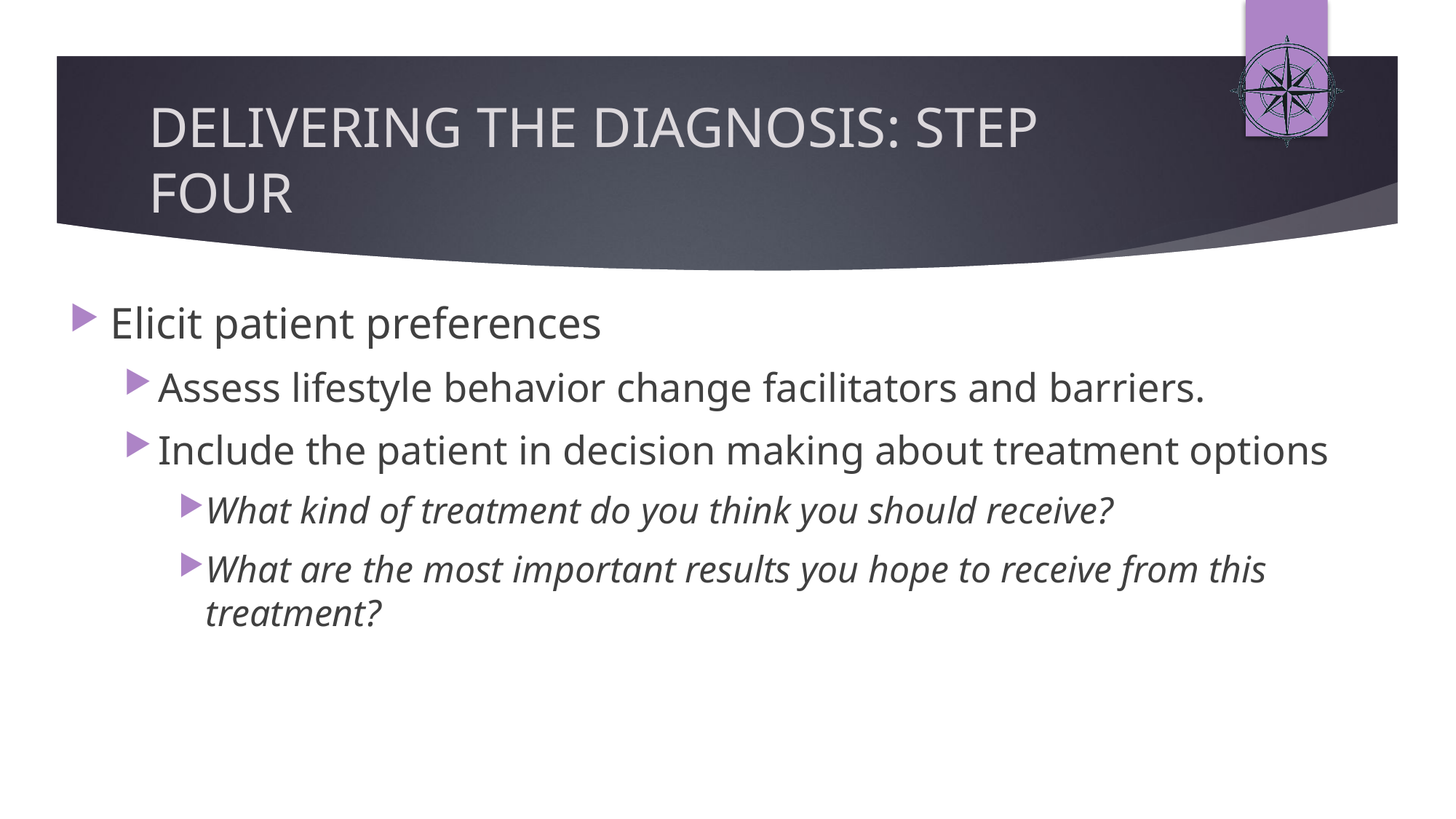

# DELIVERING THE DIAGNOSIS: STEP FOUR
Elicit patient preferences
Assess lifestyle behavior change facilitators and barriers.
Include the patient in decision making about treatment options
What kind of treatment do you think you should receive?
What are the most important results you hope to receive from this treatment?

## Slide 26
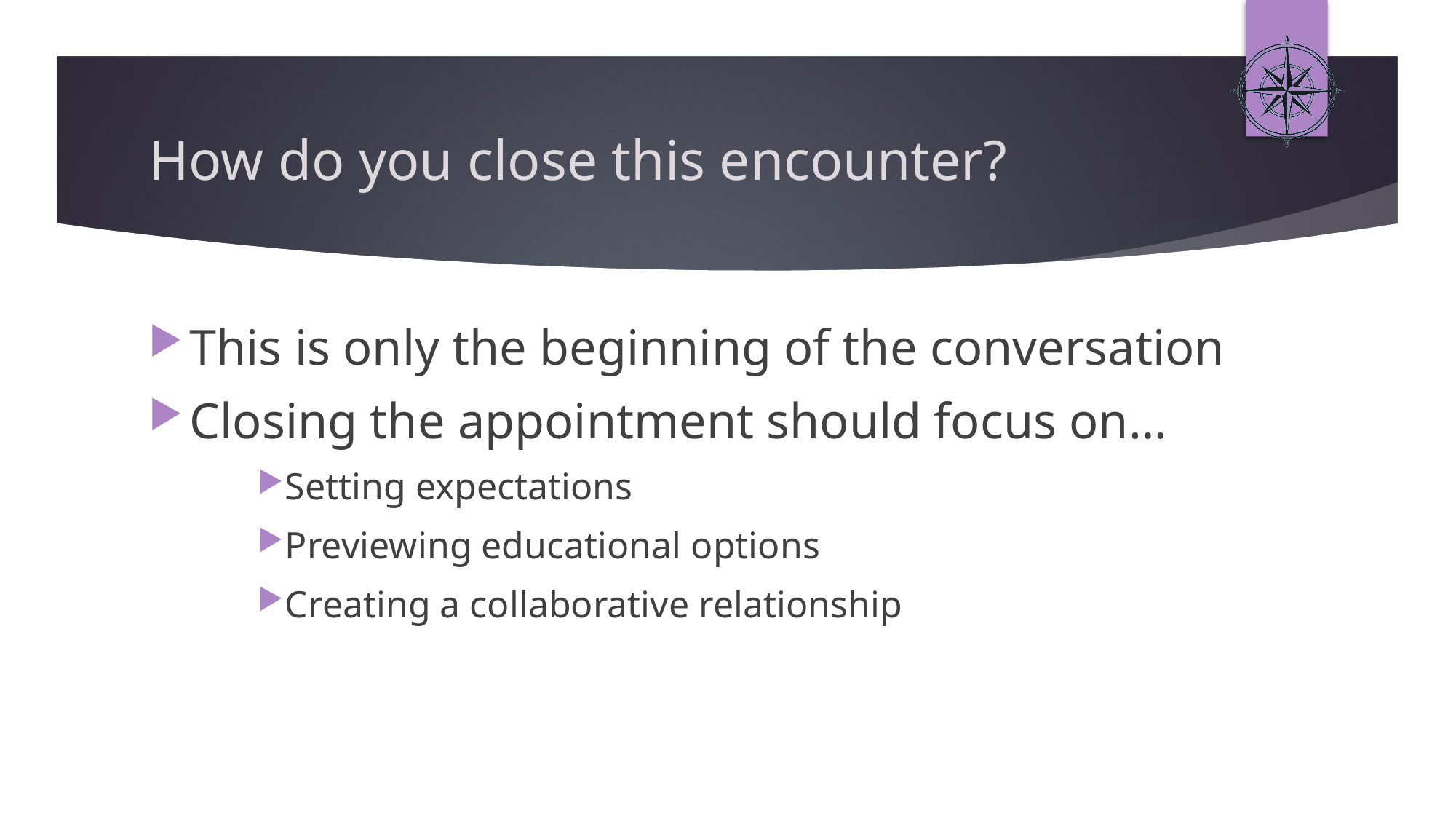

# How do you close this encounter?
This is only the beginning of the conversation
Closing the appointment should focus on…
Setting expectations
Previewing educational options
Creating a collaborative relationship

## Slide 27
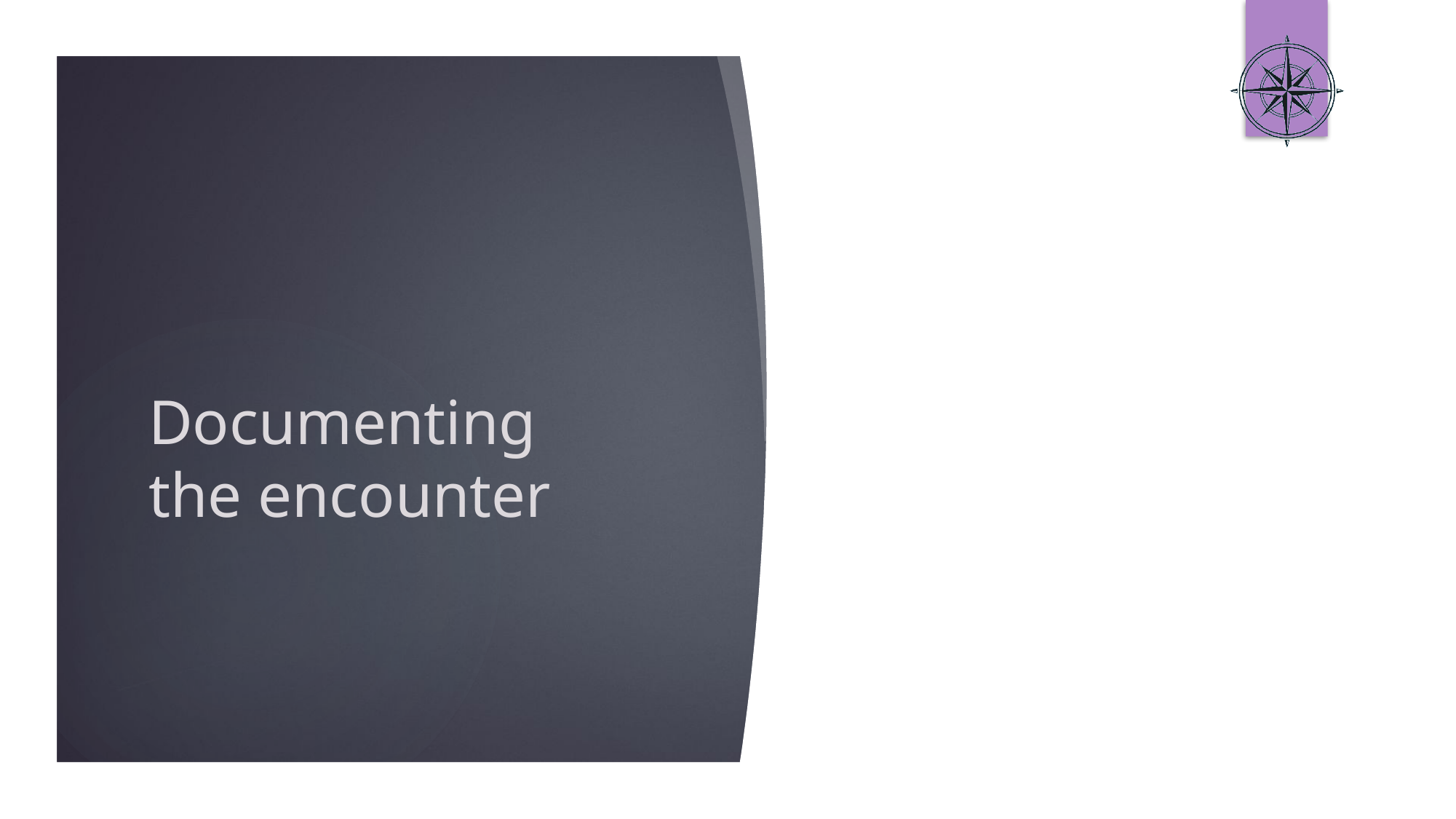

# Documenting the encounter

## Slide 28
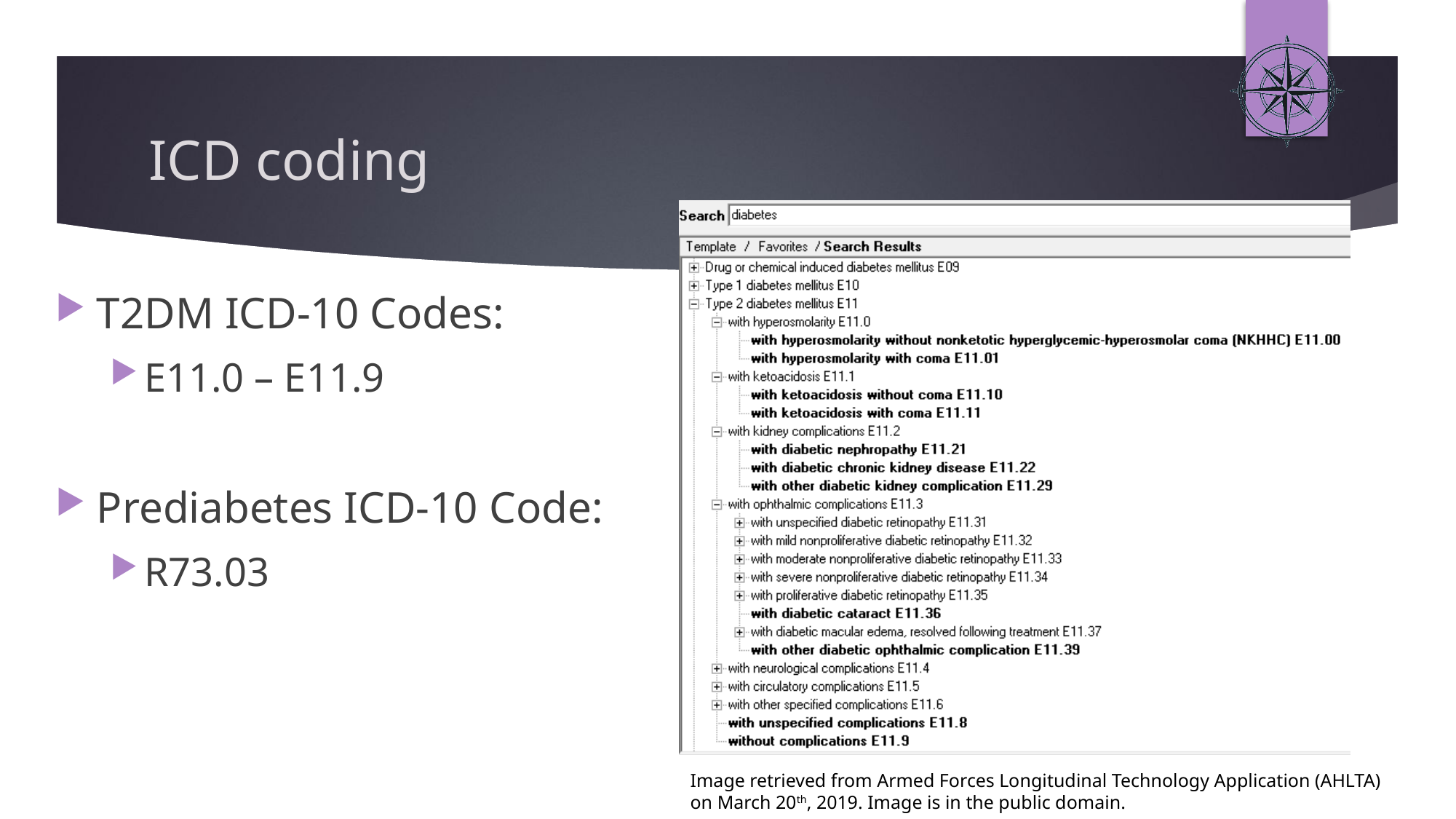

# ICD coding
T2DM ICD-10 Codes:
E11.0 – E11.9
Prediabetes ICD-10 Code:
R73.03
Image retrieved from Armed Forces Longitudinal Technology Application (AHLTA) on March 20th, 2019. Image is in the public domain.

## Slide 29
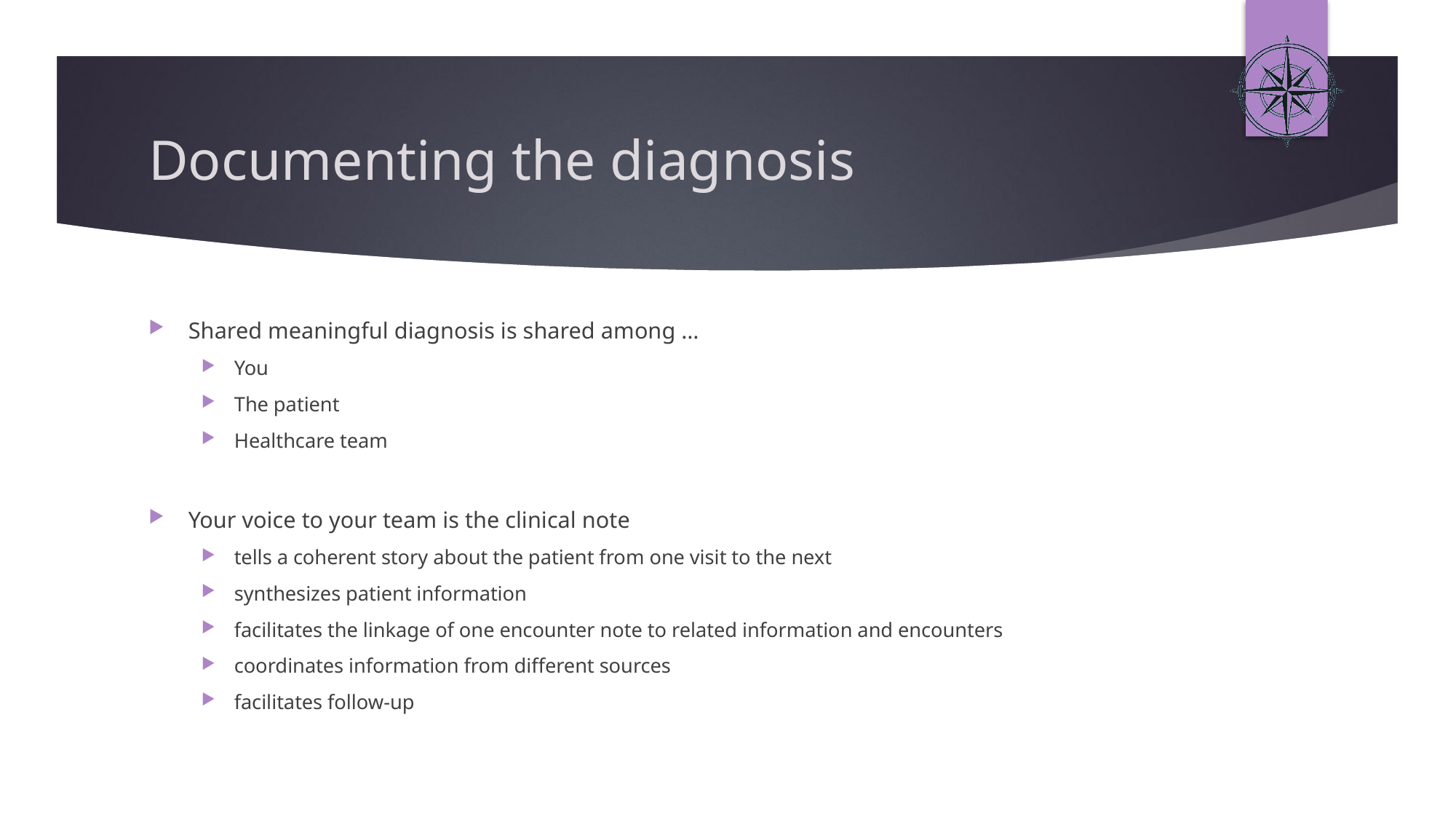

# Documenting the diagnosis
Shared meaningful diagnosis is shared among …
You
The patient
Healthcare team
Your voice to your team is the clinical note
tells a coherent story about the patient from one visit to the next
synthesizes patient information
facilitates the linkage of one encounter note to related information and encounters
coordinates information from different sources
facilitates follow-up

## Slide 30
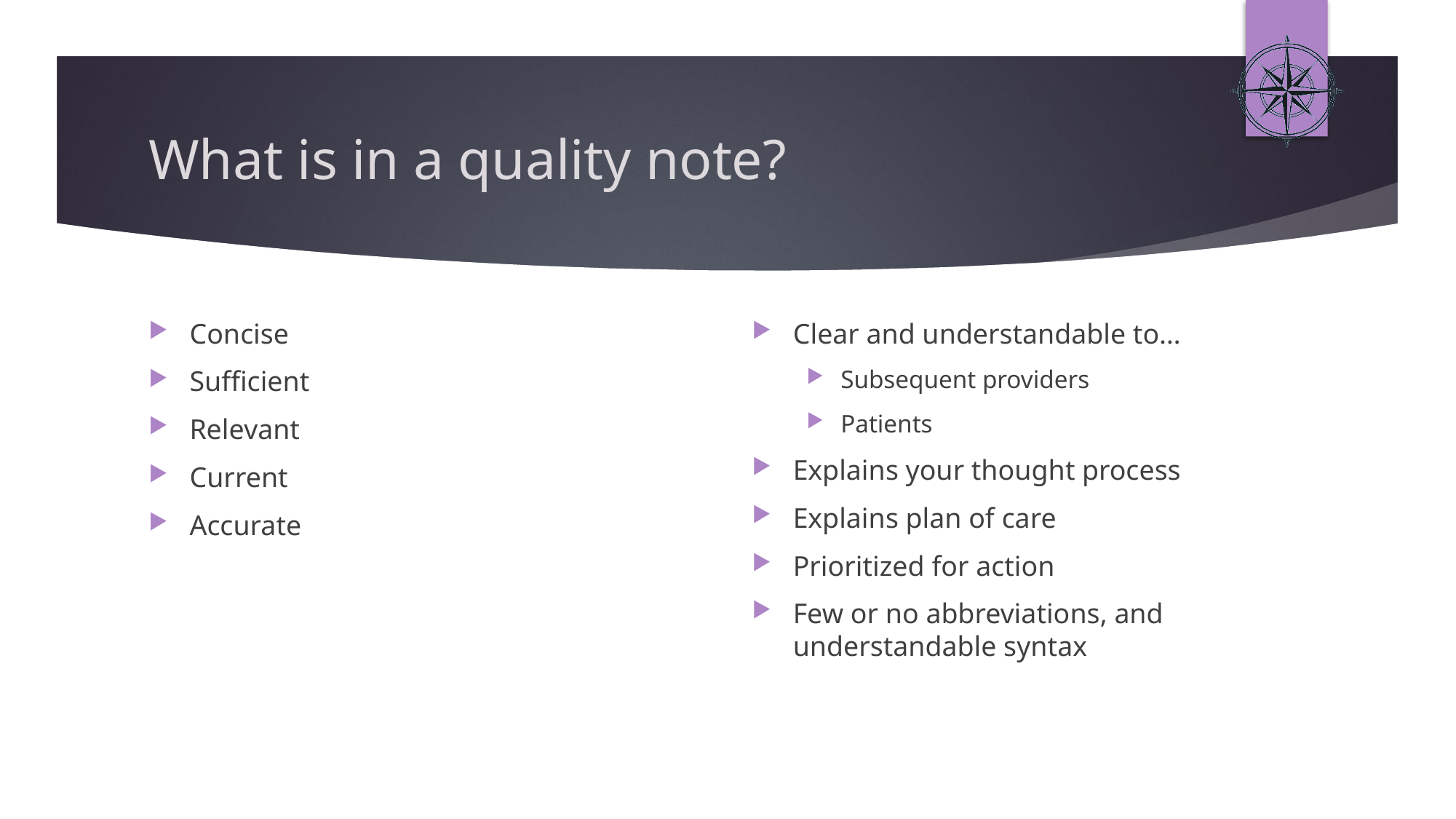

# What is in a quality note?
Concise
Sufficient
Relevant
Current
Accurate
Clear and understandable to…
Subsequent providers
Patients
Explains your thought process
Explains plan of care
Prioritized for action
Few or no abbreviations, and understandable syntax

## Slide 31
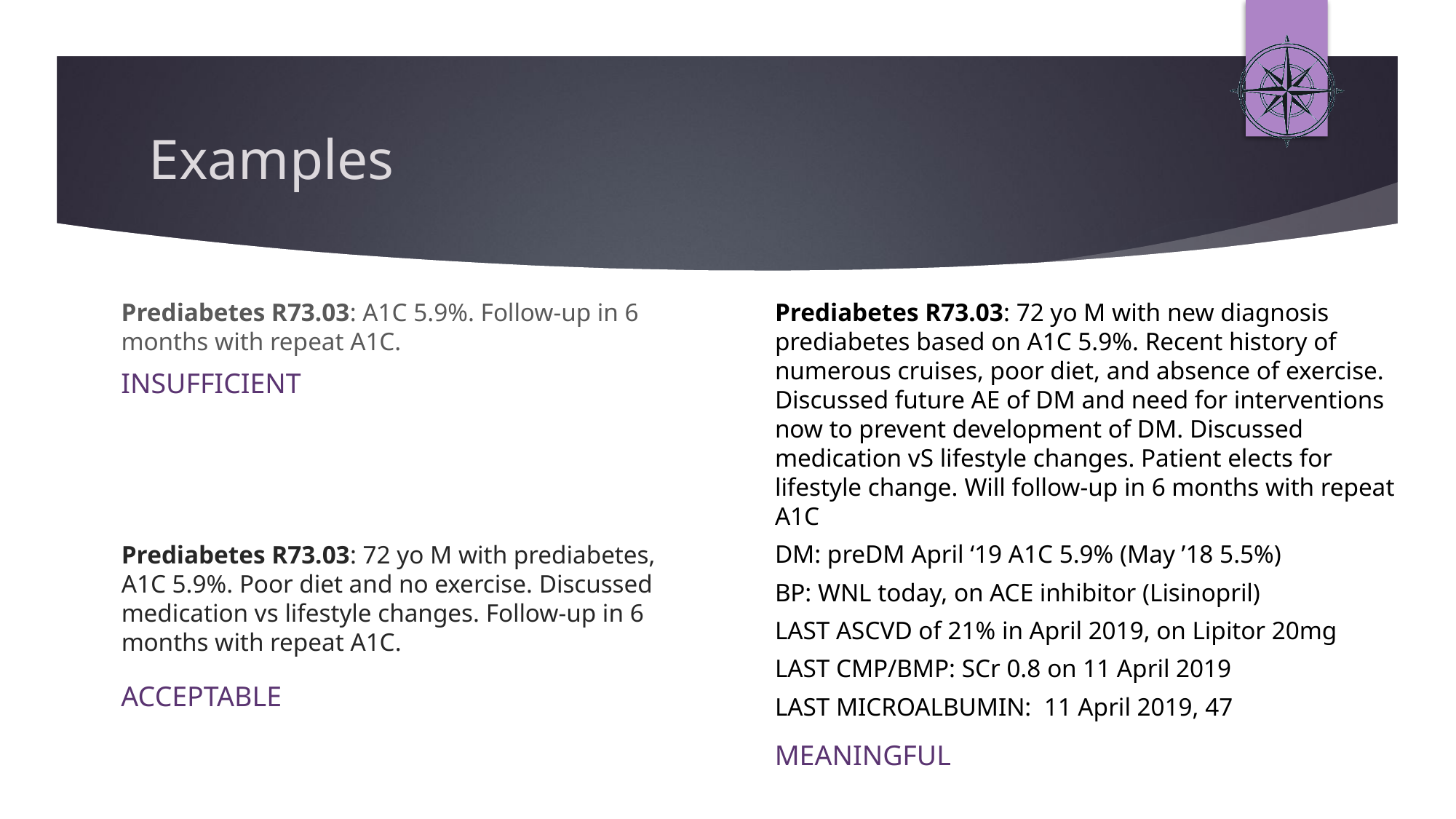

# Examples
Prediabetes R73.03: 72 yo M with new diagnosis prediabetes based on A1C 5.9%. Recent history of numerous cruises, poor diet, and absence of exercise. Discussed future AE of DM and need for interventions now to prevent development of DM. Discussed medication vS lifestyle changes. Patient elects for lifestyle change. Will follow-up in 6 months with repeat A1C
DM: preDM April ‘19 A1C 5.9% (May ’18 5.5%)
BP: WNL today, on ACE inhibitor (Lisinopril)
LAST ASCVD of 21% in April 2019, on Lipitor 20mg
LAST CMP/BMP: SCr 0.8 on 11 April 2019
LAST MICROALBUMIN: 11 April 2019, 47
Prediabetes R73.03: A1C 5.9%. Follow-up in 6 months with repeat A1C.
INSUFFICIENT
Prediabetes R73.03: 72 yo M with prediabetes, A1C 5.9%. Poor diet and no exercise. Discussed medication vs lifestyle changes. Follow-up in 6 months with repeat A1C.
ACCEPTABLE
MEANINGFUL

## Slide 32
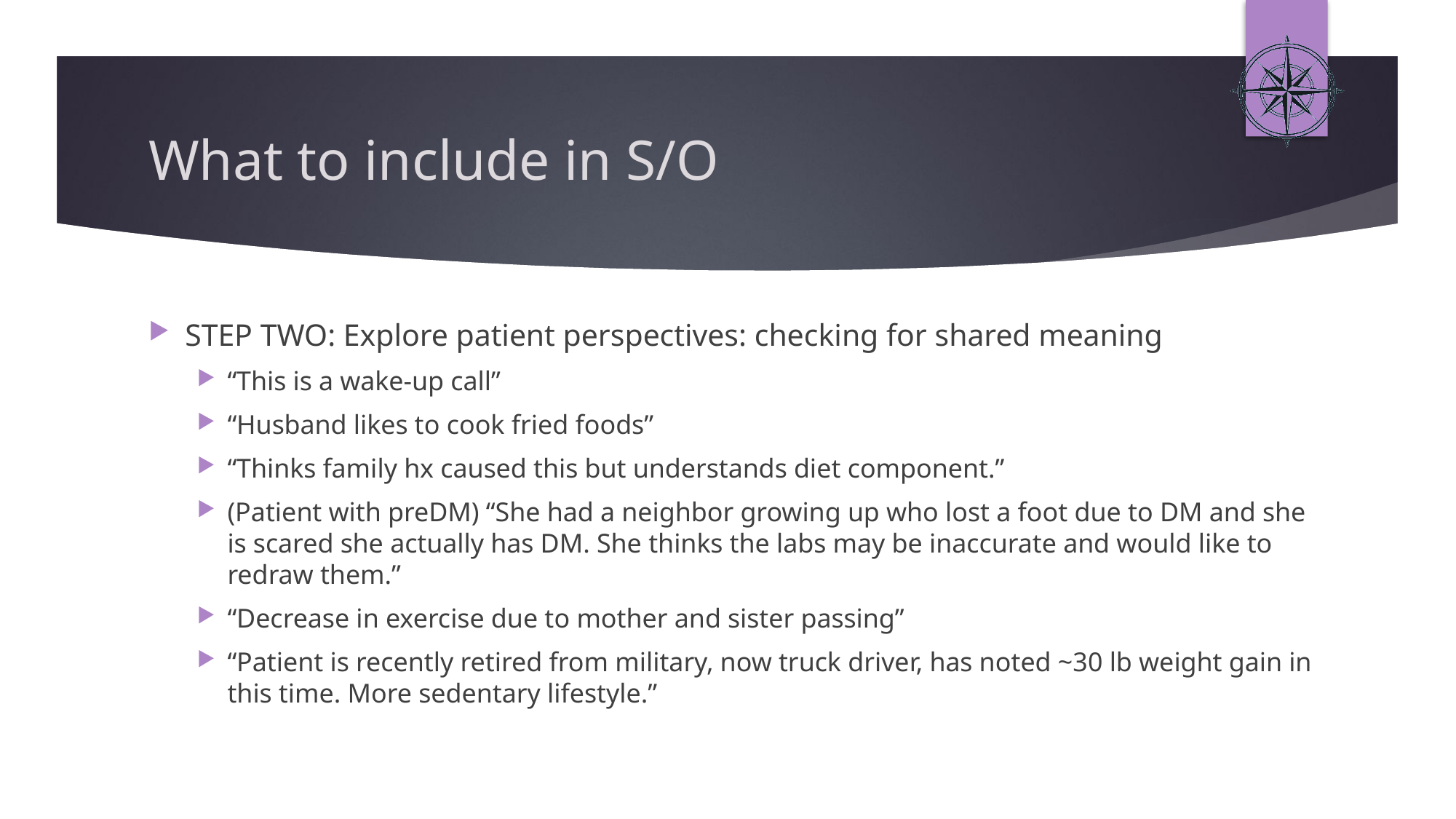

# What to include in S/O
STEP TWO: Explore patient perspectives: checking for shared meaning
“This is a wake-up call”
“Husband likes to cook fried foods”
“Thinks family hx caused this but understands diet component.”
(Patient with preDM) “She had a neighbor growing up who lost a foot due to DM and she is scared she actually has DM. She thinks the labs may be inaccurate and would like to redraw them.”
“Decrease in exercise due to mother and sister passing”
“Patient is recently retired from military, now truck driver, has noted ~30 lb weight gain in this time. More sedentary lifestyle.”

## Slide 33
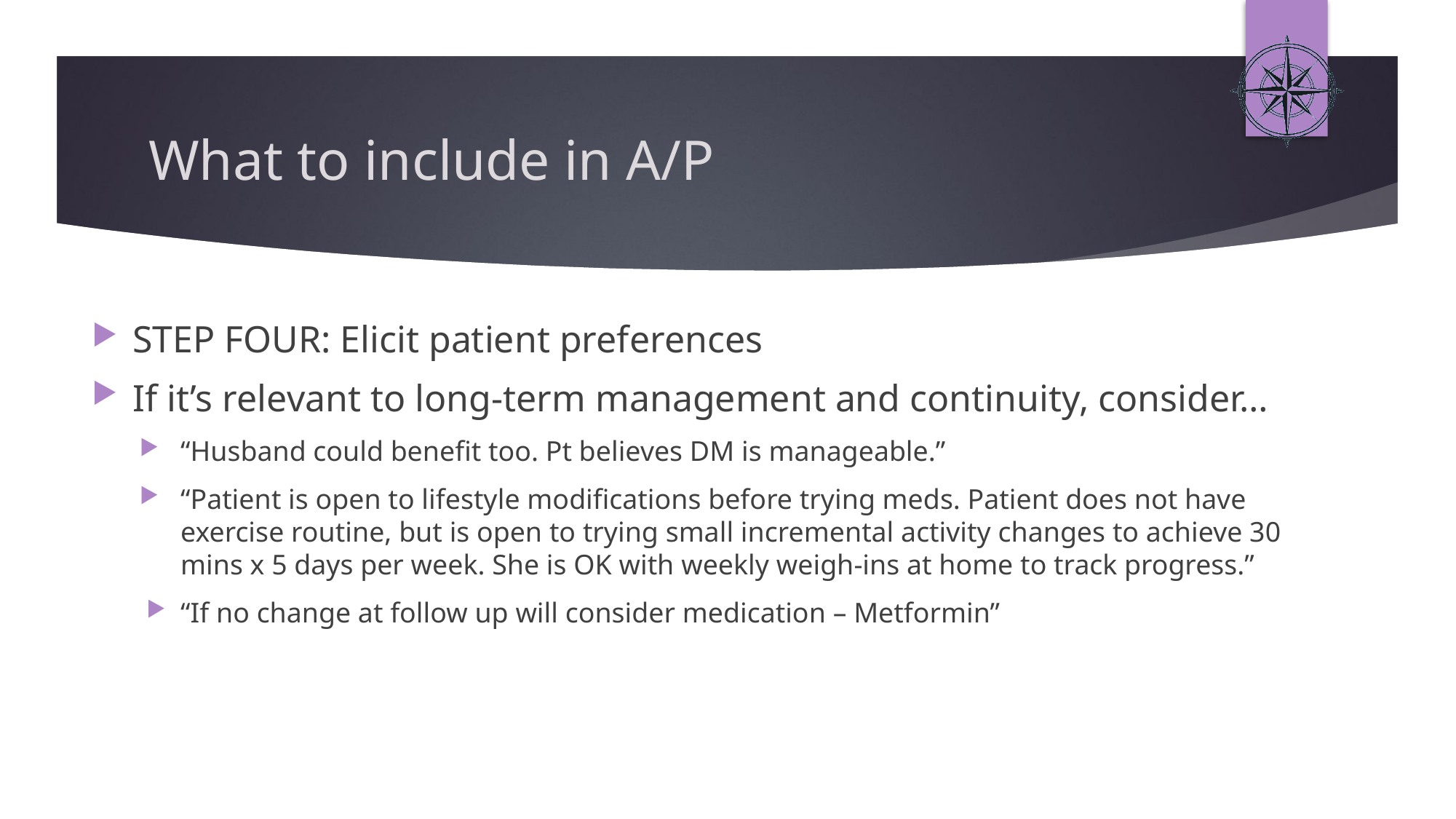

# What to include in A/P
STEP FOUR: Elicit patient preferences
If it’s relevant to long-term management and continuity, consider…
“Husband could benefit too. Pt believes DM is manageable.”
“Patient is open to lifestyle modifications before trying meds. Patient does not have exercise routine, but is open to trying small incremental activity changes to achieve 30 mins x 5 days per week. She is OK with weekly weigh-ins at home to track progress.”
“If no change at follow up will consider medication – Metformin”

## Slide 34
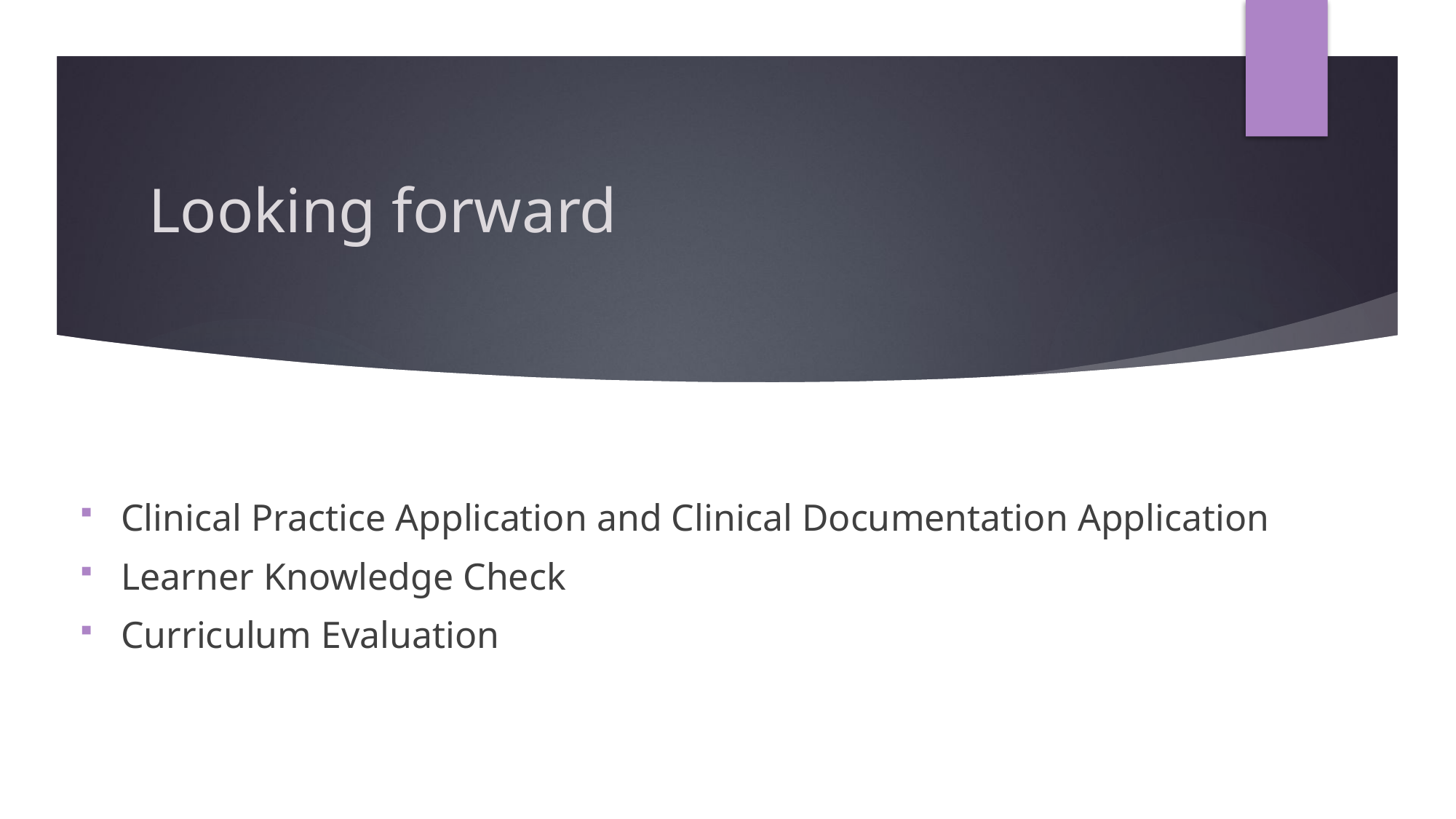

# Looking forward
Clinical Practice Application and Clinical Documentation Application
Learner Knowledge Check
Curriculum Evaluation
