# Supplementary material for: Diabetes ROADMAP: Teaching Guideline Use, Communication, and Documentation When Delivering the Diagnosis of Diabetes
Source: MedEdPORTAL. 2020 Sep 11;16:10959. doi: 10.15766/mep_2374-8265.10959 (PMC7485911; doi:10.15766/mep_2374-8265.10959)
Supplement: Supplementary file 1 — Curriculum Overview.pdfTeaching Guide.pdfROADMAP Presentation.pptxFacilitator Guide.pdfSimulation Resources.pdfAssessment Tools.pdf [file mep_2374-8265.10959-s001.zip › D. Facilitator Guide.pdf]

ROADMAP

FACILITATOR GUIDE

Uniformed Services University of the Health Sciences

MPCRN

MILITARY PRIMARY CARE RESEARCH NETWORK

Authors include: Christy JW Ledford, Lauren Cafferty, Heather Rider, Stephanie Fulleborn, Erik Clauson, Steven Trigg, and Christopher Ledford.

Overview

Thank you for your willingness to serve as a facilitator in the ROADMAP intervention. The aim is to prepare clinicians for the challenge of talking to patients about a new diabetes or prediabetes diagnosis. Through our work in both medical education and diabetes education, we find that the most effective teaching model is to follow educational instruction with learner opportunities to discuss new knowledge with their peers. We take a learner-centered approach, which encourages sharing of personal and professional experiences related to the context; fosters a supportive learning climate that promotes risk-taking; engages learners in interactive experiences; and attends to relationship-building among participants.

Our learning objectives for today are:

- to list and describe the current American Diabetes Association (ADA) guidelines on screening, diagnosis, and treatment of prediabetes and type 2 diabetes;
- to identify the relationship between the diagnosis moment and potential for patient behavior change;
- to demonstrate how to establish shared meaning when communicating a new diabetes diagnosis; and
- to document a meaningful diabetes diagnosis in the patient health record.

You, as the facilitator, will lead the small group. Facilitators don't need to be content experts, but it will be helpful to review the teaching slides prior to leading the activity. This role is about group dynamics and interpersonal management. Social skills are important for facilitation. Try gentle interruption and probing questions to keep the group on task.

SCHEDULE

Programmatic overview of curriculum as graduate medical education (2 hours)

Activity	Objective	Format	Time
Medical decision making lecture	To present the current* guidelines for screening and treatment of prediabetes and type 2 diabetes.	Large group	15 mins
Interpersonal communication lecture	To teach how clinicians can facilitate patient conversations that lead to shared meaning of the diabetes diagnosis.		25 mins
Role play	To apply and practice skills in a supportive learning climate of observation and feedback.	Small group	50 mins
Clinical documentation lecture	To demonstrate how to incorporate information from the four steps into the patient's story, as documented in the health record.	Large group	15 mins
With personal practice and application…			
Clinical practice application	To reflect on application of skills applied in clinical practice.	One on one	10 mins

*The 2019 ADA guidelines are presented in this curriculum guide.

FACILITATOR TIPS

- Remember that you are here to facilitate discussion among the learners, not to give them the "right answer."
- Remind learners that this is a safe place to take risks and to be constructive in feedback.
- The learners may fall back into the comfort of talking about the patho-physiological explanation for diabetes. In the small groups, try to steer them away from this. The curriculum is about talking to patients about the full biopsychosocial explanation of the disease.
- This is an opportunity for brainstorming new ways to talk about diabetes and provide each other feedback.

Role play instructions

This activity will focus on brainstorming different words to use in a clinical encounter when applying what the learners have seen in the teaching session. Prior to the activity, learners will be split into groups, and you will be the facilitator for one of those groups. The following information serves as a guide to help you instruct learners how to participate in the activity and includes a page (Facilitator notes, p. 36) for notetaking during the activity. The instructions are numbered to give you each step of the activity. Italicized text is recommended scripted language for the facilitator.

1. Introduce yourself and outline your role as a facilitator.

> *"I want to remind you that there are no grades and I am not here to evaluate you. This is a learning opportunity and experimental – a time to try things out where the stakes are very low."*

---

2. Read the learning objectives:

> *"Our learning objectives today are:*
>
> *to list and describe the current ADA guidelines on screening, diagnosis, and treatment of prediabetes and type 2 diabetes;*
>
> *to identify the relationship between the diagnosis moment and potential for patient behavior change;*
>
> *to demonstrate how to establish shared meaning when communicating a new diabetes diagnosis; and*
>
> *to document a meaningful diabetes diagnosis in the patient health record."*

---

3. Read the instructions for enacting the small group activity:

> *"In the teaching session, you learned about 4 steps of the clinical encounter (1- explain the diagnosis, 2- explore patient perceptions, 3- establish goals for the appointment 4- elicit patient preferences).*
>
> *I will give you a patient scenario, and you are going to role-play the clinical encounter to practice the words you might use to move through each step. Try saying the first thing that pops in your head. Feel the freedom to practice different ways of phrasing the diagnosis or*

presenting next steps to see what you like or what feels most comfortable. One person will play the role of the physician, one will act the role of the patient.

While the physician and patient are role playing, the rest of the group will use the observation worksheet that I will give you for notes. Once the encounter is completed (about 15 minutes), feedback will be shared by the observers, and then another pair will be selected to role play.

We will continue rotating through pairs until we have run out of time."

---

4. Pass out the ROLE PLAY OBSERVATION page to the learners in your small group. Assign the first pair to act in the roles of physician and patient. Give the "patient" the paper with the appropriate role play scenario.

   To the "patient":

   "Read the role play scenario in front of you to yourself."

   To the "physician":

   "The scenario does not specify a gender or race/ethnicity so that you can imagine the 'patient' as the person in front of you."

   To the observers:

   "Observers, please use the ROLE PLAY OBSERVATION page to write down specific words or phrases that you might want to incorporate into your practice or note something you might change within the four steps."

   ---

5. Three scenarios are provided to allow for up to three interactions during this activity. As you move through the scenarios, read the corresponding prompt aloud to the entire group. Take notes during each step to jog your memory for the discussion and to help keep discussion on track. A facilitator notes structure is on page 36; this structure mirrors the ROLE PLAY OBSERVATION page.

   Scenario 1 [Diabetes]:

   "Patient labs and vitals are as follows. A1c drawn last week was 7.1. BMI today is 30. Blood pressure today is 127/77."

Scenario 2 [Prediabetes]:

"Patient labs and vitals are as follows. A1c drawn last week was 5.8. BMI today is 29. Blood pressure today is 131/86."

Scenario 3 [Diabetes]:

"Patient labs and vitals are as follows. A1c drawn last week was 7.4. BMI today is 31. Blood pressure today is 131/88."

---

6. Once the encounter is complete (about 10-15 minutes), transition to a group discussion.

"Now we want to take about 10 minutes to exchange ideas and feedback."

If discussion is slow to start, consider these prompting questions:

Were all the steps apparent in the encounter?
Which step was the most challenging to incorporate into the encounter?
What did you hear that you would incorporate into your practice?
What would you have phrased differently?
What words would you have used to explain the diagnosis?

7. As time allows, walk through the next scenario. Select different learners to play the roles each time. The goal is to complete all three role play scenarios.

"Now, let's try another pair of learners with a different role play scenario."

REPEAT STEPS 4 - 6.

8. When time is up for the small group activity, transition back to the large group for the clinical documentation lecture.

Facilitator notes

Step	Notes		
	Scenario 1	Scenario 2	Scenario 3
**Explain the diagnosis**: Name the condition, clearly explain what it is, and check for understanding.			
**Explore patient perceptions**: Check for shared meaning, including severity and susceptibility.			
**Establish goal for today's appointment**: Knowing diabetes is a chronic condition, determine what you and the patient can accomplish today.			
**Elicit patient preferences**: Include the patient in decision making about treatment options and assessing behavior change facilitators and barriers.			

ROLE PLAY SCENARIOS

Scenario 1

You are 51 years old. *Your appointment today is to discuss the results of your lab work* from last week, which was ordered as part of your annual routine physical. Today, your doctor will diagnose you with **diabetes**. You have no family history of diabetes but you have seen people who have it and recognize that they don't take care of themselves. You exercise regularly and eat healthy, so you are shocked by this diagnosis.

✂---

Scenario 2

You are 51 years old. *Your appointment today is to discuss the results of your lab work* from last week, which was ordered as part of your annual routine physical. Today, your doctor will diagnose you with **prediabetes**. Although your mother was diagnosed with type 2 diabetes 15 years ago and you are very familiar with the effect it has had on her, you have never heard the term "prediabetes." You are confused and really don't want to have to be on another medicine. You are currently on a medication for high blood pressure.

✂---

Scenario 3

You are 51 years old. *Your appointment today is to discuss the results of your lab work* from last week, which was ordered as part of your annual routine physical. Today, your doctor will diagnose you with **diabetes**. You are scared. Your grandmother and uncle both had type 2 diabetes. Your grandmother's foot had to be amputated, and she passed away a year later following a heart attack. If medicine will help, you are willing to take it to cure this disease and continue with life as it is now. You are already on a medication for your high blood pressure.

ROLE PLAY OBSERVATION

Step	Notes		
	Scenario 1	Scenario 2	Scenario 3
**Explain the diagnosis**: Name the condition, clearly explain what it is, and check for understanding.			
**Explore patient perceptions**: Check for shared meaning, including severity and susceptibility.			
**Establish goal for today's appointment**: Knowing diabetes is a chronic condition, determine what you and the patient can accomplish today.			
**Elicit patient preferences**: Include the patient in decision making about treatment options and assessing behavior change facilitators and barriers.			
