# Supplementary material for: Diabetes ROADMAP: Teaching Guideline Use, Communication, and Documentation When Delivering the Diagnosis of Diabetes
Source: MedEdPORTAL. 2020 Sep 11;16:10959. doi: 10.15766/mep_2374-8265.10959 (PMC7485911; doi:10.15766/mep_2374-8265.10959)
Supplement: Supplementary file 1 — Curriculum Overview.pdfTeaching Guide.pdfROADMAP Presentation.pptxFacilitator Guide.pdfSimulation Resources.pdfAssessment Tools.pdf [file mep_2374-8265.10959-s001.zip › E. Simulation Resources.pdf]

ROADMAP

SIMULATION RESOURCES

Uniformed Services University of the Health Sciences

MPCRN
MILITARY PRIMARY CARE RESEARCH NETWORK

Authors include: Christy JW Ledford, Lauren Cafferty, Heather Rider, Dean Seehusen, Jasmyne Womack, Angela Seehusen, and Tyler Rogers.

Overview

Section 5 provides resources for enhancing the curriculum with clinical simulation. Included in this section are instructions for learners, practice partners, and character cases for the simulation.

OBJECTIVE

The goal of this simulation, called a *clinical rehearsal*, is to provide learners high-fidelity practice. Clinical rehearsal gives learners the opportunity to integrate new knowledge and peer feedback into a clinically-similar, patient encounter. Participating learners have not necessarily experienced a high-fidelity patient simulation, and many may only be familiar with this concept as an objective structured clinical examination/evaluation (OSCE). The approach to patient simulation in this environment is for teaching, not testing. The individuals who partner in this clinical rehearsal are called "*practice partners*" because the encounters are not standardized for grading.

PRACTICE PARTNERS

The practice partner is both passive and active. In the clinical rehearsal, the practice partner plays the role of a patient. Practice partners are actors in the clinical rehearsal playing a part, but they will also need to give actionable feedback. Practice partners can be professionally-trained standardized patients, or they can be volunteers from within your hospital system, such as Red Cross volunteers or members of your hospital's patient advisory board who want to help improve practice in your system. Practice partners should not be content experts – they're not intended to judge the accuracy of a learner's biomedical knowledge.

Set aside some time prior to the simulation activity to briefly train the practice partners on their role for the activity. Practice partner instructions (pp. 48 – 50) aid in this training. Each practice partner should be given a copy of PRACTICE PARTNER INFORMATION (pp. 53-54), which is a shortened version of the practice partner instructions.

CULTURALLY-DIVERSE CHARACTER CASES

Character cases, including patient background and clinical information, were created from in-depth interviews with a variety of patients diagnosed with prediabetes and type 2 diabetes. These character cases represent a diversity of race/ethnicity. Educators are encouraged to select the cases that best represent the clinical population of the learners in your hospital system. Prior to their training, realistically assign character cases to each practice partner.

FEEDBACK FOR LEARNERS

As a clinical rehearsal, the goal of this simulation is not evaluation. Yet, learners still need feedback to know how well they are integrating the new skills they learned. Included in this section are two tools to create structure for learner feedback:

1. The PRACTICE PARTNER FEEDBACK FORM (p. 55) provides a written form of feedback.
2. The Clinical rehearsal feedback session guide (p. 52) provides discussion questions for a facilitator to walk through with learners and practice partners in a group setting. In this

setting, learners benefit from hearing specific feedback about each step in delivering the diagnosis from multiple practice partners.

Also consider using any feedback mechanisms that your program uses in simulation feedback, such as the Patient-Centered Observation Form or Essential Elements of Communication – just remember that clinical rehearsal is for high-fidelity practice, not assessment.

SCHEDULE

This simulation can be added within the intervention day (either immediately following the role play activity or at the end of the full curricular intervention) or it can be used at a later time, potentially included in your program's already-scheduled simulation days.

Practice partner instructions

These instructions accompany the PRACTICE PARTNER INFORMATION handout (pp. 53-54). Use these scripted instructions to read through the handout with the volunteers. All scripted instructions to read aloud are italicized. To better understand why we are teaching these ideas, bolded text includes information not printed on the handout. It is important that practice partners understand their roles and responsibilities before leaving this time of preparation. Distribute assigned character cases along with the PRACTICE PARTNER INFORMATION handout.

WHAT IS ROADMAP?

Thank you for your willingness to serve as a practice partner in the ROADMAP curricular intervention.

> ***Research shows that many newly-diagnosed patients have limited prior medical knowledge of diabetes. The strongest predictor of how patients make sense of this diagnosis is their perception of how diabetes was explained to them.***

The goal of this intervention is to improve practice in your system, by preparing clinicians to talk to patients about a new diabetes or prediabetes diagnosis.

ROLES AND RESPONSIBILITIES

> ***Diabetes ROADMAP includes teaching sessions, role play, and clinical rehearsal followed by feedback session. As a practice partner, you will be working with the intervention team during the clinical rehearsal and feedback session.***

You will have two primary responsibilities:

- *Playing the role of a simulated patient in a clinical encounter during the clinical rehearsal*
- *Providing feedback to the learners during the feedback session*

The clinical rehearsal will simulate a typical patient encounter in a clinical room. This exercise gives learners an opportunity to practice what they have learned. The feedback session allows time for group discussion and will follow the encounter in a separate room.

> ***Clinical rehearsal allows practice of new knowledge and skills in a safe environment where mistakes have little impact. The contribution of our practice partners during this portion is a critical part of the ROADMAP curricular intervention.***

CLINICAL REHEARSAL

> ***Previous interviews with actual patients inform the character cases to provide a realistic picture of a future patient.***
>
> ***You have been assigned a character case and will be acting as one of these patients. The character case page that has been given to you includes important information to help you in the clinical rehearsal. Once we've finished going over the information sheet, you will have time to read your case and ask any questions. Please read it carefully to gain understanding about the patient you***

are representing. When it is time to begin the clinical rehearsal, each practice partner will be taken to separate clinical rooms to wait for the learner that is assigned to your case. At this point, it may feel very similar to an appointment you have experienced in the past as you wait in the room for the learner to knock on the door and come in.

For the purpose of this rehearsal, you are playing the role of a patient who is at an appointment to learn about the results of their latest lab work. Imagine that the learner in this appointment is who ordered the lab work, and you already have an established relationship.

It is possible that your own story may have similarities in some way to your assigned case. Any similarities are completely coincidental. This is not intended to be about your personal health. For the success of this exercise, it is important that you allow your assigned character case to guide you in your interaction with the learner. You may be asked questions that aren't specifically addressed in the character case. In these instances, you have the freedom to answer and discuss things that fit appropriately. Imagine, to the best of your ability, that you are the patient whose case you have been assigned. Ask questions as you believe this patient would, and give realistic responses to questions that are asked of you.

At the conclusion of the encounter, the learner will leave the room, and you will remain in the room and have a few minutes to fill out a PRACTICE PARTNER FEEDBACK FORM.

During the teaching session, the learners were introduced to four steps that should be included in the communication of a diagnosis: explain the diagnosis, explore patient perceptions, establish goals for the appointment, and elicit patient preferences. This form has questions that help us to know if the learner successfully integrated these four steps into the encounter and will help you recall information to aid in discussion during the feedback session.

Feel free to make notes at the bottom of the page to help you remember specific things that you think are important for the learner to know.

You may have more than one learner assigned to you. If this is the case, after filling out the feedback form, you will remain in the room and repeat the same encounter with a different learner.

The additional learner(s) will knock on the door and the encounter will begin just as it did before. All of the information for your character remains the same.

Remember that this isn't a continuation of the encounter you just experienced. You are starting over as the same patient who is at an appointment to learn about the results of your latest lab work.

Once again, the learner for this appointment is who ordered the lab work, so there is already an established relationship.

FEEDBACK SESSION

After you have had a few minutes to complete the final feedback form, you will be escorted to a separate room where you will have an opportunity to talk with the learner(s) you saw in the clinic room.

In this second room, there will be the learner(s) that you met with, two or three additional learners and the practice partner they were assigned, plus a facilitator. The facilitator will use

questions and prompts to help you share feedback with the learners. Although that may sound intimidating, please be assured that these learners are looking forward to hearing from you.

The information that you share provides valuable feedback from the perspective of the patient allowing the learner to hear where they may be able to improve and make changes in the future.

The facilitator will prompt discussion and monitor the time to allow the maximum amount of feedback in the time that is available.

Please keep in mind that you will be giving feedback on more than one learner and that there is another practice partner in the room who will also be giving feedback.

At the conclusion of the feedback session, give your completed feedback forms to the facilitator before leaving.

PRACTICE PARTNER TIPS

Here is a list of tips, suggestions and things for practice partners to remember to help them play the role of practice partner. Consider talking through these with the practice partners.

- Remember that the goal of this exercise is to help the learner put what they have learned into practice. The focus is on communication with patients and the delivery of a diabetes diagnosis. You are not being asked to make any medical assessments or evaluate the clinician's medical decision-making.
- You do not need to have any professional medical knowledge to be a practice partner. All the medical information that you need to know is contained within the character case.
- If you've had a previous encounter with the learner who is assigned to you, try not to let that influence how you interact.
- Do your best to "become" the character you have been assigned. Don't let information about your personal health interfere with the time you have with the learner.
- Stay on track with the reason that you are there, as detailed within your character case. Although it is entirely realistic that there are "rabbit trails" in a typical appointment, it is important to stay on topic for this learning activity.

Instructions for learners

Learners have not necessarily participated in clinical rehearsal. Similar to an objective structured clinical examination (OSCE), the approach in this environment is for teaching, not testing. This may be a new idea to some learners.

Take about two minutes to explain the clinical rehearsal and what its purpose is here. The following information serves as a guide to help you instruct your learners about what is coming next.

"Now we're going to apply these skills in a more clinically-similar environment. We have clinical rooms where you will practice with a simulated patient. These individuals are called "practice partners" because the encounters are not standardized for grading. We have provided character cases to them that are based on interviews with real patients living with prediabetes or type 2 diabetes. The patients are waiting in the rooms just as they would be in our clinic."

"Each patient is different, but they are all here to find out the results of routine lab work. Last time you saw this patient you ordered this lab work. Begin the encounter with assessment and move to the plan. I will hand you information about the patient you will see before you go in the clinical room. Please take as many notes on the front of the information sheet as you'd like throughout the encounter."

"You will have 15 minutes to work through the interaction. Use this opportunity to practice a patient encounter. When you are finished, we'll move on to the feedback session."

Clinical rehearsal feedback session guide

After the patient encounter, the practice partners will join you to provide verbal feedback to the learners. Please use the following prompts to structure that discussion and record notes here.

What overall feedback do the practice partners have for the learners?
Hopefully they'll have a section of what they did well and what needs improvement. This can be global feedback on nonverbal communication, such as eye contact, or professionalism, such as respect and listening.

How could the learner have engaged you better in the appointment?
If they need more prompts:
How could they include you in setting goals for the appointment?
How could the learner have done a better job checking how you understand the disease? How could the learner better help you make a plan for how to move forward?

ROADMAP

PRACTICE PARTNER INFORMATION

WHAT IS ROADMAP?

Thank you for your willingness to serve as a practice partner in the ROADMAP curricular intervention.

The goal of this intervention is to improve practice in your system, by preparing clinicians to talk to patients about a new diabetes or prediabetes diagnosis.

ROLES AND RESPONSIBILITIES

You will have two primary responsibilities:

- Playing the role of a simulated patient in a clinical encounter during the clinical rehearsal
- Providing feedback to the learners during the feedback session

The clinical rehearsal will simulate a typical patient encounter in a clinical room. This exercise gives learners an opportunity to practice what they have learned. The feedback session allows time for group discussion and will follow the encounter in a separate room.

CLINICAL REHEARSAL

For the purpose of this rehearsal, you are playing the role of a patient who is at an appointment to learn about the results of their latest lab work. Imagine that the learner in this appointment is who ordered the lab work, and you already have an established relationship.

It is possible that your own story may have similarities in some way to your assigned case. Any similarities are completely coincidental. This is not intended to be about your personal health. For the success of this exercise, it is important that you allow your assigned character case to guide you in your interaction with the learner. You may be asked questions that aren't specifically addressed in the character case. In these instances, you have the freedom to answer and discuss things that fit appropriately. Imagine, to the best of your ability, that you are the patient whose case you have been assigned. Ask questions as you believe this patient would, and give realistic responses to questions that are asked of you.

At the conclusion of the encounter, the learner will leave the room, and you will remain in the room and have a few minutes to fill out a PRACTICE PARTNER FEEDBACK FORM.

You may have more than one learner assigned to you. If this is the case, after filling out the feedback form, you will remain in the room and repeat the same encounter with a different learner.

Remember that this isn't a continuation of the encounter you just experienced. You are starting over as the same patient who is at an appointment to learn about the results of your latest lab work.

FEEDBACK SESSION

After you have had a few minutes to complete the final feedback form, you will be escorted to a separate room where you will have an opportunity to talk with the learner(s) you saw in the clinic room.

The information that you share provides valuable feedback from the perspective of the patient allowing the learner to hear where they may be able to improve and make changes in the future.

Please keep in mind that you will be giving feedback on more than one learner and that there is another practice partner in the room who will also be giving feedback.

At the conclusion of the feedback session, give your completed feedback forms to the facilitator before leaving.

PRACTICE PARTNER TIPS

Here is a list of tips, suggestions and things for practice partners to remember to help them play the role of practice partner. Consider talking through these with the practice partners.

- Remember that the goal of this exercise is to help the learner put what they have learned into practice. The focus is on communication with patients and the delivery of a diabetes diagnosis. You are not being asked to make any medical assessments or evaluate the clinician's medical decision-making.
- You do not need to have any professional medical knowledge to be a practice partner. All the medical information that you need to know is contained within the character case.
- If you've had a previous encounter with the learner who is assigned to you, try not to let that influence how you interact.
- Do your best to "become" the character you have been assigned. Don't let information about your personal health interfere with the time you have with the learner.
- Stay on track with the reason that you are there, as detailed within your character case. Although it is entirely realistic that there are "rabbit trails" in a typical appointment, it is important to stay on topic for this learning activity.

PRACTICE PARTNER FEEDBACK FORM

Learner name:		
Did the learner explain the new diagnosis?	No	Yes
Did the learner talk to you about how serious the condition is?	No	Yes
Did the learner ask what you wanted to accomplish in the appointment today?	No	Yes
Did the learner ask you about what would prevent you from making changes appropriate to this diagnosis?	No	Yes
Did the learner help you create a plan to address barriers to healthy eating and exercise?	No	Yes
What other feedback would you like to share with the learner?		

Character case set

Each case includes a character summary for the practice partner along with information sheets for the learner.

DIAGNOSIS: PREDIABETES

George Cuevas, 51-year-old Hispanic male ____ 57

Phyllis Black, 51-year-old white, non-Hispanic female ____ 59

Ken Black, 51-year-old white, non-Hispanic male ____ 62

Beulah White, 51-year-old African American female ____ 64

Albert White, 51-year-old African American male ____ 66

Angelica Bautista, 51-year-old Asian female ____ 68

DIAGNOSIS: TYPE 2 DIABETES

Jaime Cuevas, 51-year-old Hispanic male ____ 70

Felicia Black, 51-year-old white, non-Hispanic female ____ 72

Kevin Black, 51-year-old white, non-Hispanic male ____ 75

Beverly White, 51-year-old African American female ____ 77

Alvin White, 51-year-old African American male ____ 79

Angelina Bautista, 51-year-old Asian female ____ 82

MPCRN
MILITARY PRIMARY CARE RESEARCH NETWORK

Character cases written by Heather Rider, Angela Seehusen, Jasmyne Womack, and Christy Ledford.

Mr. George Cuevas is a 51 y/o Hispanic male who is here to review lab results.

**Today's appointment**	The labs were ordered as part of a routine physical. He has no reason to believe that anything is wrong. He does feel a little more tired which he believes is because of his schedule. The doctor will diagnose him with prediabetes. George's mother has diabetes so he has some working knowledge of the disease but is unfamiliar with the term "prediabetes."
**Characteristics**	George is an easy-going person with a very agreeable disposition. He is in a great mood today after recently finding out he is receiving a promotion at work.
**Medication**	George is on medication for high blood pressure.
**Background**	George grew up in a rural community in the west. His father was a miner. Being one of 5 boys, money was sometimes tight, but with staples like homemade flour tortillas, rice and beans, there was plenty to eat.<br>George was very active growing up and exercised regularly during his military service. In addition, he played softball and basketball with others from the unit. His military record outlines an incredible career filled with special duty assignments, many of them physically demanding.<br>He retired 5 years ago and since has become somewhat sedentary, other than a demanding job. His current job has him in the car much of the day, visiting the different sites that he manages. George's mother was diagnosed with type 2 diabetes 20 years ago. He has seen first-hand what happens when she doesn't eat or when she eats too many sweets.
**Smoking history**	Non-smoker
**Activity level**	He is somewhat sedentary, other than a demanding job. He does get out to walk the dogs, but one of the dogs is older and overweight and can only make it two blocks before he has to turn around and head home.
**Current diet**	Lunch typically consists of running to the drive thru and eating in the car on his way to the next location because of the convenience. Dinner is at home with his wife and mother-in-law. He enjoys his beer and admits to over-eating. His mother-in-law is also Hispanic and loves to cook just like her mother did. To her, the amount of food left on your plate at the end of a meal is a reflection of how much you liked it.
**Reaction to diagnosis**	George is unfamiliar with the term "prediabetes," but is concerned because he knows it has something to do with diabetes. He has watched his mother struggle with the disease. He is a man of action and willing to try anything, but he likes to see results and tends to stop things that don't produce them.

Patient information for physician

**Name**	George Cuevas
**Age**	51
**Reason for visit**	Follow-up labs
**Most recent lab results**	A1c: 5.8<br>Chem7 140/ 4.0 | 110/ 22| 18/0.9 < 135<br>Lipid panel TC: 195, HDL: 43; LDL: 97<br>TSH 2.78
**Vital signs today**	BP: 131/ 88<br>BMI: 29
**Medical history**	Hypertension
**Medication list**	Cinopril (Lisinopril) 10 mg<br>Ibuprofen PRN
**Technician review**	Patient up to date for age-based immunizations and preventive services

Encounter notes

Mrs. Phyllis Black is a 51 y/o white, non-Hispanic female who is here to review lab results.

**Today's appointment**	The labs were ordered as part of a routine physical. She feels tired most of the time, but is not experiencing any other symptoms. The doctor will diagnose her with prediabetes. Phyllis' mother had type 2 diabetes so she knows about diabetes but has not heard the term prediabetes.
**Characteristics**	Phyllis appears tired and somewhat anxious/stressed. Her shoulders are rounded and occasionally wrings her hands.
**Medication**	She is currently on medication for high blood pressure.
**Background**	Phyllis Black grew up with two sisters and a brother in the mid-west in a lower-middle class family. The family was never hungry, but frequently drank powdered milk and processed "convenience" foods that were cheap. They had fresh vegetables from their garden, but that was seasonal. She would eat fried chicken and mashed potatoes for Sunday dinner if they went to her grandparents' house after church. There would also be cookies and pie which she always enjoyed and equated with her grandmother's love. Her grandmother had diabetes, and Phyllis remembers her having to inject insulin, but she doesn't remember her grandmother ever complaining about it. Her grandmother died suddenly – she thinks from something cardiovascular related, but she doesn't know for sure.<br><br>Phyllis didn't learn to cook growing up, but she married a Southern man who loves biscuits and gravy and fried pork chops. She learned this Southern style of cooking from her mother-in-law and has been eating this way most of her adult life. She loves her husband, Don, and they have two grown children who are out of the house and a grandchild on the way. Phyllis has been thinking about their eating habits and knows it isn't good for their health, but doesn't have the energy to do anything about it right now. Don is supportive of Phyllis, however, he may be resistant to any changes in his diet.<br><br>Phyllis' mother developed type 2 diabetes in her 60s. She saw her mother eating cookies and pie even after her diagnosis, which was upsetting. Her mother was on oral medication for diabetes as well as hypertension and hyperlipidemia. Her mother died suddenly, a year ago, at the age of 73 from a stroke. Phyllis lost one of her sisters to breast cancer about 4 months ago. She has been trying to take care of her father and her sisters' family, but it has taken a toll on her health. She has gained 20 pounds and has not been feeling like walking or swimming. She is generally fatigued.
**Smoking history**	Smoked a few cigarettes/day while in college, but quit after graduating
**Activity level**	Phyllis used to walk her dog 20 minutes 3x/week, swim in the summer, and regular housekeeping, but doesn't feel like doing much since her recent loss.
**Current diet**	Phyllis didn't learn to cook growing up, but she married a Southern man who loves biscuits and gravy and fried pork chops. Phyllis has been thinking about their eating

	habits and knows it isn't good for their health, but doesn't have the energy to do anything about it right now.<br><br>She has recently been drinking her favorite mint juleps a little more than usual.
**Reaction to diagnosis**	Phyllis is confused by the term prediabetes, but is not surprised to hear "diabetes" and that she has abnormal lab results. She knows she has "let herself go" since the loss of her mother and sister. She is saddened and burdened to hear *another* piece of bad news; however, this is a wake-up call. Phyllis knows she needs to take care of herself because she wants to be around for her husband, children, and grandchildren.

Patient information for physician	
**Name**	Phyllis Black
**Age**	51
**Reason for visit**	Follow-up labs
**Most recent lab results**	A1c: 5.8<br>Chem7 140/ 4.0 | 110/ 22| 18/0.9 < 135<br>Lipid panel TC: 195, HDL: 43; LDL: 97<br>TSH 2.78
**Vital signs today**	BP: 131/ 88<br>BMI: 29
**Medical history**	Hypertension
**Medication list**	Cinopril (Lisinopril) 10 mg<br>Ibuprofen PRN
**Technician review**	Patient up to date for age-based immunizations and preventive services

<u>Encounter notes</u>

Mr. Ken Black is a 51 y/o white, non-Hispanic male who is here to review lab results.

**Today's appointment**	The labs were ordered as part of a routine physical. The doctor will diagnose him with prediabetes.
**Characteristics**	Ken has had very positive past encounters with the staff. He is personable, forthcoming and direct about his background. He isn't a rambler.
**Medication**	He is currently on medication for high blood pressure.
**Background**	Growing up in a small town, Ken didn't eat many vegetables or fruits. Meat was a luxury at times so he ate mostly carbohydrates and didn't begin eating more protein until after a few years in foster homes. Once he entered the military and had a lot more food available to him, he began to eat more vegetables and fruits.<br>Ken had to lose a couple of pounds to join the military and drastically lost weight while in basic training. Despite this, he's always been active and was much more so once in the service by playing basketball, swimming, and running. Once he stopped doing physical training every day, his weight ballooned. He rolled his ankle while out hiking and also twisted his knee while at work, so now he feels a lot of swelling if he's on his feet too long.<br>His mom has diabetes.
**Smoking history**	None
**Activity level**	He tries to walk two miles a day with his wife. He used to bike more, but it hurts his knee.
**Current diet**	Ken's diet now consists of generally a lot of food he considers unhealthy. He's been "eating out of control." He enjoys a couple of beers on the weekends.
**Reaction to diagnosis**	His initial reaction is shock. He isn't sure what prediabetes is, but he knows it's something you don't want to be involved with. Whatever the physician says, he'll say "okay, fine." Despite how many questions he has, he won't ask them here, but will look them up on the Internet at home. He wants as much information as the physician can give and he's very interested in any classes the physician may recommend.<br>His motivation is to not be diagnosed with type 2 diabetes and have "all the side effects and stuff." He's nervous about the "lifestyle" changes he may have to make such as having to take medication, giving himself insulin shots, and having to abstain from alcohol. He's already on one pill and "doesn't need another."<br>If the physician asks about behavioral goal, he's interested in how losing weight and eating better can keep his A1c levels low.

Patient information for physician	
**Name**	Ken Black
**Age**	51
**Reason for visit**	Follow-up labs
**Most recent lab results**	A1c: 5.8<br>Chem7 140/ 4.0 | 110/ 22| 18/0.9 < 135<br>Lipid panel TC: 195, HDL: 43; LDL: 97<br>TSH 2.78
**Vital signs today**	BP: 131/ 88<br>BMI: 29
**Medical history**	Hypertension
**Medication list**	Cinopril (Lisinopril) 10 mg<br>Ibuprofen PRN
**Technician review**	Patient up to date for age-based immunizations and preventive services

Encounter notes

Mrs. Beulah White is a 51 y/o African American female who is here to review lab results.

**Today's appointment**	The labs were ordered as part of a routine physical. The doctor will diagnose her with prediabetes.
**Characteristics**	Beulah is reserved. She's highly intelligent and is a cautious listener. She wants to hear what you have to say and then thinks about it before she responds.
**Medication**	She has high blood pressure and is on medication for it. She is not experiencing any type of symptoms.
**Background**	Beulah grew up in Georgia. She is married and has one grown son. She still home cooks for her family, particularly chicken and seafood. Her husband and kids all like dessert so she bakes for them, especially on the holidays – red velvet cake, carrot cake, and sweet potato pie.<br>Beulah's mother has prediabetes, and her father has "full blown diabetes" and takes medication twice a day. As a child, she remembers her great grandmother had diabetes; her family said, "she had sugar so she's gotta watch what she eats."<br>Beulah is a home care specialist. She spends her days with patients who have chronic medical conditions. She has taken care of patients who are on dialysis so she understands the potential long-term, scary consequences of diabetes.
**Smoking history**	None
**Activity level**	Beulah has a mentally and physically challenging job so she doesn't exercise for exercise's sake. She does yard work and is outside when it's nice.
**Current diet**	Beulah eats mostly her own cooking at home. They eat a lot of chicken and seafood. She shops at the grocery store but prefers fresh produce stands in the summertime. Beulah enjoys pasta, and bread, and sweets. When she goes out to eat, she really likes chips and salsa. She loves cranberry juice.
**Reaction to diagnosis**	Beulah is not surprised when the doctor tells her about prediabetes because of her family history. She expects she can "take care" of prediabetes with her diet and exercise. She doesn't ever want to have to be on medication for it.

**Patient information for physician**	
**Name**	Beulah White
**Age**	51
**Reason for visit**	Follow-up labs
**Most recent lab results**	A1c: 5.8<br>Chem7 140/ 4.0 | 110/ 22| 18/0.9 < 135<br>Lipid panel TC: 195, HDL: 43; LDL: 97<br>TSH 2.78
**Vital signs today**	BP: 131/ 88<br>BMI: 29
**Medical history**	Hypertension
**Medication list**	Cinopril (Lisinopril) 10 mg<br>Ibuprofen PRN
**Technician review**	Patient up to date for age-based immunizations and preventive services

Encounter notes

Mr. Albert White is a 51 y/o African American male who is here to review lab results.

**Today's appointment**	The labs were ordered as part of a routine physical. The doctor will diagnose him with prediabetes. Albert's mother and aunt had type 1 diabetes, and he knows people at church with type 2 diabetes, so he has some knowledge of the disease but is unfamiliar with the term "prediabetes."
**Characteristics**	Albert is an easy-going man who enjoys talking with people. He is generally an upbeat person who places his worry in God's hands. Although Albert has had many struggles in his life, he feels like he has persevered when he was true to his faith. When facing new challenges, he may need some time to let news sink in, but will then take them on, doing whatever he needs to overcome.
**Medication**	He is currently on medication for high blood pressure. He would rather not take medications but is compliant.
**Background**	Albert currently lives with his wife of 31 years. They enjoy going to church and having their two grown daughters and grandchildren over for Sunday dinners, like he had growing up. Over the last year or so has gained about 30 pounds.
**Smoking history**	Albert smoked for a few years after high school, but quit after meeting his wife.
**Activity level**	Albert has become mostly sedentary since taking the truck driving job. He continues to lift some weights on his days off and plays a little basketball with his grandsons on the weekend.
**Current diet**	Albert continues to love his wife's cooking, and he takes left-overs to work for lunch. A typical meal may be collards, beans and corn bread. He likes cereal in the mornings, but says the "healthy" cereals are expensive.<br><br>Albert may drink one or two beers during the week, but enjoys having a few on the weekend, especially if watching sports on television.
**Reaction to diagnosis**	Albert is scared to hear the word "diabetes," because he remembers what his mom and aunt went through, but he is confused by the term "prediabetes."<br><br>If the diagnosis is explained, Albert will be less fearful and will want to know what he can do to "fix" it. If given the option, he would rather try to make lifestyle changes than take medicine. He will go along with taking oral medication if it means he won't have to stick himself with needles.<br><br>If prediabetes is not explained, he will continue to be fearful and have questions about what it is and if that means he is "doomed." He will want to know if he is definitely going to get diabetes and if there's anything he can do to stop it. If given enough information, he will approach this challenge with help of the Holy Spirit and a "can do" attitude.

<table>
<tr><th colspan="2">Patient information for physician</th></tr>
<tr><td>Name</td><td>Albert White</td></tr>
<tr><td>Age</td><td>51</td></tr>
<tr><td>Reason for visit</td><td>Follow-up labs</td></tr>
<tr><td>Most recent lab results</td><td>A1c: 5.8
Chem7 140/ 4.0 | 110/ 22| 18/0.9 < 135
Lipid panel TC: 195, HDL: 43; LDL: 97
TSH 2.78</td></tr>
<tr><td>Vital signs today</td><td>BP: 131/ 88
BMI: 29</td></tr>
<tr><td>Medical history</td><td>Hypertension</td></tr>
<tr><td>Medication list</td><td>Cinopril (Lisinopril) 10 mg
Ibuprofen PRN</td></tr>
<tr><td>Technician review</td><td>Patient up to date for age-based immunizations and preventive services</td></tr>
</table>

Encounter notes

Mrs. Angelica Bautista is a 51 y/o Asian female who is here to review lab results.

**Today's appointment**	The labs were ordered as part of a routine physical. The doctor will diagnose her with prediabetes.
**Characteristics**	Angelica is very pleasant and usually smiling.
**Medication**	She is currently on medication for high blood pressure.
**Background**	Angelica grew up in the Philippines. Her family was poor and, with six siblings, food was scarce. Every meal included rice. When available, they had vegetables from their garden, sometimes they added fish to their meal. Her father passed away when she was a teenager; she thinks it was something related to his heart, she is unsure of the actual cause.<br><br>Angelica met and married her husband while he was stationed in the Philippines. Moving to the United States was a culture shock. The abundance and availability of resources, especially when going to the grocery store, was a bit overwhelming. She quickly adapted and learned to cook new things, but rice has been and will continue to be a part of her daily diet. She has a successful clerical career.<br><br>Her husband passed away from heart disease three years ago. About a year after his passing, she recognized that she was "eating more and getting fat." She became intentional with exercise and has become active in the local community center.<br><br>Angelica's mother, who lives in the same town, was diagnosed with type 2 diabetes five years ago. Because of this, they tried to switch to brown rice but found that they just didn't like it. For the most part, her mother has managed her diabetes well and doesn't seem to have any problems "as long as she takes her pills." Angelica has never heard of prediabetes.
**Smoking history**	Non-smoker
**Activity level**	Very active. She walks daily and is consistent with exercise classes five days each week at her gym.
**Current diet**	She feels that she eats fairly healthy, always incorporating vegetables with dinner. She doesn't eat much sugar, although she enjoys ice cream from time to time. She eats rice with every meal. She enjoys drinking an occasional glass of wine.
**Reaction to diagnosis**	Initially, Angelica doesn't understand the doctor's term "prediabetes." She only hears "diabetes" and recognizes that as to why her mother had to start taking pills five years ago. She isn't sure why she has it but is insistent (with a smile) that she won't stop eating rice. She is terrified of needles and doesn't like the idea of having to check her blood sugar like her mother does.

**Patient information for physician**	
**Name**	Angelica Bautista
**Age**	51
**Reason for visit**	Follow-up labs
**Most recent lab results**	A1c: 5.8<br>Chem7 140/ 4.0 | 110/ 22| 18/0.9 < 135<br>Lipid panel TC: 195, HDL: 43; LDL: 97<br>TSH 2.78
**Vital signs today**	BP: 131/ 88<br>BMI: 26
**Medical history**	Hypertension
**Medication list**	Cinopril (Lisinopril) 10 mg<br>Ibuprofen PRN
**Technician review**	Patient up to date for age-based immunizations and preventive services

<u>Encounter notes</u>

Mr. Jaime Cuevas is a 51 y/o Hispanic male who is here to review lab results.

**Today's appointment**	The labs were ordered as part of a routine physical. He has no reason to believe that anything is wrong. He does feel a little more tired which he believes is because of his schedule. The doctor will diagnose him with diabetes. Jaime's mother has diabetes so he has some working knowledge of the disease.
**Characteristics**	Jaime is an easy-going person with an agreeable disposition. He is in a great mood today after recently finding out he is receiving a promotion at work.
**Medication**	Jaime is currently on medication for high blood pressure.
**Background**	Jaime grew up in a rural community in the west. His father was a miner. Being one of 5 boys, money was sometimes tight, but with staples like homemade flour tortillas, rice and beans, there was plenty to eat.<br><br>Jaime was very active growing up and exercised regularly during his military service. In addition, he played softball and basketball with others from the unit. His military record outlines an incredible career filled with special duty assignments, many of them physically demanding.<br><br>He retired 5 years ago and since has become somewhat sedentary, other than a demanding job. His current job has him in the car much of the day, visiting the different sites that he manages.<br><br>Jaime's mother was diagnosed with type 2 diabetes 20 years ago. He has seen first-hand what happens when she doesn't eat or when she eats too many sweets. His father passed away when Jaime was 18 from lung cancer.
**Smoking history**	Non-smoker
**Activity level**	He is somewhat sedentary, other than a demanding job. He does get out to walk the dogs, but one of the dogs is older and overweight and can only make it two blocks before he has to turn around and head home.
**Current diet**	Lunch typically consists of running to the drive thru and eating in the car on his way to the next location because of the convenience. Dinner is at home with his wife and mother-in-law. He enjoys his beer and admits to over-eating. He's a grandpa who loves his grandchildren. His mother-in-law is also Hispanic and loves to cook just like her mother did. To her, the amount of food left on your plate at the end of a meal is a reflection of how much you liked it.
**Reaction to diagnosis**	Jaime is concerned because he has watched his mother struggle with the disease. He is a man of action and willing to try anything but he likes to see results and tends to stop things that don't produce them.

Patient information for physician	
**Name**	Jaime Cuevas
**Age**	51
**Reason for visit**	Follow-up labs
**Most recent lab results**	A1c: 7.1<br>Chem7 140/ 4.0 | 110/ 22| 18/0.9 < 135<br>Lipid panel TC: 195, HDL: 43; LDL: 97<br>TSH 2.78
**Vital signs today**	BP: 131/ 88<br>BMI: 29
**Medical history**	Hypertension
**Medication list**	Cinopril (Lisinopril) 10 mg<br>Ibuprofen PRN
**Technician review**	Patient up to date for age-based immunizations and preventive services

Encounter notes

Mrs. Felicia Black is a 51 y/o white, non-Hispanic female who is here to review lab results.

**Today's appointment**	The labs were ordered as part of a routine physical. She feels tired most of the time, but is not experiencing any other symptoms. The doctor will diagnose her with diabetes. Felicia's mother developed diabetes in her 60s so she knows about diabetes.
**Characteristics**	Felicia appears tired and somewhat anxious/stressed. Her shoulders are rounded and occasionally wrings her hands.
**Medication**	She is currently on medication for high blood pressure.
**Background**	Felicia Black grew up with two sisters and a brother in the mid-west in a lower-middle class family. The family was never hungry, but frequently drank powdered milk and processed "convenience" foods that were cheap. They had fresh vegetables in the summer from their garden, but that was seasonal. She would eat fried chicken and mashed potatoes for Sunday dinner if they went to her grandparents' house after church. There would also be cookies and pie which she always enjoyed and equated with her grandmother's love. Her grandmother had type 2 diabetes and Felicia remembers her having to inject insulin, but she doesn't remember her grandmother ever complaining about it. Her grandmother died suddenly; she thinks from something cardiovascular related, but she was young and doesn't know for sure.<br><br>She loves her husband, Don, and they have two grown children who are out of the house and a grandchild on the way. Felicia has been thinking about their eating habits and knows it isn't good for their health, but doesn't have the energy to do anything about it right now. Her husband is overweight, but really doesn't have any health problems. Don is supportive of Felicia, however, he may be resistant to any changes in his diet.<br><br>Felicia' mother developed type 2 diabetes in her 60s. She saw her mother eating cookies and pie even after her diagnosis, which was upsetting. Her mother was on oral medication for diabetes as well as hypertension and hyperlipidemia. Her mother died suddenly, a year ago, at the age of 73 from a stroke. Felicia lost one of her sisters to breast cancer about 4 months ago. She has been trying to take care of her father and her sisters' family, but it has taken a toll on her health. She has gained 20 pounds and has not been feeling like walking or swimming. She is generally fatigued.
**Smoking history**	Smoked a few cigarettes per day while in college, but quit after graduating
**Activity level**	Felicia used to walk her dog 20 minutes each day, swim in the summer, and regular housekeeping, but doesn't feel like doing much since her recent loss.
**Current diet**	Felicia didn't learn to cook growing up, but she married a Southern man who loves biscuits and gravy and fried pork chops. Felicia has been thinking about their eating habits and knows it isn't good for their health, but doesn't have the energy to do

	anything about it right now. She has recently been drinking her favorite mint juleps a little more than usual.
**Reaction to diagnosis**	Felicia is not surprised to hear "diabetes" and that she has abnormal lab results. She knows she has "let herself go" since the loss of her mother and sister. She is saddened and burdened to hear *another* piece of bad news; however, this is a wake-up call. Felicia knows she needs to take care of herself because she wants to be around for her husband, children, and grandchildren.

**Patient information for physician**	
**Name**	Felicia Black
**Age**	51
**Reason for visit**	Follow-up labs
**Most recent lab results**	A1c: 7.1<br>Chem7 140/ 4.0 | 110/ 22| 18/0.9 < 135<br>Lipid panel TC: 195, HDL: 43; LDL: 97<br>TSH 2.78
**Vital signs today**	BP: 131/ 88<br>BMI: 29
**Medical history**	Hypertension
**Medication list**	Cinopril (Lisinopril) 10 mg<br>Ibuprofen PRN
**Technician review**	Patient up to date for age-based immunizations and preventive services

Encounter notes

Mr. Kevin Black is a 51 y/o white, non-Hispanic male who is here to review lab results.

**Today's appointment**	The labs were ordered as part of a routine physical. The doctor will diagnose him with diabetes.
**Characteristics**	Kevin has had very positive past encounters with the staff. He is personable, forthcoming and direct about his background. He isn't a rambler.
**Medication**	He is currently on medication for high blood pressure.
**Background**	Growing up in a small town, Kevin didn't eat many vegetables or fruits. Meat was a luxury at times so he ate mostly carbohydrates and didn't begin eating more protein until after a few years in foster homes. After he entered the military and ate at dining halls, he began to include more vegetables and fruits into his diet.<br><br>Kevin had to lose a couple of pounds to join the military and drastically lost weight while in basic training. Despite this, he's always been active and was much more so once in the service by playing basketball, swimming, and running. Once he stopped doing physical training every day, his weight ballooned. Kevin rolled his ankle while out hiking and twisted his knee while at work, so now he feels a lot of swelling if he's on his feet too long. He's also noticed an increase in urination, thirst, and sweating, even when it's cold.<br><br>His mom has diabetes.
**Smoking history**	None
**Activity level**	Kevin tries to walk two miles a day with his wife. He used to bike more, but it hurts his knee.
**Current diet**	Now his diet consists of generally a lot of food he considers unhealthy. He's been "eating out of control." He likes to enjoy a couple of beers on the weekends.
**Reaction to diagnosis**	Kevin's initial reaction is worry because he's heard of people needing to take "insulin shots." He's scared of his "body failing before its time" but now the past year of symptoms all make sense. He doesn't know much about diabetes except that "it's a blood sugar…like not eating healthy or whatever" and he wants nothing to do with it. He doesn't want to be that person who's "seen as diabetic."<br><br>He's nervous about having to give up the things he really enjoys, but he's willing to make lifestyle modifications. He's already on one pill and "doesn't need another." If the physician asks about behavioral goals, he'd like to know how losing weight will affect his diabetes. He wants to know how he can live healthier, move more, and eat better.

**Patient information for physician**	
**Name**	Kevin Black
**Age**	51
**Reason for visit**	Follow-up labs
**Most recent lab results**	A1c: 7.1<br>Chem7 140/ 4.0 | 110/ 22| 18/0.9 < 135<br>Lipid panel TC: 195, HDL: 43; LDL: 97<br>TSH 2.78
**Vital signs today**	BP: 131/ 88<br>BMI: 29
**Medical history**	Hypertension
**Medication list**	Cinopril (Lisinopril) 10 mg<br>Ibuprofen PRN
**Technician review**	Patient up to date for age-based immunizations and preventive services

<u>Encounter notes</u>

Mrs. Beverly White is a 51 y/o African American female who is here to review lab results.

**Today's appointment**	The labs were ordered as part of a routine physical. The provider today will diagnose Beverly with diabetes.
**Characteristics**	Beverly is a strong, independent woman. She is respectful but you can see her spirit. However, this appointment is emotional for her.
**Medication**	She has high blood pressure and takes medicine for it, but she feels fine.
**Background**	Beverly is married to a Navy man who was a cook so he does the cooking and she does the cleaning. She has two grown daughters who live away, but no grandchildren. Beverly is originally from North Carolina. When she goes back to visit family, "Southern people love to feed you." No one in her family has diabetes.<br><br>Beverly is a numbers person. She likes to know where she stands. She is retired from the military.
**Smoking history**	None
**Activity level**	When she was in the military, she was doing physical training five days a week, but since she's retired, she doesn't have an exercise routine.
**Current diet**	Beverly likes to have a Krispy Kreme donut in the morning with her coffee. She grew up eating a lot of rice in North Carolina and still does. She also loves corn. She drinks sweet tea but doesn't really like soda. Her favorite dessert is ice cream. She likes to eat at S&S cafeteria because they have a lot of choices, but she also likes a McDonald's hamburger.<br><br>For Beverly, a meal isn't a meal without bread.
**Reaction to diagnosis**	Beverly is shocked. She doesn't even know what questions to ask. She is anxious and fearful and just can't understand it since no one in her family has had diabetes. Someone in her church has had his foot amputated and that scares her. She is hurt; she is scared; she is depressed; she is worried.

**Patient information for physician**	
**Name**	Beverly White
**Age**	51
**Reason for visit**	Follow-up labs
**Most recent lab results**	A1c: 7.1<br>Chem7 140/ 4.0 | 110/ 22| 18/0.9 < 135<br>Lipid panel TC: 195, HDL: 43; LDL: 97<br>TSH 2.78
**Vital signs today**	BP: 131/ 88<br>BMI: 29
**Medical history**	Hypertension
**Medication list**	Cinopril (Lisinopril) 10 mg<br>Ibuprofen PRN
**Technician review**	Patient up to date for age-based immunizations and preventive services

Encounter notes

Mr. Alvin White is a 51 y/o African American male who is here to review lab results.

**Today's appointment**	The labs were ordered as part of a routine physical. The doctor will diagnose him with diabetes. Alvin's mother and aunt had type 1 diabetes, and he knows people at church with type 2 diabetes, so he has some knowledge of the disease.
**Characteristics**	Alvin is an easy-going man who enjoys talking with people. He is generally an upbeat person who places his worry in God's hands. Although Alvin has had many struggles in his life, he feels like he has persevered when he was true to his faith. When facing new challenges, he may need some time to let news sink in, but will then take it on, doing whatever he needs to overcome them.
**Medication**	He is currently on medication for high blood pressure. He would rather not take medications but is compliant.
**Background**	Alvin grew up in the "country" with his mom, dad, and three brothers. His family had a dairy farm and a garden, so he always had fresh vegetables and milk. After going to Sunday church, they would have a big meal with his extended family. Family meals included fried chicken, collard greens, cornbread, and his favorite, mashed potatoes. Alvin remembers hearing older people at church saying they got "the sugar" and their limbs "falling off." Alvin's mother and aunt had type 1 diabetes, and he vividly recalls them getting insulin from the refrigerator, drawing it up from the vial and injecting it with a syringe. After witnessing this, Alvin became fearful of needles. His mother died of complications from diabetes when he was 20.<br><br>Alvin is semi-retired from law enforcement, but he still works about 20-30 hours a week, driving a delivery truck. He used to feel "like a Ferrari," but since retiring, he "feels like his body is becoming a dump truck." Alvin lives with his wife of 31 years. They enjoy going to church and having their two grown daughters and grandchildren over for Sunday dinners, like he had growing up. Over the last year or so, he has gained about 30 pounds.
**Smoking history**	Alvin smoked after graduating from high school, but quit after meeting his wife.
**Activity level**	Alvin has become mostly sedentary since taking the truck driving job. He continues to lift some weights on his days off and plays a little basketball with his grandsons on the weekend, but swore he would never run again after he retired.
**Current diet**	Alvin continues to love his wife's cooking and he takes left-overs to work for lunch. A typical meal may be collards, beans, and corn bread. He likes cereal in the mornings, but says the "healthy" cereals are expensive.<br><br>Alvin may drink one or two beers during the week, but enjoys having a few on the weekend, especially if watching sports on television.

**Reaction to diagnosis**	Alvin is scared to hear the word "diabetes" and feels like he is "doomed." He will bring up images of older people at church with missing limbs and talking about "the sugar." Alvin will worry about what that means for the health of his girls.<br><br>After the initial fear, he will be sad to think about giving up his favorite foods. Alvin will have questions about exercise and if that means he needs to start running again. If given time and explanation, he will be receptive to advice. Alvin doesn't like having to take medication, but is willing to do whatever will prevent him from having to use needles or lose a limb. If given enough information, he will approach this challenge with help of the Holy Spirit and a "can do" attitude.

Patient information for physician	
**Name**	Alvin White
**Age**	51
**Reason for visit**	Follow-up labs
**Most recent lab results**	A1c: 7.1<br>Chem7 140/ 4.0 | 110/ 22| 18/0.9 < 135<br>Lipid panel TC: 195, HDL: 43; LDL: 97<br>TSH 2.78
**Vital signs today**	BP: 131/ 88<br>BMI: 29
**Medical history**	Hypertension
**Medication list**	Cinopril (Lisinopril) 10 mg<br>Ibuprofen PRN
**Technician review**	Patient up to date for age-based immunizations and preventive services

Encounter notes

Mrs. Angelina Bautista is a 51 y/o Asian female who is here to review lab results.

**Today's appointment**	The labs were ordered as part of a routine physical. The doctor will diagnose her with diabetes.
**Characteristics**	Angelina is very pleasant and usually smiling.
**Medication**	She is currently on medication for high blood pressure.
**Background**	Angelina grew up in the Philippines. Her family was poor, and with six siblings, food was scarce. Every meal included rice. When available, they had vegetables from their garden, sometimes they added fish to their meal. Her father passed away when she was a teenager; she thinks it was something related to his heart, she is unsure of the actual cause.<br><br>Angelina met and married her husband while he was stationed in the Philippines. Moving to the United States was a culture shock. The abundance and availability of resources, especially when going to the grocery store, was a bit overwhelming. She quickly adapted and learned to cook new things, but rice has been and will continue to be a part of her daily diet. She has a successful clerical career.<br><br>Her husband passed away from heart disease three years ago. About a year after his passing, she recognized that she was "eating more and getting fat." She became intentional with exercise and has become active in the local community center.<br><br>Angelina's mother, who lives in the same town, was diagnosed with type 2 diabetes five years ago. Because of this, they tried to switch to brown rice but found that they just didn't like it. For the most part, her mother has managed her diabetes well and doesn't seem to have any problems "as long as she takes her pills."
**Smoking history**	None
**Activity level**	Very active. She walks daily and is consistent with exercise classes five days each week at her gym.
**Current diet**	She feels that she eats fairly healthy, always incorporating vegetables with dinner. She doesn't eat much sugar, although she enjoys ice cream from time to time. She eats rice with every meal. She enjoys drinking an occasional glass of wine.
**Reaction to diagnosis**	Angelina hears "diabetes" and recognizes that as to why her mother had to start taking pills five years ago. She isn't sure why she has it but is insistent (with a smile) that she won't stop eating rice. She is terrified of needles and doesn't like the idea of having to check her blood sugar like her mother does.

**Patient information for physician**	
**Name**	Angelina Bautista
**Age**	51
**Reason for visit**	Follow-up labs
**Most recent lab results**	A1c: 7.1<br>Chem7 140/ 4.0 | 110/ 22| 18/0.9 < 135<br>Lipid panel TC: 195, HDL: 43; LDL: 97<br>TSH 2.78
**Vital signs today**	BP: 131/ 88<br>BMI: 27
**Medical history**	Hypertension
**Medication list**	Cinopril (Lisinopril) 10 mg<br>Ibuprofen PRN
**Technician review**	Patient up to date for age-based immunizations and preventive services

<u>Encounter notes</u>
