# Supplementary material for: Diabetes ROADMAP: Teaching Guideline Use, Communication, and Documentation When Delivering the Diagnosis of Diabetes
Source: MedEdPORTAL. 2020 Sep 11;16:10959. doi: 10.15766/mep_2374-8265.10959 (PMC7485911; doi:10.15766/mep_2374-8265.10959)
Supplement: Supplementary file 1 — Curriculum Overview.pdfTeaching Guide.pdfROADMAP Presentation.pptxFacilitator Guide.pdfSimulation Resources.pdfAssessment Tools.pdf [file mep_2374-8265.10959-s001.zip › F. Assessment Tools.pdf]

ROADMAP

ASSESSMENT TOOLS

Uniformed Services University of the Health Sciences

MPCRN
MILITARY PRIMARY CARE RESEARCH NETWORK

Authors include: Christy JW Ledford and Stephanie Fulleborn.

Did ROADMAP improve practice?

Built into ROADMAP are a variety of curricular evaluations, which map onto each learning objective.

Objective 1: Can learners list and describe the current ADA guidelines on screening, diagnosis, and treatment of prediabetes and type 2 diabetes?

The LEARNER KNOWLEDGE CHECK (p. 41) provides a snapshot of learners' ability to describe the screening and treatment guidelines. As written, the questions reflect the 2019 guidelines. The LEARNER KNOWLEDGE CHECK KEY (pp. 42-43) connects items to the objectives and related content from the teaching session. Rather than an assessment of individual learners, the knowledge check gives educators a program perspective of which curriculum objectives were met.

Objective 2: Can learners identify the relationship between the diagnosis moment and potential for patient behavior change?

In the CURRICULUM EVALUATION, we ask about two ideas that are commonly linked to education outcomes: response efficacy and self-efficacy.

To assess this concept,	this item	shows
Response efficacy	"How I tell a patient about a new diagnosis will influence the patient's behavior change"	a measure of how learners feel confident that they can follow through on the behaviors taught in the intervention.
Self-efficacy	"I am confident in my ability to counsel a patient through the prediabetes/diabetes diagnosis"	if the learner recognizes the connection between their communication and the patient's likelihood to change their behavior.

Objective 3: Can learners demonstrate how to establish shared meaning when communicating a new diabetes diagnosis?

CLINICAL PRACTICE APPLICATION (p. 28) provides learners a guide to talk through an active clinical encounter with a peer or a preceptor, applying the skills from ROADMAP. This page can also act as a tool to demonstrate learner application.

Objective 4: Can learners document a meaningful diabetes diagnosis in the patient health record?

CLINICAL DOCUMENTATION APPLICATION (p. 29) provides learners a guide to talk through an active clinical note with a peer or a preceptor, applying the skills from ROADMAP. In residencies, as faculty review and co-sign clinical documentation, they can look at how learners have improved their clinical documentation skills. If clinical resources are available, educators or learners can also conduct peer review of health records of newly diagnosed patients to see how learners are documenting diagnosis conversations.

Educators can also follow up with learners after the curricular intervention to give the learners an opportunity for self-assessment. Learners can self-assess how the new knowledge and skills have been necessary for their patients and how well they have integrated that learning into their care for these patients.

LEARNER KNOWLEDGE CHECK

1) Mrs. Lopez is a 42-year-old female with a past medical history of gestational diabetes (diagnosed in her most recent pregnancy, 7 years ago) and hypertension who presents to clinic for an annual exam. Her family history is significant only for insulin-dependent type 2 diabetes in her maternal grandmother. Per ADA guidelines, you consider screening for diabetes in this patient because of her:
   a. History of gestational diabetes
   b. Hypertension
   c. Family history of diabetes
   d. A and B
   e. All of the above

2) Mrs. Lopez's hemoglobin A1c results at 6.7%. You advise her that:
   a. She has diabetes and needs to come back for diabetes education.
   b. The lab needs to repeat the test on the blood sample.
   c. She needs to go back to the lab for fasting blood glucose testing.
   d. This is probably a lab error, since she has no symptoms of diabetes.

3) Mr. Jackson has recently been diagnosed with prediabetes. He asks you about treatment options, and you tell him:
   a. Intensive lifestyle modification with at least 7% weight loss is the most effective way to delay the onset of type 2 diabetes.
   b. Taking Metformin daily is the most effective way to delay the onset of type 2 diabetes.
   c. Bariatric surgery is the most effective way to delay the onset of type 2 diabetes, and he is eligible since his BMI is >30.
   d. There is no known method that effectively delays the onset of type 2 diabetes.

4) What patient perceptions can help you understand if Mr. Jackson is ready to consider changing his disease-related behavior?
   a. How he thinks about the physician-patient relationship and how he trusts the physician
   b. How adverse he is to taking medication and how much pain he is experiencing
   c. How severe he thinks prediabetes is and how susceptible he thinks he is to the condition and its potential complications
   d. How bad he perceives his current dietary behavior and how that affects his mood and ability to exercise

5) Which part of the patient story should you document in the assessment and plan section of a clinical note?
   a. Mrs. Jordan tells you her sister has diabetes.
   b. Mr. Jackson tells you he just retired from the military.
   c. Ms. Johnson has questions about how diabetes is related to hypertension.
   d. Mr. Jefferson doesn't want to take any medication that he can avoid.

Learner knowledge check KEY

Objective: To list and define the current ADA guidelines on screening and diagnosis of prediabetes and type 2 diabetes.

1) Mrs. Lopez is a 42-year-old female with a past medical history of gestational diabetes (diagnosed in her most recent pregnancy, 7 years ago) and hypertension who presents to clinic for an annual exam. Her family history is significant only for insulin-dependent type 2 diabetes in her maternal grandmother. Per ADA guidelines, you consider screening for diabetes in this patient because of her:
    a. History of gestational diabetes
    b. Hypertension
    c. Family history of diabetes
    d. A and B
    e. All of the above

    (Refer to ADA screening guidelines on teaching slide 7. The answer does not also include family history because Mrs Lopez's family history is a second-degree relative.)

2) Mrs. Lopez's hemoglobin A1c results at 6.7%. You advise her that:
    a. She has diabetes and needs to come back for diabetes education.
    b. The lab needs to repeat the test on the blood sample.
    c. She needs to go back to the lab for fasting blood glucose testing.
    d. This is probably a lab error, since she has no symptoms of diabetes.

    (Refer to ADA diagnostic criteria on teaching slide 5.)

Objective: To list and describe the treatment guidelines of prediabetes and type 2 diabetes.

3) Mr. Jackson has recently been diagnosed with prediabetes. He asks you about treatment options, and you tell him:
    a. Intensive lifestyle modification with at least 7% weight loss is the most effective way to delay the onset of type 2 diabetes.
    b. Taking Metformin daily is the most effective way to delay the onset of type 2 diabetes.
    c. Bariatric surgery is the most effective way to delay the onset of type 2 diabetes, and he is eligible since his BMI is >30.
    d. There is no known method that effectively delays the onset of type 2 diabetes.

    (Refer to treatment options for prediabetes on teaching slide 9.)

Objective: To identify the relationship between the diagnosis moment and potential for patient behavior change.

4) What patient perceptions can help you understand if Mr. Jackson is ready to consider changing his disease-related behavior?
    a. How he thinks about the physician-patient relationship and how he trusts the physician
    b. How adverse he is to taking medication and how much pain he is experiencing
    c. How severe he thinks prediabetes is and how susceptible he thinks he is to the condition and its potential complications
    d. How bad he perceives his current dietary behavior and how that affects his mood and ability to exercise

(Refer to shared meaning and the moment of diagnosis on teaching slide 16.)

Objective: To document a meaningful diabetes diagnosis in the patient health record.

5) Which part of the patient story should you document in the assessment and plan section of a clinical note?
    a. Mrs. Jordan tells you her sister has diabetes.
    b. Mr. Jackson tells you he just retired from the military.
    c. Ms. Johnson has questions about how diabetes is related to hypertension.
    d. Mr. Jefferson doesn't want to take any medication that he can avoid.

(Refer to what to include in the A/P section of the note on teaching slide 33. Preferences for medication taking (or not taking) are an important for the next clinician's medical decision making.)

ROADMAP

CURRICULUM EVALUATION

This activity was a worthwhile learning experience.	Agree strongly	Agree somewhat	Neither agree nor disagree	Disagree somewhat	Disagree strongly
The content was delivered at an appropriate level of understanding.	Agree strongly	Agree somewhat	Neither agree nor disagree	Disagree somewhat	Disagree strongly

What are the top three things you learned today?

1. ____

2. ____

3. ____

How I tell a patient about a new diagnosis will influence the patient's behavior change.	Agree strongly	Agree somewhat	Neither agree nor disagree	Disagree somewhat	Disagree strongly
I am confident in my ability to counsel a patient through the prediabetes/diabetes diagnosis.	Agree strongly	Agree somewhat	Neither agree nor disagree	Disagree somewhat	Disagree strongly

How will what you learned today change your practice over the next month?

____

____

____
