# Supplementary material for: Diabetes ROADMAP: Teaching Guideline Use, Communication, and Documentation When Delivering the Diagnosis of Diabetes
Source: MedEdPORTAL. 2020 Sep 11;16:10959. doi: 10.15766/mep_2374-8265.10959 (PMC7485911; doi:10.15766/mep_2374-8265.10959)
Supplement: Supplementary file 1 — Curriculum Overview.pdfTeaching Guide.pdfROADMAP Presentation.pptxFacilitator Guide.pdfSimulation Resources.pdfAssessment Tools.pdf [file mep_2374-8265.10959-s001.zip › _Educational_Summary_Report_10959.pdf]

MedEdPORTAL® | The AAMC Journal of Teaching and Learning Resources

Original Publication Open Access

Diabetes ROADMAP: Teaching Guideline Use, Communication, and Documentation When Delivering the Diagnosis of Diabetes

Christy J. W. Ledford, PhD, Dean A. Seehusen, MD, MPH, Lauren A. Cafferty, MA, Heather A. Rider, Tyler Rogers, MD, Stephanie Fulleborn, MD, Erik Clauson, DO, CAQSM, Christopher C. Ledford, MD, Steven Trigg, MD, Jeremy T. Jackson*, Paul F. Crawford, MD

*Corresponding author: jtjackson@hjf.org

Abstract

Introduction: Most interventions to date regarding breaking bad news focus on late-stage disease or disclosing a cancer diagnosis. Little attention has been given to delivery of chronic metabolic disease diagnoses such as prediabetes/type 2 diabetes. **Methods:** Informed by the American Diabetes Association standards of care and formative research conducted by our research team, we developed this curriculum through the six-step approach to curriculum development. The curriculum consists of a 2- or 3-hour intervention that teaches medical decision-making, interpersonal communication, and clinical documentation in the context of prediabetes and type 2 diabetes followed by role-play and clinical practice. **Results:** Across three cohorts, 53 clinicians completed the curriculum. Across the three iterations, learners rated the curricular intervention as worthwhile and delivered at an appropriate level. In a community hospital setting, learners scored significantly higher on a knowledge check than did a control group of six clinicians ($p < .001$). Learners in the community hospital also indicated high response efficacy and self-efficacy. At the academic medical center, simulated patients indicated high measures on the Diabetes Health Threat Communication Questionnaire. **Discussion:** The moment of diagnosis presents a key opportunity to affect patients' perceptions of the disease. This curriculum guides clinicians in making the most of diagnosis delivery. Pairing of qualitative, patient-centered research alongside the iterative curriculum design process allows the curriculum to be adaptable and scalable to multiple settings and learner types.

Keywords
Diabetes, Diabetes Mellitus, Prediabetes, Prediabetic State, Family Medicine, Internal Medicine, Primary Care, Editor's Choice

Educational Objectives

By the end of this activity, learners will be able to:

1. List and describe the current American Diabetes Association guidelines on screening, diagnosis, and treatment of prediabetes and type 2 diabetes.
2. Identify the relationship between the diagnosis moment and potential for patient behavior change.
3. Demonstrate how to establish shared meaning when communicating a new diabetes diagnosis.
4. Document a meaningful diabetes diagnosis in the patient health record.

Citation:
Ledford CJW, Seehusen DA, Cafferty LA, et al. Diabetes ROADMAP: teaching guideline use, communication, and documentation when delivering the diagnosis of diabetes. *MedEdPORTAL*. 2020;16:10959. https://doi.org/10.15766/mep_2374-8265.10959

Introduction

Breaking bad news is a teachable skill.[1] Research has shown that learner-centered, skill- and practice-based programs are the most effective means of improving clinician communication skills.[2] Most teaching interventions to date, however, have focused on disclosing cancer diagnoses and discussing prognosis at an early stage of the disease. Although successful interventions have been developed to improve clinician skills in giving bad news,[3,4] little attention has been given to the delivery of a diagnosis of prediabetes/diabetes or other chronic metabolic disease.

After numerous interviews with patients who had been diagnosed with prediabetes and type 2 diabetes in the medical system, our research team discovered a trend of patients who were unaware of the scope and severity of their disease.[5-7] This curriculum, Diabetes ROADMAP (Responding to the Opportunity to Adapt the Diagnosis to Motivate and Activate Patients), was developed as a method of sharing the results of our research with the clinicians responsible for the care of these patients. Diabetes

Copyright © 2020 Ledford et al. This is an open-access publication distributed under the terms of the Creative Commons Attribution license.

ROADMAP provides training for any clinician who may deliver a diabetes diagnosis and is intended to prepare clinicians for this challenge. The curriculum consists of a 2-hour intervention (3 hours when including simulation) that teaches medical decision-making, interpersonal communication, and clinical documentation in the context of prediabetes and diabetes followed by small-group role-play and, finally, clinical practice. Diabetes ROADMAP represents a unique contribution to existing resources, including (1) curricula that teach more broadly about the diagnosis and management of diabetes[8,9] and (2) curricula that teach learners how to deliver bad news[4] such as a cancer diagnosis[10] or a negative turn in an admitted hospital patient's condition.[11]

Diabetes ROADMAP aims to achieve enduring positive change by implementing a communication intervention at the clinician level. Within the cultural framework for health,[12] this curriculum targets individual-level change by teaching clinicians communication skills to interact with patients newly diagnosed with prediabetes/diabetes. The primary theoretical framework for this curriculum is a hybrid health belief model.[13] In this adaptation of the model, we accept that cultural factors influence a patient's understanding of disease, likelihood to act, enacted behavior change, and resulting diabetes outcomes. This influence is what necessitates the health care system to adopt culturally competent and tailored communication throughout prevention, diagnosis, and treatment. The focus of this curriculum is the cue to action for the patient, which is delivered by the clinician in the diagnosis communication. Through an intervention that improves clinicians' ability to communicate with patients in a patient-centered, cultural framework, they can leverage the moment of diagnosis to increase patient activation and impact patient understanding of diabetes, which can result in a greater likelihood of behavior change.

A clinician will be more effective in disclosing a diagnosis to a patient when confident that he or she is accurately following clinical guidelines. Especially for prediabetes, there is confusion among providers about this designation and its meaning.[7,14] American Diabetes Association guidelines[15] are widely accepted and can be briefly reviewed to give clinicians confidence in their ability to make a correct diagnosis.

Properly documenting both the diagnosis and discussion within the medical record is also covered in the curriculum. This documentation conveys to subsequent clinicians that this key conversation has taken place. The electronic medical record is the official means of documenting discussions with patients and is the standard way for providers to communicate through time to other clinicians who see the patient in the future. Consistently and clearly documenting what diagnosis has been made and what message has been provided to a patient then allows the care team to present a common message, reducing confusion on the patient's part. This also prevents the care team from giving mixed messages that can undermine a patient's confidence in his or her health care.[16]

The instructional design of the curriculum is based on adult learning theory[17] and on research documenting characteristics of programs effective in improving skills[18] and changing clinical practice.[19] The learner-centered approach includes promoting learners' participation in setting goals and priorities and evaluating progress; encouraging sharing of personal and professional experiences related to course content; fostering a supportive, nonjudgmental learning climate that promotes risk-taking; engaging learners in interactive experiences in small and large groups (such as role-play, group discussion, and one-on-one consultation); and attending to relationship-building between faculty and participants and among participants.[18,20]

Two widely used frameworks for teaching breaking bad news are SPIKES (setting up interview, assessing patient's perception, obtaining patient's invitation, giving knowledge and information, addressing the patient's emotions, and summary)[21] and an extension of SPIKES called COMFORT (communication, orientation, mindfulness, family, ongoing, reiterative messages, and team).[22] Using the data from the formative phases of study, we have adapted and tested these existing protocols for our study by revising the frameworks to specifically address cultural competence within the setting of manageable chronic disease and evaluating them using simulated patient cases[23-26] with dyadic (patient-clinician) scenarios.

The Diabetes ROADMAP curriculum's four educational objectives align with the Accreditation Council for Graduate Medical Education (ACGME) core competencies of Patient Care (PC) and Interpersonal and Communication Skills (C).[27] The objectives map onto the following family medicine subcompetencies:

- PC-2: care for patients with chronic conditions.
- C-1: develop meaningful, therapeutic relationships with patients and families.
- C-2: communicate effectively with patients, families, and the public.
- C-3: develop relationships and effectively communicate with physicians, other health professionals, and health care teams.
- C-4: use technology to optimize communication.

Copyright © 2020 Ledford et al. This is an open-access publication distributed under the terms of the Creative Commons Attribution license.

This curriculum also contributes to learners' ability to achieve the following Entrustable Professional Activities (EPAs) for family medicine[28]:

- EPA-1: provide a usual source of comprehensive, longitudinal medical care for people of all ages.
- EPA-7: diagnose and manage chronic medical conditions and multiple comorbidities.
- EPA-15: develop trusting relationships and sustained partnerships with patients, families, and communities.
- EPA-17: in the context of culture and health beliefs of patients and families, use the best science to set mutual health goals and provide services most likely to benefit health.

Alternately, the four objectives map onto the following internal medicine PC and Interpersonal and Communication Skills (ICS) subcompetencies:

- PC-F4: with minimal supervision, manage patients with common clinical disorders seen in the practice of inpatient and ambulatory general internal medicine.
- PC-F5: with supervision, manage patients with common and complex clinical disorders seen in the practice of inpatient and ambulatory general internal medicine.
- ICS-A3: use communication skills to build a therapeutic relationship.
- ICS-A4: engage patients/advocates in shared decision-making for uncomplicated diagnostic and therapeutic scenarios.
- ICS-A5: utilize patient-centered educational strategies.
- ICS-A6: engage patient/advocates in shared decision-making for difficult, ambiguous, or controversial scenarios.
- ICS-B3: actively seek to understand patient differences and views and reflect this in respectful communication and shared decision-making with the patient and the health care team.
- ICS-D2: effectively communicate plan of care to all members of the health care team.
- ICS-D3: engage in collaborative communication with all members of the health care team.

This curriculum contributes to learners' ability to achieve the following EPAs for internal medicine:

- EPA-3: manage care of patients with chronic diseases across multiple care settings.
- EPA-4: provide age-appropriate screening and preventative care.
- EPA-11: facilitate the learning of patients, families, and members of the interdisciplinary team.
- EPA-13: improve the quality of health care at both the individual and systems levels.

The Diabetes ROADMAP curriculum has been developed iteratively, using the six-step approach to curriculum development.[29] The curriculum is designed to instruct clinicians on the process of delivering a diabetes diagnosis and targets clinicians who treat patients at risk for prediabetes or type 2 diabetes.

Methods

This report presents the evaluation of the Diabetes ROADMAP from September 2017 to March 2019. Our iterative process followed the six-step approach to curriculum development: problem identification and general needs assessment, targeted needs assessment, goals and objectives, educational strategies, implementation, and evaluation. Each phase of implementation was followed by evaluation and curriculum maintenance. See Table 1 for a summary. As this was a curricular intervention to improve diagnosis discussions, the target learner was a clinician in primary care practice. This included resident physicians, staff physicians, nurse practitioners, and physician assistants. Appendix A presents the curriculum overview.

Curricular Context

We implemented the Diabetes ROADMAP in two distinct settings: a community hospital family medicine residency (GME cohort) and a group of primary care clinics situated within an academic medical center (CME cohorts 1 and 2). All learners were primary care clinicians and had completed all requisite training. For the residency setting, we delivered the curriculum as part of regular residency education, whereas in the medical center, the curriculum was offered as continuing medical education for primary care clinicians. Note that cohorts were named for their settings, not for their participants. In the academic medical center clinics (CME cohorts), residents participated alongside their faculty and staff physicians. In the residency setting (GME cohort), one faculty member chose to participate alongside her residents.

Implementation Details for Community Hospital Residency Program

The intervention began with didactic instruction: lectures on screening and treatment guidelines and diagnosis communication in a large-group setting. We wrote the curricular content, drawing from the American Diabetes Association standards of care[15] and formative research conducted by our

Copyright © 2020 Ledford et al. This is an open-access publication distributed under the terms of the Creative Commons Attribution license.

Table 1. Iterative Development of ROADMAP Along Steps 2-6 of the Six-Step Approach to Curriculum Development[a]

Cohort	Curriculum Maintenance and Enhancement	Step 2: Needs Assessment of Targeted Learners	Step 3: Goals and Specific Measurable Objectives	Step 4: Educational Strategies	Step 5: Implementation	Step 6: Evaluation[b] and Feedback
CME cohort 1		Targeted learners: physicians, physician assistants, nurse practitioners, and clinical pharmacists practicing in primary care practices at an academic medical center	To list and describe the current ADA guidelines on screening and diagnosis of prediabetes and type 2 diabetes<br>To list and describe the treatment guidelines on prediabetes and type 2 diabetes<br>To recognize the connection between the diagnosis moment and potential for patient behavior change<br>To apply culturally competent communication to the diabetes diagnosis	Content: medical decision-making and interpersonal communication<br>Methods: didactics, role-play, simulation	Setting: September 2017 at a large academic medical center<br>Resources used: two physicians as instructors, four research team members as facilitators, 10 simulated patients	Some learners had never participated in simulation, needed more preparation for encounter.<br>Simulated patients needed more curriculum-specific training.<br>Learners needed more time.
CME cohort 2	Planned change1: local physicians as small-group facilitators, creation of facilitator guide<br>Planned change 2: more time for small-group activity and transitions<br>Planned change 3: better prepare simulated patients	Targeted learners: resident physicians and physician assistants in primary care practices at an academic medical center	To list and describe the current ADA guidelines on screening and diagnosis of prediabetes and type 2 diabetes<br>To list and describe the treatment guidelines on prediabetes and type 2 diabetes<br>To recognize the connection between the diagnosis moment and potential for patient behavior change<br>To apply culturally competent communication to the diabetes diagnosis	Content: medical decision-making and interpersonal communication<br>Methods: didactics, role-play, simulation	Setting: April 2018 at a large academic medical center<br>Resources used: two local physicians as instructors, four local physicians as facilitators, 10 simulated patients	Facilitators struggled with the small-group activity flow.<br>Needed more curriculum-specific training for simulated patients.
GME cohort	Planned change 4: fully develop a written curriculum and supporting materials<br>Planned change 5: design a version of the curriculum providing an alternate practice opportunity to the simulated encounter<br>Planned change 6: add learning objectives and content to address clinical documentation	Targeted learners: resident and staff family physicians practicing at a community hospital	To list and describe the current ADA guidelines on screening, diagnosis, and treatment of prediabetes and type 2 diabetes<br>To recognize the connection between the diagnosis moment and potential for patient behavior change<br>To demonstrate how to establish shared meaning when communicating a new diabetes diagnosis<br>To document a meaningful diabetes diagnosis in the patient health record	Content: medical decision-making, interpersonal communication, and clinical documentation<br>Methods: didactics, role-play	Setting: March 2019 at a community hospital<br>Resources used: two local physicians as instructors, two local physicians as facilitators	Curriculum was successful overall.<br>Learners were highly engaged in the small group.<br>Faculty wanted more time for role-play.

Abbreviation: ADA, American Diabetes Association.
[a]Step 1: definition of the problem—trend of patients who were unaware of the scope and severity of their diseases (prediabetes or type 2 diabetes) as well as patients identifying mixed messages from clinicians that negatively impacted patient understanding of the disease.
[b]Learner evaluation is presented in Table 3.

research team.[5-7] In this setting, four local faculty physicians acted as both teachers and facilitators. After reviewing the teaching guide, they divided the lecture content in the way they determined was most appropriate. (Appendix B presents the teaching guide; pp. 13-26 include teaching notes for each slide in the PowerPoint presentation, and p. 27 presents citations for the teaching material. Appendix C is the PowerPoint presentation.) Each faculty member also facilitated one small group. In addition to the teaching guide, they received a facilitator guide (Appendix D), which we wrote to be used without facilitator-specific training.

Copyright © 2020 Ledford et al. This is an open-access publication distributed under the terms of the Creative Commons Attribution license.

Learners in this setting (GME cohort) were resident physicians and one staff physician.

For the large-group didactic instruction, the curriculum required a large room, appropriately set up for lectures, with access to PowerPoint presentation software and a projector to display the lecture slides. The curriculum also required additional space for each small-group breakout. Small groups occurred within separate rooms to enhance the perception of a safe space in which to practice.

Following the large-group instruction, the learners divided into small groups preselected by local faculty prior to the didactics. In the small-group activity, learners practiced the steps in delivering a diabetes diagnosis while participating in, or observing, a seamless encounter. This activity provided opportunity for practicing the new communication skills because it allowed the learners to work through up to three clinical encounters in a safe place where mistakes had no impact (pp. 33-35 of Appendix D present specific step-by-step role-play instructions). Following the small-group activity, learners returned to the large-group setting for a third lecture on clinical documentation.

After the final lecture, teaching faculty described the Clinical Practice Application and Clinical Documentation Application forms. These forms provided learners a guide to talk through an active clinical encounter with a peer or a preceptor, applying the skills from the Diabetes ROADMAP (see pp. 28-29 in Appendix B). Local faculty learners were instructed to work through the application sheets when precepting patients with new diagnoses over the next 6 weeks. We gave all clinical preceptors a folder with 20 application forms and a list of participating learners pasted to the front.

Implementation Details for Primary Care Clinics Within an Academic Medical Center

Unlike the community hospital, the academic medical center had access to a local interdisciplinary simulation center that served nursing, medicine, allied health, and dentistry disciplines. The center offered simulation services including high-fidelity simulation, standardized patients, skills labs, and procedural training. This external resource allowed us to add optional simulation to the Diabetes ROADMAP curriculum. The simulation center provided a pool of trained simulated patients who matched the ethnicity and age requirements of the patient cases. For this curricular intervention, we gave each simulated patient (called practice partners in the curriculum) a character description with specific details. All patients presented at an appointment to follow up on recent lab work. Variations between cases focused on differences in culture, family history, knowledge of diabetes, and perceptions of severity. We instructed simulated patients to follow the character case with the freedom to add anything that was not included within the scope of the character they were given. (Appendix E presents all simulation resources, including 12 character cases.)

As continuing medical education, learners were staff family physicians, resident family physicians, nurse practitioners, physician assistants, and clinical pharmacists (CME cohorts 1 and 2). Note that in our system, a clinical pharmacist is often the first referral for patients as well as being the first person to have the time to discuss a new diagnosis. We included clinical pharmacists so that they would have the same training as clinicians who might code an encounter and make the referral but not explain the diagnosis. Two local physicians delivered the lectures on screening and treatment guidelines and diagnosis communication in the large-group setting. For the small-group activity, support staff and local physicians were facilitators.

Following the small group, the learners participated in the simulated clinical encounter. We instructed learners to enter the encounter to deliver a new diagnosis (p. 51 in Appendix E includes instructions for learners). Learners were allotted 15 minutes to complete the encounter. The simulation center video-recorded all encounters.

At the conclusion of all encounters, learners returned to their small-group room and were joined by the simulated patients and facilitators for group debriefs of the clinical encounters. The facilitator guided the debrief session through a list of prepared questions for the simulated patients to provide feedback to the learners (p. 52 in Appendix E provides a guide for the feedback session).

We did not use the Clinical Practice Application form with this diverse group of learners who were not all residents. Instead, in the 6 weeks following the intervention, the facilitators and the lead author reviewed the video-recorded simulated patient encounters. Using the Patient Centered Observation Form[30] (PCOF) as a guide, the team provided written feedback to each learner on his or her performance. The PCOF did not use numerical scoring; it instead focused on recording evidence of skill use. In the memos to learners, we summarized the skills relevant to the curriculum that they each demonstrated in the simulated encounter. For each learner, we included at least two successfully demonstrated skills and one skill that needed attention. One example of demonstrated skill was “You excelled at cocreating a plan with the patient.” One example of

Copyright © 2020 Ledford et al. This is an open-access publication distributed under the terms of the Creative Commons Attribution license.

recommended improvement was "At times, it appeared that you jumped between assessing patient understanding of the disease, recommending lifestyle changes, and then soliciting patient input. This disorganization can confuse the patient and disrupt your own recall in notating the visit."

Evaluation Strategy

At the community hospital, we achieved outcomes evaluation through learner reactions, a knowledge check, and survey items. (Appendix F includes all assessment tools.) At the end of the intervention, learners completed a curriculum evaluation (Appendix F, p. 44) to capture their reactions (Kirkpatrick[31] level 1). On a 1-5 scale, learners rated two Likert-type items: "This activity was a worthwhile learning experience" and "The content was delivered at an appropriate level of understanding." These two items were adapted from previous curriculum evaluation forms used in the residency. To assess learning objective 1 and Kirkpatrick level 2, we developed a short knowledge check (Appendix E, p. 41) to provide a snapshot of learners' ability to describe the screening and treatment guidelines. Rather than being an assessment of individual learners, the knowledge check gave educators a program perspective of which curriculum objectives had been met. To assess objective 2, two Likert-type items on the curriculum evaluation (Appendix E, p. 44) measured response efficacy and self-efficacy.[32] Objectives 3 and 4, both Kirkpatrick level 3, were assessed more passively: Local faculty followed up with learners in the 6 weeks after the intervention using the Clinical Practice Application and Clinical Documentation Application (Appendix B, pp. 28 and 19, respectively).

At the academic medical center, outcomes evaluation included the learner reactions from the curriculum evaluation and also learner practice as rated by the simulated patient.[33] Upon completion of the encounter (and prior to debrief), the simulated patient completed the Diabetes Health Threat Communication Questionnaire (DHTCQ).[33] The DHTCQ assessed patient perceptions of how reassuring or threatening a provider's communication was in the encounter. The DHTCQ had two subscales: reassurance and threat. The reassurance subscale assessed patients' perceptions of how well the provider communicated information about diabetes and reassured them that they could manage the disease. The threat subscale assessed patients' perceptions of the seriousness and impact of diabetes. In this evaluation, simulated patients completed the DHTCQ to capture the learners' ability to enact communication behaviors from the didactics and small-group activity. Rated on a scale of 1-7, the Likert-type items were averaged, with higher scores indicating stronger endorsement of that concept.

Results

Table 2 presents demographics for each of the learner cohorts. Of note, a number of the learners in CME cohort 1 had never participated in patient simulation. Demographics for this group of learners suggest that many learners in this cohort had completed medical training before simulation became commonplace.[34] Facilitators reported that CME learners needed more preparation for the simulated clinical encounter, such as time allotted, background of patient, and what to do upon completion of the encounter. Table 1 summarizes the feedback from each cohort.

Across the three cohorts, learners rated the curricular intervention as worthwhile ($M = 4.7$, $SD = 0.8$) and delivered at an appropriate level ($M = 4.9$, $SD = 0.4$). Table 3 presents the quantitative results of outcomes evaluation.

At the community hospital, curriculum evaluation was also assessed with a knowledge check. The mean score on the knowledge check was 4.0 out of 5, which was significantly higher than the mean score (2.3, $SD = 0.5$) of a control group of six clinicians (three residents and three faculty physicians) who did not participate in the curriculum, $F(1, 20) = 33.1$, $p < .001$. Learners also indicated high response efficacy ($M = 4.8$, $SD = 0.4$) and self-efficacy ($M = 4.8$, $SD = 0.4$) following the intervention. In the 6 weeks following the intervention, faculty tallied the use of 10 Clinical Practice Application forms with five unique learners.

Table 2. Learner Characteristics Across Three Cohorts

Characteristic	CME Cohort 1 ($n = 24$)	CME Cohort 2 ($n = 12$)	GME Cohort ($n = 17$)
Gender			
Female	13	1	7
Male	11	11	10
Years practice[a]	14.1[b]	2.5[c]	2.0[d]
Throughout practice, how frequently have you had diagnosis discussion with patients about type 2 diabetes?			
Daily	6	2	0
Weekly	14	1	5
Monthly	1	4	7
Rarely	3	5	5
Throughout practice, how frequently have you had diagnosis discussions with patients about prediabetes?			
Daily	4	2	1
Weekly	9	1	5
Monthly	7	7	9
Rarely	4	2	2

[a]Statistically significant difference among groups, $F(2, 50) = 17.62$, $p < .001$.
[b]$SD = 10.6$.
[c]$SD = 2.0$.
[d]$SD = 1.7$.

Copyright © 2020 Ledford et al. This is an open-access publication distributed under the terms of the Creative Commons Attribution license.

Table 3. Curriculum Evaluation by Cohort

Evaluation Component	CME Cohort 1 (n = 24)	CME Cohort 2 (n = 12)	GME Cohort (n = 17)
Kirkpatrick level 1: reaction[a]			
This activity was a worthwhile learning experience.	4.7 (SD = 0.9)	4.3 (SD = 0.8)	4.8 (SD = 0.4)
The content was delivered at an appropriate level of understanding.	5.0 (SD = 0.0)	4.7 (SD = 0.7)	4.9 (SD = 0.3)
Kirkpatrick level 2: learning[b]			
Total score learner knowledge check			4.0 (SD = 0.6)
Kirkpatrick level 3: behavior[c]			
DHTCQ reassurance	6.0 (SD = 0.8)	5.9 (SD = 1.0)	
DHTCQ threat	5.9 (SD = 0.7)	6.1 (SD = 1.1)	
Efficacy items[d]			
How I tell a patient about a new diagnosis will influence the patient's behavior change. (response efficacy)			4.8 (SD = 0.4)
I am confident in my ability to counsel a patient through the prediabetes/diabetes diagnosis. (self-efficacy)			4.8(SD = 0.4)

Abbreviation: DHTCQ, Diabetes Health Threat Communication Questionnaire.
[a]Scaled 1-5, with higher scores indicating more agreement.
[b]Five multiple-choice questions scored as correct/incorrect. Highest possible score was 5. The group mean score was significantly higher than 2.3 (SD = 0.5), the mean score of a group of six clinicians (three residents and three faculty physicians) who did not participate in the curriculum but completed the knowledge check, $F(1, 20) = 33.1$, $p < .001$.
[c]Scaled 1-7, with higher scores indicating stronger endorsement of concept.
[d]Scaled 1-5, with higher scores indicating more agreement.

At the academic medical center, an additional level of curriculum evaluation included simulated-patient perception of clinician communication, as measured by the DHTCQ. Across those two cohorts, simulated patients indicated high measures of both threat ($M = 6.0$, $SD = 0.8$) and reassurance ($M = 6.0$, $SD = 0.9$), which was the goal—that patients would perceive the threat of the disease but would also be reassured by the clinician.

Discussion

To address gaps we identified through patient-centered investigation of real patients' experiences with the moment of diagnosis, we developed the Diabetes ROADMAP curriculum to teach medical decision-making, interpersonal communication, and clinical documentation in the context of prediabetes and type 2 diabetes. This curriculum introduces clinicians to a method that can maximize the positive potential of the moment of diagnosis for the patient. Through various adaptations, learners here rated the curricular intervention as worthwhile and delivered at an appropriate level; recalled guidelines for screening and diagnosis; indicated high response efficacy and self-efficacy; and effected high measures of both threat and reassurance for simulated patients.

Two features of this project stand out as strengths leading to a useful end product. First is the pairing of qualitative, patient-centered research alongside the curriculum design process. This pairing ensures that the curriculum is realistic and clinically relevant. Actual quotes and realistic patient scenarios have been inserted into the curriculum, which keeps the material interesting and immediately relevant for the clinical learners. The second key feature is the iterative design process, moving through the six-step approach[29] over three iterations. The curriculum was refined and adjusted after each implementation based on feedback and observations of what did, or did not, work. Introducing the curriculum in both a large medical center and in a small community hospital allowed not only a description of potential scalability but also a concrete experience with scaling based on resources.

Throughout, we conducted formal process evaluation that allowed us to identify challenges before the next implementation. Notable challenges arose in the first iteration that we addressed in the second and third implementations. First, we were surprised to encounter learners who had never participated in simulation. We needed to provide more in-depth explanation about what simulation was like and what to expect in the encounter. Second, we discovered that simulated patients who were accustomed to interacting with undergraduate medical students and junior residents needed more training about how to interact and challenge seasoned physicians. This simulated patient training needed to reiterate the learning objectives for this topically intensive intervention. Third, we observed that the facilitators needed more scripted direction for the small-group activity.

The resulting curriculum, informed by concurrent research, is adaptable and scalable to multiple settings and learner types. Although creating a curriculum using this type of model is desirable, it is resource demanding and will likely require extramural funding—as this project did. In addition to funding, the creation of this curriculum required a setting for simulations, protected time for busy clinicians, and institutional review board–approved research projects involving several patient interviews. Implementing the curriculum will likewise require dedicated time for clinicians and space to do some level of simulation.

Copyright © 2020 Ledford et al. This is an open-access publication distributed under the terms of the Creative Commons Attribution license.

Findings here are limited by the design and setting of the intervention and evaluation. First, we evaluated the intervention at sites where local faculty were highly engaged in the design and implementation of the curriculum. We cannot predict whether faculty selecting this curriculum off the shelf will engage learners as effectively in the curricular content. Second, although the curriculum guide provides detailed instructions for the small-group activity, each small group's dynamics will affect its degree of interactivity. Third, after each implementation, we conducted a process evaluation to make improvements and adaptations to each setting. This continuous improvement process means that the three iterations collapsed for analysis here are not identical. Lastly, the design allowed for evaluation at Kirkpatrick levels 1 and 2 but only addressed level 3 in a simulated environment. Further study should include replicated testing of the final curriculum guide followed by observation of learners in clinical practice and peer review of clinical documentation of actual encounters.

We intend to further refine the curriculum as we collect additional data from implementation in different settings. In particular, we intend to expand the documentation portion of the curriculum to provide easily used, succinct, meaningful templates for use in the clinical setting. We also recognize the potential for adapting the curriculum to be used in undergraduate medical education for both knowledge and skill development.

The model used to design this curriculum on delivering the diagnosis of prediabetes and type 2 diabetes could be replicated for delivering the diagnosis of other chronic lifestyle diseases. We perceive that the moment of diagnosis of diseases such as hypertension, hyperlipidemia, and chronic obstructive pulmonary disease could also represent an opportunity to influence a patient's approach to self-management.

Appendices

A. Curriculum Overview.pdf

B. Teaching Guide.pdf

C. ROADMAP Presentation.pptx

D. Facilitator Guide.pdf

E. Simulation Resources.pdf

F. Assessment Tools.pdf

All appendices are peer reviewed as integral parts of the Original Publication.

Christy J. W. Ledford, PhD: Associate Professor, Family Medicine, Uniformed Services University of the Health Sciences; ORCID: https://orcid.org/0000-0001-5523-454X

Dean A. Seehusen, MD, MPH: Associate Dean for Graduate Medical Education and Professor of Family Medicine, Augusta University

Lauren A. Cafferty, MA: Clinical Research Coordinator, Military Primary Care Research Network, Department of Family Medicine, Uniformed Services University of the Health Sciences, and Henry M. Jackson Foundation

Heather A. Rider: Research Coordinator, Clinical Investigations Program, Mike O'Callaghan Military Medical Center

Tyler Rogers, MD: Leader and Faculty Development Fellow, Madigan Army Medical Center

Stephanie Fulleborn, MD: Resident Physician, Family Medicine, Eglin Air Force Base Family Medicine Residency

Erik Clauson, DO, CAQSM: Staff Physician, Family Medicine, Eglin Air Force Base Family Medicine Residency

Christopher C. Ledford, MD: Staff Physician, Family Medicine, Eglin Air Force Base Family Medicine Residency

Steven Trigg, MD: Resident Physician, Family Medicine, Eglin Air Force Base Family Medicine Residency

Jeremy T. Jackson: Publications Coordinator, Military Primary Care Research Network, Department of Family Medicine, Uniformed Services University of the Health Sciences, and Henry M. Jackson Foundation

Paul F. Crawford, MD: Professor of Family Medicine, Military Primary Care Research Network, Uniformed Services University of the Health Sciences, and Nellis Air Force Base Family Medicine Residency

Acknowledgments
We thank Angela Seehusen and Jasmyne Womack for their assistance in the implementation of this curriculum. We also thank Carla Fisher, who provided qualitative methods expertise to the formative phase of our research that informed this curricular content.

Funding/Support
This study was funded by the Office of the Assistant Secretary of Defense for Health Affairs through the Defense Medical Research and Development Program under Award no. FMBB100383695.

Ethical Approval
The Uniformed Services University of the Health Sciences Institutional Review Board #1 approved this study.

Disclaimer
The views expressed within this publication represent those of the authors and do not reflect the official position of the U.S. Air Force, U.S. Army, Uniformed Services University of the Health Sciences, or the U.S. Government, the Department of Defense at large.

Copyright © 2020 Ledford et al. This is an open-access publication distributed under the terms of the Creative Commons Attribution license.

References

1. Fallowfield L, Jenkins V. Communicating sad, bad, and difficult news in medicine. *Lancet*. 2004;363(9405):312-319. https://doi.org/10.1016/S0140-6736(03)15392-5
2. Berkhof M, van Rijssen HJ, Schellart AJM, Anema JR, van der Beek AJ. Effective training strategies for teaching communication skills to physicians: an overview of systematic reviews. *Patient Educ Couns*. 2011;84(2):152-162. https://doi.org/10.1016/j.pec.2010.06.010
3. Ludwig A, Lee R, Parish S, Raff A. Four-station group observed structured clinical encounter for formative assessment of communication skills for internal medicine clerks. *MedEdPORTAL*. 2016;12:10444. https://doi.org/10.15766/mep_2374-8265.10444
4. van Zuilen M, Caralis P, Granville L. Breaking bad news: a small group session teaching communication skills for delivering bad news. *MedEdPORTAL*. 2013;9:9604. https://doi.org/10.15766/mep_2374-8265.9604
5. Seehusen DA, Fisher CL, Rider HA, et al. Exploring patient perspectives of prediabetes and diabetes severity: a qualitative study. *Psychol Health*. 2019;34(11):1314-1327. https://doi.org/10.1080/08870446.2019.1604955
6. Ledford CJW, Seehusen DA, Crawford PF. Geographic and race/ethnicity differences in patient perceptions of diabetes. *J Prim Care Community Health*. 2019;10:1-5. https://doi.org/10.1177/2150132719845819
7. Ledford CJW, Seehusen DA, Crawford PF. The relationship between patient perceptions of diabetes and glycemic control: a study of patients living with prediabetes or type 2 diabetes. *Patient Educ Couns*. 2019;102(11):2097-2101. https://doi.org/10.1016/j.pec.2019.05.023
8. Sprabery L, George C, Brescia W. Diabetes mellitus: diagnosis, prevention and goals of therapy. *MedEdPORTAL*. 2011;7:7944. https://doi.org/10.15766/mep_2374-8265.7944
9. Amori RE, Simon B. A primer on diabetes mellitus: foundations for the incoming first-year resident. *MedEdPORTAL*. 2016;12:10469. https://doi.org/10.15766/mep_2374-8265.10469
10. Cannone D, Atlas M, Fornari A, Barilla-LaBarca ML, Hoffman M. Delivering challenging news: an illness-trajectory communication curriculum for multispecialty oncology residents and fellows. *MedEdPORTAL*. 2019;15:10819. https://doi.org/10.15766/mep_2374-8265.10819
11. Clay A, Ross E, Knudsen N, Chudgar S, Engle D, Grochowski C. A breaking bad news exercise to assess student competence prior to graduation. *MedEdPORTAL*. 2015;11:10015. https://doi.org/10.15766/mep_2374-8265.10015
12. Kagawa-Singer M, Dressler WW, George SM, Elwood WN. *The Cultural Framework for Health: An Integrative Approach for Research and Program Design and Evaluation*. National Institutes of Health, Office of Behavioral and Social Sciences Research; 2015.
13. Rosenstock IM. Historical origins of the health belief model. *Health Educ Monogr*. 1974;2(4):328-335. https://doi.org/10.1177/109019817400200403
14. Buysschaert M, Medina JL, Buysschaert B, Bergman M. Definitions (and current controversies) of diabetes and prediabetes. *Curr Diabetes Rev*. 2016;12(1):8-13. https://doi.org/10.2174/1573399811666150122150233
15. American Diabetes Association. *Standards of Medical Care in Diabetes—2019 abridged for primary care providers*. *Clin Diabetes*. 2019;37(1):11-34. https://doi.org/10.2337/cd18-0105
16. Ledford CJW, Villagran MM, Kreps GL, et al. “Practicing medicine”: patient perceptions of physician communication and the process of prescription. *Patient Educ Couns*. 2010;80(3):384-392. https://doi.org/10.1016/j.pec.2010.06.033
17. Knowles MS. *The Adult Learner: A Neglected Species*. 2nd ed. Gulf Publishing; 1978.
18. Quirk ME, DeWitt T, Lasser D, Huppert M, Hunniwell E. Evaluation of primary care futures: a faculty development program for community health center preceptors. *Acad Med*. 1998;73(6):705-707. https://doi.org/10.1097/00001888-199806000-00019
19. Cauffman JG, Forsyth R, Clark VA, et al. Randomized controlled trials of continuing medical education: what makes them most effective? *J Contin Educ Health Prof*. 2002;22(4):214-221. https://doi.org/10.1002/chp.1340220405
20. Seehusen DA, Miser WF. Teaching the outstanding medical learner. *Fam Med*. 2006;38(10):731-735.
21. Baile WF, Buckman R, Lenzi R, Glober G, Beale EA, Kudelka AP. SPIKES—a six-step protocol for delivering bad news: application to the patient with cancer. *Oncologist*. 2000;5(4):302-311. https://doi.org/10.1634/theoncologist.5-4-302
22. Villagran M, Goldsmith J, Wittenberg-Lyles E, Baldwin P. Creating COMFORT: a communication-based model for breaking bad news. *Commun Educ*. 2010;59(3):220-234. https://doi.org/10.1080/03634521003624031
23. Amiel GE, Ungar L, Alperin M. Using an OSCE to assess primary care physicians' competence in breaking bad news. *Acad Med*. 2000;75(5):560-561. https://doi.org/10.1097/00001888-200005000-00095
24. Adamo G. Simulated and standardized patients in OSCEs: achievements and challenges 1992-2003. *Med Teach*. 2003;25(3):262-270. https://doi.org/10.1080/0142159031000100300
25. Altshuler L, Kachur E. A culture OSCE: teaching residents to bridge different worlds. *Acad Med*. 2001;76(5):514. https://doi.org/10.1097/00001888-200105000-00045
26. Kaplonyi J, Bowles KA, Nestel D, et al. Understanding the impact of simulated patients on health care learners' communication skills: a systematic review. *Med Educ*. 2017;51(12):1209-1219. https://doi.org/10.1111/medu.13387

Copyright © 2020 Ledford et al. This is an open-access publication distributed under the terms of the Creative Commons Attribution license.

27. *The Internal Medicine Milestone Project: A Joint Initiative of the Accreditation Council for Graduate Medical Education and the American Board of Internal Medicine*. Accreditation Council for Graduate Medical Education. July 2015. Accessed August 4, 2020. https://www.acgme.org/Portals/0/PDFs/Milestones/InternalMedicineMilestones.pdf

28. EPAs, competencies, subcompetencies, and milestones: entrustable professional activities for family medicine. *Society of Teachers of Family Medicine.* Accessed August 4, 2020. https://www.stfm.org/teachingresources/resources/epascompetenciesmilestones/overview/#7136

29. Kern DE, Thomas PA, Hughes MT, eds. *Curriculum Development for Medical Education: A Six-Step Approach*. 2nd ed. Johns Hopkins University Press; 2009.

30. Keen M, Cawse-Lucas J, Carline J, Mauksch L. Using the Patient Centered Observation Form: evaluation of an online training program. *Patient Educ Couns*. 2015;98(6):753-761. https://doi.org/10.1016/j.pec.2015.03.005

31. Kirkpatrick DL. Seven keys to unlock the four levels of evaluation. *Perform Improv*. 2006;45(7):5-8. https://doi.org/10.1002/pfi.2006.4930450702

32. Smith S, Seeholzer EL, Gullett H, et al. Primary care residents' knowledge, attitudes, self-efficacy, and perceived professional norms regarding obesity, nutrition, and physical activity counseling. *J Grad Med Educ*. 2015;7(3):388-394. https://doi.org/10.4300/JGME-D-14-00710.1

33. Lawson VL, Bundy C, Harvey JN. Development of the Diabetes Health Threat Communication Questionnaire (DHTCQ). *Patient Educ Couns*. 2007;67(1-2):117-126. https://doi.org/10.1016/j.pec.2007.02.012

34. Irby DM, Wilkerson L. Educational innovations in academic medicine and environmental trends. *J Gen Intern Med*. 2003;18(5):370-376. https://doi.org/10.1046/j.1525-1497.2003.21049.x

Received: September 20, 2019
Accepted: February 25, 2020
Published: September 11, 2020

Copyright © 2020 Ledford et al. This is an open-access publication distributed under the terms of the Creative Commons Attribution license.
